# Exploratory biomarker analysis in the phase III L-MOCA study of olaparib maintenance therapy in patients with platinum-sensitive relapsed ovarian cancer

**DOI:** 10.1186/s12916-024-03409-9

**Published:** 2024-05-16

**Authors:** Huayi Li, Zikun Peng, Jianqing Zhu, Weidong Zhao, Yi Huang, Ruifang An, Hong Zheng, Pengpeng Qu, Li Wang, Qi Zhou, Danbo Wang, Ge Lou, Jing Wang, Ke Wang, Beihua Kong, Xing Xie, Rutie Yin, John Low, Abdul Malik Rozita, Lim Chun Sen, Yong Chee Meng, Kho Swee Kiong, Jihong Liu, Zhiqing Liang, Weiguo Lv, Yaping Zhu, Weiguo Hu, Wei Sun, Jingya Su, Qiqi Wang, Rongyu Zang, Ding Ma, Qinglei Gao

**Affiliations:** 1 Department of Obstetrics and Gynaecology, National Clinical Research Centre for Obstetrics and Gynaecology, Tongji Hospital, Tongji Medical College, Huazhong University of Science and Technology, 1095 Jiefang Avenue, Hankou, Wuhan, 430030 China; 2 Key Laboratory of Cancer Invasion and Metastasis (Ministry of Education), Hubei Key Laboratory of Tumour Invasion and Metastasis, Tongji Hospital, Tongji Medical College, Huazhong University of Science and Technology, 1095 Jiefang Avenue, Hankou, Wuhan, 430030 China; 3 Department of Gynaecologic Oncology, Cancer Hospital of the University of Chinese Academy of Sciences (Zhejiang Cancer Hospital), Hangzhou, China; 4 Department of Gynaecologic Oncology, Anhui Provincial Cancer Hospital, Hefei, China; 5 Department of Gynaecologic Oncology, Hubei Cancer Hospital, Wuhan, China; 6 Department of Obstetrics and Gynaecology, The First Affiliated Hospital of Xi’an Jiaotong University, Xi’an, China; 7 Department of Gynaecology, Key Laboratory of Carcinogenesis and Translational Research (Ministry of Education), Beijing Cancer Hospital, Beijing, China; 8 Department of Gynaecology Oncology, Tianjin Central Hospital of Gynaecology Obstetrics, Tianjin, China; 9 Department of Gynaecologic Oncology, Affiliated Cancer Hospital of Zhengzhou University, (Henan Cancer Hospital), Zhengzhou, China; 10 Department of Gynaecologic Oncology, Chongqing University Cancer Hospital, Chongqing, China; 11 Department of Gynaecologic Oncology, Liaoning Cancer Hospital, Shenyang, China; 12 Department of Gynaecologic Oncology, Harbin Medical University Cancer Hospital, Harbin, China; 13 Department of Gynaecologic Oncology, Hunan Cancer Hospital, Changsha, China; 14 Department of Gynaecologic Oncology, Tianjin Medical University Cancer Institute and Hospital, Tianjin, China; 15 Department of Obstetrics and Gynaecology, Qilu Hospital of Shandong University, Jinan, China; 16 Department of Gynaecologic Oncology, Women’s Hospital, School of Medicine, Zhejiang University, Hangzhou, China; 17 Department of Obstetrics and Gynaecology, West China Second University Hospital, Chengdu, China; 18 Cancer Centre @ PHKL, Pantai Hospital Kuala Lumpur, Kuala Lumpur, Malaysia; 19 Clinical Oncology Unit, Faculty of Medicine, University of Malaya, Kuala Lumpur, Malaysia; 20 Oncology Department, Hospital Sultan Ismail, Johor Bahru, Malaysia; 21 Gynaeoncology, Hospital Ampang, Ampang, Malaysia; 22 Oncology, Hospital Umum Sarawak, Kuching, Sarawak Malaysia; 23 Department of Gynaecologic Oncology, Sun Yat-sen University Cancer Centre, Guangzhou, China; 24 Department of Gynaecologic Oncology, Southwest Hospital, Third Military Medical University, Chongqing, China; 25 Department of Gynaecology, Shanghai General Hospital, Shanghai, China; 26 Fudan University Shanghai Cancer Centre and Institutes of Biomedical Sciences, Shanghai Medical College, Fudan University, Shanghai, China; 27 Department of Medical Affairs, AstraZeneca, Shanghai, China; 28 Department of Gynaecologic Oncology, Zhongshan Hospital, Fudan University, Shanghai, 200032 China

**Keywords:** Platinum-sensitive relapsed ovarian cancer, Olaparib, PD-L1 expression, *BRCA1/2*, PARP inhibitors, Homologous recombination deficiency, L-MOCA trial, Biomarker

## Abstract

**Background:**

The prospective phase III multi-centre L-MOCA trial (NCT03534453) has demonstrated the encouraging efficacy and manageable safety profile of olaparib maintenance therapy in the Asian (mainly Chinese) patients with platinum-sensitive relapsed ovarian cancer (PSROC). In this study, we report the preplanned exploratory biomarker analysis of the L-MOCA trial, which investigated the effects of homologous recombination deficiency (HRD) and programmed cell death ligand 1 (PD-L1) expression on olaparib efficacy.

**Methods:**

HRD status was determined using the ACTHRD assay, an enrichment-based targeted next-generation sequencing assay. PD-L1 expression was assessed by SP263 immunohistochemistry assay. PD-L1 expression positivity was defined by the PD-L1 expression on ≥ 1% of immune cells. Kaplan–Meier method was utilised to analyse progression-free survival (PFS).

**Results:**

This exploratory biomarker analysis included 225 patients and tested HRD status [*N* = 190; positive, *N* = 125 (65.8%)], PD-L1 expression [*N* = 196; positive, *N* = 56 (28.6%)], and *BRCA1/2* mutation status (*N* = 219). The HRD-positive patients displayed greater median PFS than the HRD-negative patients [17.9 months (95% CI: 14.5–22.1) versus 9.2 months (95% CI: 7.5–13.8)]. PD-L1 was predominantly expressed on immune cells. Positive PD-L1 expression on immune cells was associated with shortened median PFS in the patients with germline *BRCA1/2* mutations [14.5 months (95% CI: 7.4–18.2) versus 22.2 months (95% CI: 18.3–NA)]. Conversely, positive PD-L1 expression on immune cells was associated with prolonged median PFS in the patients with wild-type *BRCA1/2* [20.9 months (95% CI: 13.9–NA) versus 8.3 months (95% CI: 6.7–13.8)].

**Conclusions:**

HRD remained an effective biomarker for enhanced olaparib efficacy in the Asian patients with PSROC. Positive PD-L1 expression was associated with decreased olaparib efficacy in the patients with germline *BRCA1/2* mutations but associated with improved olaparib efficacy in the patients with wild-type *BRCA1/2*.

**Trial registration:**

NCT03534453. Registered at May 23, 2018.

**Supplementary Information:**

The online version contains supplementary material available at 10.1186/s12916-024-03409-9.

## Background

Ovarian cancer remains one of the most difficult-to-treat malignancies, with 313,959 new cases and 207,252 cancer-related deaths annually worldwide [1]. Due to the insidious onset of ovarian cancer, over 70% of patients with the disease are diagnosed with an advanced stage, thus having abysmal prognosis [1]. Though the initial response rate to platinum-based chemotherapy exceeds 70%, most patients will inevitably experience recurrence [2]. In the treatment of platinum-sensitive relapsed ovarian cancer (PSROC), the application of poly (ADP-ribose) polymerase (PARP) inhibitors is one of the most encouraging advances [3].

PARP inhibitors enable synthetic lethality in tumour cells with homologous recombination deficiency (HRD), especially those with mutated *BRCA1/2* (*BRCAm*) [4]. As the first authority-approved PARP inhibitor, olaparib prolongs progression-free survival (PFS) among patients with PSROC in Study 19 regardless of *BRCA1/2* mutation status and extends PFS among *BRCAm* patients in SOLO2 trial [5, 6]. Furthermore, OPINION trial has demonstrated that HRD-positive patients with PSROC exhibited improved responses to olaparib compared to their HRD-negative counterparts [7]. Owing to the paucity of clinical trials investigating olaparib efficacy in the Asian patients, we initiated the prospective phase III multi-centre L-MOCA study (NCT03534453) to assess the efficacy of olaparib exclusively in the Asian (mainly Chinese) patients with PSROC and its association with mutations of *BRCA1/2* and homologous recombination repair (HRR) genes [8]. The association between HRD and treatment benefits of olaparib maintenance therapy in the Asian patients with PSROC remains unclear.

In addition to eliciting targeted tumour-killing effects, PARP inhibitors could modulate tumour immune microenvironment. The efficacy of PARP inhibitors partially depends on anti-tumour immune responses [9]. DNA damage caused by PARP inhibition increases tumour mutational load and neoantigen expressions, thereby inducing anti-tumour immune responses through recruiting cytotoxic T lymphocytes (CTLs) [9]. Meanwhile, PARP inhibitors could upregulate programmed cell death ligand 1 (PD-L1) expression on tumour cells, making the combination of PARP inhibitors and immune checkpoint inhibitors a scientifically rational approach [10]. Moreover, *BRCAm* tumours display enhanced infiltration of CTLs than HRD-negative tumours [11]. Therefore, it is intriguing to decipher the effects of PD-L1 expression, an immunologic marker, on PARP inhibitors efficacy under different genotypes.

Accurate detection of genetic vulnerabilities of cancers could foster the personalised use of targeted therapies. Compared with traditional biopsy, liquid biopsy is advantageous in minimally invasive sampling, fewer contraindications and complications, and comprehensive characterization of the mutation landscape [12]. Circulating tumour DNA (ctDNA) carries the molecular aberrations of the primary tumours and effectively detects tumour-specific mutations that could be exploited by targeted therapies [13]. However, the consistency between ctDNA and tumour samples to detect molecular abnormalities in ovarian cancer, especially the evidence derived from prospective clinical trials, remain underexplored.

The prospective phase III multi-centre L-MOCA study was the first trial to evaluate olaparib efficacy and tolerability exclusively in the Asian patients with PSROC. The PFS and subgroup analysis by *BRCA1/2* and HRR mutation status were previously released [8]. In this study, the preplanned exploratory biomarker analysis of the L-MOCA trial was conducted to evaluate other potential biomarkers of treatment benefits with olaparib including HRD and PD-L1 expression and to investigate the ability of ctDNA to detect *BRCA*/HRR mutations.

## Methods

### Study design and patients

This study was the preplanned exploratory biomarker analysis of the international, prospective, open-label, single-arm, phase III L-MOCA trial, which recruited 225 patients from 28 centres in China (*N* = 22) and Malaysia (*N* = 6) between March 2018 and December 2020. The inclusion and exclusion criteria have been described in the previous report [8]. Patients aged 18 years or older, with the Eastern Cooperative Oncology Group performance status score of 0 or 1, who had received at least two courses of platinum-based chemotherapy, and had achieved either a complete or partial radiological response defined by the Response Evaluation Criteria in Solid Tumours (RECIST) guidelines were included. Patients were excluded if they had been exposed to PARP inhibitors. Eligible patients received oral olaparib (300 mg) twice daily until disease progression or unacceptable toxicity. As a committed study for registration, the L-MOCA trial has strict data quality control with the rate of source data verification reaching 100%. Approved by the national regulatory authority and the respective local ethics committees at each participating institution [(2018)-(3)-No.4 for the leading site], this study was conducted following the Good Clinical Practice guidelines and the Declaration of Helsinki. Informed consent was obtained from all individual participants included in the study. The trial protocol is presented in Additional file 1.

### Study endpoints and assessments

The primary endpoint of the L-MOCA study was investigator-assessed PFS according to RECIST 1.1 criteria, defined as the time from the date of first olaparib dose to the date of disease progression or death. Clinical and objective radiologic tumour assessments were conducted at baseline, every 12 weeks until week 72, and thereafter every 24 weeks until objective disease progression. Results of the primary analysis of PFS, along with safety, have been published [8]. Secondary endpoints included overall survival, time from first dose to second progression, and others detailed in the trial protocol (Additional file 1). This study reported the exploratory objective of exploring biomarkers in tumour tissues or blood predictive of sensitivity to olaparib including PD-L1 expression, HRD status, and *BRCA*/HRR mutation status in the ctDNA.

### Sample collection and DNA extraction

For patients with biomarker informed consent, their whole blood and tumour samples were collected at any time following diagnosis but prior to study entry. Tissues with tumour proportions ≥ 10% and necrotic proportions ≤ 50% were selected for DNA extraction. Tumour DNA and ctDNA were extracted from tumour tissue and plasma, respectively, using the QIAamp DNA Formalin-fixed paraffin-embedded (FFPE) Tissue Kit (Qiagen, Valencia, CA) and QIAamp Circulating Nucleic Acid Kit (Qiagen). Germline DNA was obtained from paired white blood cells (WBCs) via the QIAamp DNA Blood Mini Kit (Qiagen). DNA quantification was performed by a Qubit 2.0 fluorimeter (Thermo Fisher Scientific).

### *BRCA*/HRR/HRD status analysis

For each patient, we conducted paired genetic testing of tumour samples, ctDNA, and germline DNA derived from peripheral WBCs. Germline and somatic *BRCA* mutation status were established using DNA extracted from WBCs and FFPE tumour samples, respectively. To validate the *BRCA* status, 5% was set to limit the detection of *BRCA* single-nucleotide variant (SNV) or insertion/deletion (INDEL). Next-generation sequencing (NGS) on a panel of 72 genes were performed to determine the HRR alterations for both tumour and ctDNA samples. The details of HRR genes were presented in the previous report [8]. Sequence data were mapped to the reference human genome (UCSC hg19) using Burrows-Wheeler Aligner (BWA-MEM, v.0.7.10). Local alignment optimization and variant calling were performed using Genome Analysis Tool Kit (GATK, v.3.3) and VarScan v.2.4.3, respectively. Variants were filtered using the VarScan filter pipeline, where loci with depth < 100 were filtered out to eliminate benign variants. Base-calling in samples required at least 8 supporting reads for SNV and 5 supporting reads for INDEL variations. Variants with population frequency over 0.1% in the ExAC, 1000 Genomes, dbSNP, or ESP6500SI-V2 databases were grouped as single-nucleotide polymorphisms (SNPs) and excluded from further analysis. The remaining variants were annotated with ANNOVAR (2016–02-01 release) and SnpEff v.3.6. Xcavator software was applied to analyse DNA translocation. Copy number variations (CNVs) were analysed based on the depth of coverage data of capture intervals using an in-house developed algorithm. The limit of detection for CNVs was 1.5 for deletions and 2.64 for amplification, respectively. Classifications of all detected gene variants were based on the American College of Medical Genetics recommendations for standards of interpretation and reporting of sequence variants. Variants were classified as pathologic, likely pathologic, variation of uncertain significance, likely benign, or benign [14]. Deleterious *BRCA1/2* and HRR mutations included pathologic and likely pathologic mutations and were defined as detailed functional defects or changes in gene expression.

When analysing the ctDNA results, mutations from both germline and somatic origins were identified. The mutations were categorised based on their presence or absence in the WBCs. If a mutation was present in both the ctDNA and WBCs, it was considered germline; if a mutation was solely present in the ctDNA, it was deemed somatic.

ACTHRD assay, an enrichment-based targeted NGS assay, was designed to evaluate HRD status based on the detection of *BRCA1/2* gene SNVs, INDEL variations, and the extent of genome-wide loss of heterozygosity (gLOH). The analytical robustness of ACTHRD assay has been validated to be highly consistent with the Food and Drug Administration (FDA)-approved myChoice CDx [15]. The assay employs HRD panel including the whole exons of 22 HRD-related genes (*ATM*, *BARD1*, *BRIP1*, *CDK12*, *CHEK1*, *CHEK2*, *FANCI*, *FANCL*, *PPP2R2A*, *PALB2*, *RAD51C*, *RAD51D*, *RAD51B*, *RAD54L*, *ATR*, *EMSY*, *FANCA*, *FAM175A*, *NBN*, *MRE11A*, *RAD50*, and *PTEN*) and the whole exons and introns for *BRCA1* and *BRCA2*. Besides, ACTHRD assay includes over 10,000 SNPs from Agilent OneSeq for detecting LOH. The panel spans 2.8 MB of the human genome and the experimental quality control parameters are presented (Additional file 2: Table S1).

### PD-L1 expression analysis with immunohistochemistry (IHC)

The Ventana SP263 assay (Ventana Medical Systems Inc.) was adopted to assess PD-L1 expression in FFPE tumour samples. Briefly, 5 μm sections were cut and stained for SP263 on the Ventana Benchmark Ultra platform according to the standard protocol. Subsequently, stained sections were scanned using Ventana iScan HT and scored based on the percentage of tumour cells and immune cells showing membranous positivity. PD-L1 evaluation was performed by pathologists who routinely use clone SP263 in their clinical practice. For each case, the highest scoring value across the scores was used. PD-L1 expression on immune cells (IC) and tumour cells (TC), and the combined positive score (CPS) were evaluated and calculated in accordance with previous literature [16]. SP263 assay has been demonstrated to have similar levels of analytic performance with other FDA-approved PD-L1 IHC assays including 22C3 and 28–8 assays [16, 17], with advantages of high sensitivity and relatively low cost in China, which contributed to most of the study subjects and where the PD-L1 analysis was performed centrally.

### Statistical analysis

Data were summarised using descriptive statistics since the first dose of olaparib. Continuous variables were represented in mean ± standard deviation or median and interquartile range. Categorical variables were represented in frequency and percentages. PFS was estimated using the Kaplan–Meier method. All the analyses were performed using the SAS software, version 9.4.

## Results

### HRD prevalence and patients’ characteristics

**Fig. 1:**
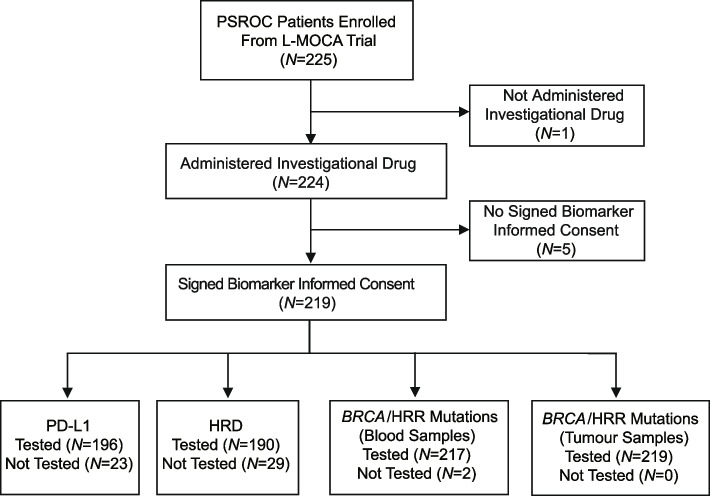
Study workflow. We included 225 patients with PSROC from the phase III L-MOCA trial, among which 219 patients with signed biomarker informed consent were included for the biomarker analysis. After strict quality control processes, PD-L1 expression and HRD status were evaluated in 196 and 190 participants, respectively. *BRCA*/HRR mutations were assessed in all the tumour samples and 217 blood samples (circulating tumour DNA). Abbreviations: PSROC, platinum-sensitive relapsed ovarian cancer; PD-L1, programmed cell death ligand 1. HRD, homologous recombination deficiency; HRR, homologous recombination repair

Between March 2018 and December 2020, 225 patients from the L-MOCA trial were enrolled and 219 eligible participants with signed biomarker informed consent were included in this preplanned exploratory biomarker analysis (Fig. 1). Specifically, 190 (86.8%) patients had residual DNA for HRD assay, including 125 (65.8%) HRD-positive patients, 26 (13.7%) HRD-negative patients, and 39 (20.5%) patients with unknown HRD status (Additional file 2: Table S2). Unknown HRD status was caused by quality check processes for tumour cellularity (*N* = 12; 6.3%), total DNA quantity and integrity (*N* = 6; 3.1%), DNA library (*N* = 8; 4.2%), bioinformatic analysis (*N* = 3; 1.6%), or SNP analysis (*N* = 10; 5.3%). Patients with HRD testing results showed a median age of 54.0 years (interquartile range, 50.0–62.0), and the majority (94.7%; *N* = 180) of them were from the China. The baseline characteristics of the participants are elaborated in Additional file 2: Table S3.

### The association between HRD and olaparib efficacy

**Fig. 2:**
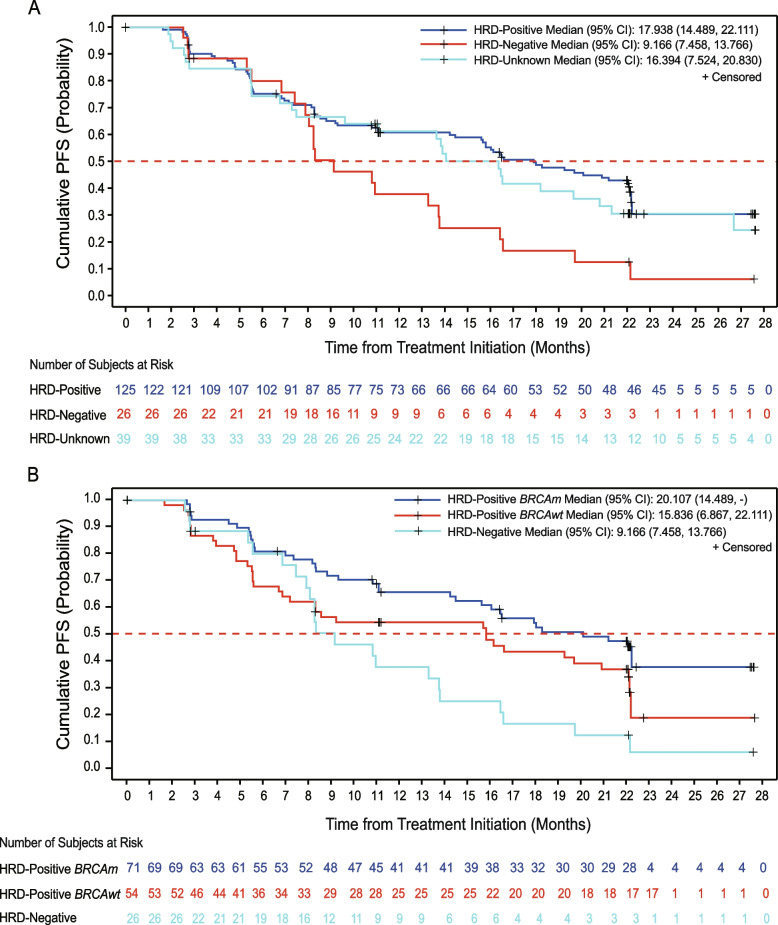
Kaplan–Meier estimates of progression-free survival in HRD/*BRCA* subgroups. **A** Kaplan–Meier plots of progression-free survival analysis in HRD-positive, HRD-negative, and HRD-unknown patients. **B** Kaplan–Meier plots of progression-free survival analysis in HRD-positive *BRCAm*, HRD-positive *BRCAwt*, and HRD-negative patients. Abbreviations: HRD, homologous recombination deficiency; 95% CI, 95% confidence interval; *BRCAm*, *BRCA1/2* mutated; *BRCAwt*, *BRCA1/2* wild type

The median PFS (mPFS) in the HRD-positive subgroup [17.9 months (95% CI: 14.5–22.1)] was prolonged compared with that in the HRD-negative subgroup [9.2 months (95% CI: 7.5–13.8); Fig. 2A]. Moreover, the 12-month and 24-month PFS rates were higher in the HRD-positive subgroup than those in the HRD-negative subgroup [12-month: 60.9% (95% CI: 51.6–69.0) versus 37.9% (95% CI: 19.3–56.5); 24-month: 30.5% (95% CI: 18.6–43.3) versus 6.3% (95% CI: 0.6–23.0); Table 1].

**Table 1:** PFS rates at 12 months and 24 months

	Number of patients (%)	12-month PFS rate, % (95% CI)	24-month PFS rate, % (95% CI)
HRD tested	190 (100)		
HRD-positive	125 (65.8)	60.9 (51.6, 69.0)	30.5 (18.6, 43.3)
HRD+ *BRCAm*	71 (56.8)	65.8 (53.1, 75.8)	37.9 (21.4, 54.4)
HRD+ *BRCAwt*	54 (43.2)	54.6 (40.3, 66.8)	19.0 (5.2, 39.5)
HRD-negative	26 (13.7)	37.9 (19.3, 56.5)	6.3 (0.6, 23.0)
HRD-unknown	39 (20.5)	61.3 (44.2, 74.6)	30.7 (16.8, 45.7)
PD-L1 expression	196 (100)		
PD-L1+	56 (28.6)	61.0 (46.6, 72.6)	29.6 (16.3, 44.2)
PD-L1–	135 (68.9)	56.1 (47.2, 64.2)	28.2 (18.5, 38.6)
PD-L1-unknown	5 (2.5)	-	-
*gBRCAm*	76 (100)		
* gBRCAm* PD-L1+	24 (31.6)	51.3 (29.5, 69.5)	23.3 (8.6, 42.3)
* gBRCAm* PD-L1–	52 (68.4)	76.3 (62.1, 85.8)	45.9 (25.1, 64.5)
*BRCAwt*	102 (100)		
* BRCAwt* PD-L1+	29 (28.4)	71.4 (50.9, 84.6)	35.7 (13.3, 59.2)
* BRCAwt* PD-L1–	73 (71.6)	41.4 (29.8, 52.7)	15.1 (6.7, 26.6)
HRRm	105 (100)		
HRRm PD-L1+	32 (30.5)	49.5 (30.6, 65.8)	22.8 (9.5, 39.7)
HRRm PD-L1–	73 (69.5)	67.4 (55.1, 77.0)	43.0 (27.4, 57.7)
HRRwt	85 (100)		
HRRwt PD-L1+	24 (28.2)	75.0 (52.6, 87.9)	36.4 (13.1, 60.5)
HRRwt PD-L1–	61 (71.8)	43.4 (30.5, 55.6)	12.6 (4.6, 24.8)
HRD-positive	122 (100)		
HRD+ PD-L1+	41 (33.6)	64.0 (46.9, 76.9)	37.8 (22.3, 53.2)
HRD+ PD-L1–	81 (66.4)	58.1 (46.4, 68.1)	28.4 (13.1, 45.8)
HRD-negative	26 (100)		
HRD– PD-L1+	5 (19.2)	60.0 (12.6, 88.2)	0 (-, -)
HRD– PD-L1–	21 (80.8)	31.9 (13.1, 52.7)	5.3 (0.4, 21.6)

Percentages are based on the number of subjects in analysis population. *Abbreviations*: *PFS* Progression-free survival, *95% CI* 95% confidence interval, *PD-L1* Programmed cell death ligand 1, g*BRCAm* Germline *BRCA1/2* mutations, *BRCAwt* *BRCA1/2* wild type, *HRRm* Mutated homologous recombination repair genes, *HRRwt* Wild-type homologous recombination repair genes, *HRD* Homologous recombination deficiency

Of the HRD-positive patients (*N* = 125), *BRCAm* patients and patients with wild-type *BRCA1/2* (*BRCAwt*) accounted for 56.8% (*N* = 71) and 43.2% (*N* = 54), respectively. Among HRD-positive patients, the *BRCAm* subgroup displayed prolonged mPFS than the *BRCAwt* subgroup [20.1 months (95% CI: 14.5–NA) versus 15.8 months (95% CI: 6.9–22.1); Fig. 2B]. Similarly, the *BRCAm* subgroup exhibited enhanced 12-month and 24-month PFS rates than the *BRCAwt* subgroup [12-month: 65.8% (95% CI: 53.1–75.8) versus 54.6% (95% CI: 40.3–66.8); 24-month: 37.9% (95% CI: 21.4–54.4) versus 19.0% (95% CI: 5.2–39.5); Table 1].

### Prevalence and patterns of PD-L1 expression

PD-L1 expression by IC, TC, and CPS was evaluated among 196 (89.5%) patients (Additional file 2: Table S4). PD-L1 was stained positive on ≥ 1% of IC in 28.6% of patients (*N* = 56), whilst PD-L1 was stained positive on < 1% of TC in the majority of patients (*N* = 180; 91.8%). For CPS, a scoring algorithm that considered both IC and TC, 25.5% of patients (*N* = 50) exhibited a CPS of ≥ 1. IHC staining showed that PD-L1 was dominantly expressed on IC (Additional file 2: Fig. S1A) and PD-L1 expression on IC contributed the most to CPS (Additional file 2: Fig. S1B, C). Therefore, PD-L1 expression on ≥ 1% of IC was set as the threshold for PD-L1 expression positivity in further analysis.

### The association between PD-L1 expression and olaparib efficacy

**Fig. 3:**
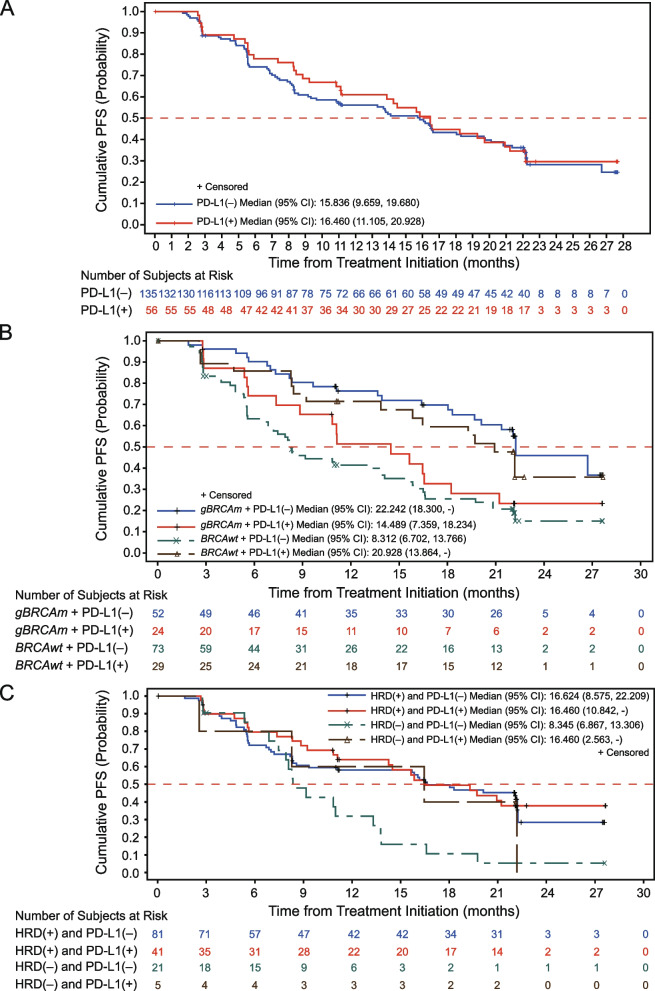
The association between PD-L1 expression and olaparib efficacy. **A** Kaplan–Meier plots of PFS analysis in PD-L1-negative (IC < 1%) and PD-L1-positive (IC ≥ 1%) patients. **B** Kaplan–Meier plots of PFS analysis in *gBRCAm* PD-L1-negative, *gBRCAm* PD-L1-positive, *BRCAwt* PD-L1-negative, and *BRCAwt* PD-L1-positive patients. **C** Kaplan–Meier plots of PFS analysis in HRD-positive PD-L1-negative, HRD-positive PD-L1-positive, HRD-negative PD-L1-negative, and HRD-negative PD-L1-positive patients. Abbreviations: PFS, progression-free survival; PD-L1, programmed cell death ligand 1; 95% CI, 95% confidence interval; *gBRCAm*, germline *BRCA1/2* mutations; *BRCAwt*, wild-type *BRCA1/2*; HRD, homologous recombination deficiency

We first investigated the effects of PD-L1 expression on olaparib efficacy in the total population. Patients with positive PD-L1 expression (IC ≥ 1%) exhibited similar PFS compared with PD-L1-negative patients (IC < 1%) [16.5 months (95% CI: 11.1–20.9) versus 15.8 months (95% CI: 9.7–19.7); Fig. 3A]. We further examined the effects of PD-L1 expression on olaparib efficacy in patients of germline *BRCA* (*gBRCA*) mutation, HRR mutation, and HRD subgroups. Intriguingly, positive PD-L1 expression was associated with shortened mPFS in patients with *gBRCA* mutations (*gBRCAm*) [14.5 months (95% CI: 7.4–18.2) versus 22.2 months (95% CI: 18.3–NA); Fig. 3B]. Conversely, positive PD-L1 expression was associated with prolonged mPFS in *BRCAwt* patients [20.9 months (95% CI: 13.9–NA) versus 8.3 months (95% CI: 6.7–13.8); Fig. 3B]. Similar effect of PD-L1 expression on olaparib efficacy was shown in HRR mutation subgroups because of the overlapping patients between *gBRCA* and HRR mutation subgroups. Among patients with HRR mutations (HRRm), PD-L1-positive patients [11.1 months (95% CI: 8.3–16.6)] exhibited shortened mPFS compared with PD-L1-negative patients [22.1 months (95% CI: 16.4–NA); Additional file 2: Fig. S2]. However, PD-L1-positive patients [20.9 months (95% CI: 13.9–NA)] demonstrated prolonged mPFS than PD-L1-negative patients [8.3 months (95% CI: 5.6–14.1)] in the patients with wild-type HRR (HRRwt) (Additional file 2: Fig. S2).

Interestingly, the effects of PD-L1 expression on olaparib efficacy disappeared in the HRD-positive subgroup. The mPFS did not differ between patients with negative and positive PD-L1 expression [16.6 months (95% CI: 8.6–22.2) versus 16.5 months (95% CI: 10.8–NA), Fig. 3C] in the HRD-positive subgroup. In the HRD-negative subgroup, positive PD-L1 expression was still associated with improved mPFS [16.5 months (95% CI: 2.6–NA) versus 8.3 months (95% CI: 6.9–13.3); Fig. 3C], but the number of PD-L1-positive patients in the HRD-negative subgroup was limited (*N* = 5). To elucidate the reasons underlying the association between PD-L1 expression and olaparib efficacy in *gBRCA* mutation subgroups and why the trend disappeared in the HRD-positive subgroup, we reviewed the demographics (Additional file 2: Tables S5, S6), pathology and extent of disease (Additional file 2: Tables S7, S8), and previously received anti-tumour therapies and responses (Additional file 2: Tables S9, S10) of PD-L1-positive and PD-L1-negative patients in g*BRCA* mutation and HRD subgroups. However, no contributing factor was found. The similar PFS between PD-L1-positive and PD-L1-negative patients in the HRD-positive subgroup could be attributed to the similar proportion of *gBRCAm* (*N* = 56; 44.8%) and *BRCAwt* patients (*N* = 60; 48.0%) in this subgroup (Additional file 2: Table S11).

### Efficacy of ctDNA in identifying *BRCA*/HRR mutation status

A total of 219 patients provided tumour samples for *BRCA*/HRR mutations profiling, resulting in 104 (47.5%) *BRCAm* patients, 114 (52.1%) *BRCAwt* patients, 124 (56.6%) HRRm patients, 94 (42.9%) HRRwt patients, and one (0.5%) patient with unknown *BRCA*/HRR mutation status (Table 2). After sequencing ctDNA samples of 217 patients, we found 92 (42.4%) *BRCAm* patients, 122 (56.2%) *BRCAwt* patients, 105 (48.4%) HRRm patients, 109 (50.2%) HRRwt patients, and three (1.4%) patients with unknown *BRCA*/HRR mutation status (Table 2). Since tissue biopsy was regarded as the reference standard, we included 213 patients with both ctDNA and tumour samples to evaluate the efficacy of ctDNA to detect *BRCA*/HRR mutation status. The overall percent agreement for ctDNA and tumour samples to detect *BRCA1/2* and HRR mutations was 94.8% (95% CI: 91.9–97.8) and 91.5% (95% CI: 87.8–95.3), respectively (Additional file 2: Table S12). The positive percentage agreement (PPA) and negative percentage agreement (NPA) of ctDNA for detecting *BRCA1/2* mutations reached 90.1% and 99.1%, respectively. Besides, ctDNA demonstrated a PPA of 86.6% and a NPA of 97.9% to detect HRR deficiencies (Additional file 2: Table S12). It was important to note that ctDNA detected all the *gBRCA1/2* mutations (91/91; 100%) but missed the majority of somatic *BRCA1/2* mutations (12/14; 85.7%) (Table 2).

**Table 2:** Summary of *BRCA* and HRR mutation testing results in ctDNA and tumour samples

	Total (*N* = 219)
*BRCA* mutation type—ctDNA from blood sample^a^	
Yes, testing results	217
Germline *BRCA1/2*	91 (41.9)
Germline *BRCA1*	64 (29.5)
Germline *BRCA2*	27 (12.4)
Somatic *BRCA1/2*	2 (0.9)
Somatic *BRCA1*	2 (0.9)
Somatic *BRCA2*	0
*BRCA1/2*	92 (42.4)
*BRCA1*	65 (30.0)
*BRCA2*	27 (12.4)
*BRCA* wild type	122 (56.2)
Unknown^b^	3 (1.4)
No	2
HRR mutation type—ctDNA from blood sample^c^	
Yes, testing results	217
HRR mutation	105 (48.4)
*BRCA1/2*	92 (42.4)
*BRCA1*	65 (30.0)
*BRCA2*	27 (12.4)
Non-*BRCA* HRR	13 (6.0)
*ATM*	2 (0.9)
*BARD1*	1 (0.5)
*BRIP1*	2 (0.9)
*CDK12*	1 (0.5)
*PALB2*	3 (1.4)
*PPP2R2A*	0
*RAD51B*	1 (0.5)
*RAD51C*	3 (1.4)
*RAD51D*	6 (2.8)
*RAD54L*	0
HRR wild type	109 (50.2)
Unknown^b^	3 (1.4)
No	2
*BRCA* mutation type—from tumour sample^a^	
Yes, testing results	219
Germline *BRCA1/2*	91 (41.6)
Germline *BRCA1*	64 (29.2)
Germline *BRCA2*	27 (12.3)
Somatic *BRCA1/2*	14 (6.4)
Somatic *BRCA1*	9 (4.1)
Somatic *BRCA2*	5 (2.3)
*BRCA1/2*	104 (47.5)
*BRCA1*	72 (32.9)
*BRCA2*	33 (15.1)
*BRCA* wild type	114 (52.1)
Unknown^d^	1 (0.5)
No	0
HRR mutation type—from tumour sample^c^	
Yes, testing results	219
HRR mutation	124 (56.6)
*BRCA1/2*	104 (47.5)
*BRCA1*	72 (32.9)
*BRCA2*	33 (15.1)
Non-*BRCA* HRR	20 (9.1)
*ATM*	3 (1.4)
*BARD1*	1 (0.5)
*BRIP1*	1 (0.5)
*CDK12*	6 (2.7)
*PALB2*	2 (0.9)
*PPP2R2A*	1 (0.5)
*RAD51B*	1 (0.5)
*RAD51C*	4 (1.8)
*RAD51D*	7 (3.2)
*RAD54L*	1 (0.5)
HRR wild type	94 (42.9)
Unknown^d^	1 (0.5)
No	0

^a^Patients with more than one mutation origin were summarised in more than one category. Patients with *BRCA* mutations of undefined origin were counted in ‘*BRCA1/2*’. Patients without mutations or with mutations other than *BRCA* of undefined origin were counted in ‘*BRCA* wild type’

^b^Circulating tumour DNA (ctDNA) samples of three patients concluded unknown *BRCA*/HRR status due to the unsuccessful next-generation sequencing library preparation (*N* = 1), low DNA quality (*N* = 1), and single-nucleotide polymorphism (SNP) mismatch between tumour and blood samples (*N* = 1)

^c^Patients with more than one mutation origin were summarised in more than one category

^d^Tumour samples of one patient concluded unknown *BRCA*/HRR status due to the test failure of both leukocyte and formalin-fixed paraffin-embedded (FFPE) tissue samples

We further investigated the association between ctDNA-detected *BRCA*/HRR mutations and olaparib efficacy. Patients with ctDNA-detected *BRCA* mutations exhibited prolonged mPFS compared with those with ctDNA-detected wild-type *BRCA* [22.2 months (95% CI: 17.9–26.7) versus 11.1 months (95% CI: 8.3–15.8); Additional file 2: Table S13]. Consistently, the mPFS in patients with ctDNA-detected HRR mutations was superior to that in patients without ctDNA-detected HRR mutations [22.2 months (95% CI: 17.9–26.7) versus 10.9 months (95% CI: 7.9–15.8); Additional file 2: Table S13].

## Discussion

In this preplanned exploratory biomarker analysis of the prospective phase III L-MOCA trial, we demonstrated that the HRD-positive patients with PSROC derived greater PFS benefits from olaparib maintenance therapy compared to the HRD-negative patients in an Asian population. Meanwhile, remarkable clinical outcomes were observed in HRD-positive patients harbouring *BRCA1/2* mutations. Positive PD-L1 expression on immune cells was associated with decreased olaparib efficacy in *gBRCAm* patients but associated with improved olaparib efficacy in *BRCAwt* patients.

The prevalence of HRD in this cohort was 65.8%, being comparable to the percentage (68.7%) reported in the real-world study among the Chinese population [18, 19] but higher than the widely accepted approximately 50% in patients with high-grade serous ovarian cancer [20]. Besides, the incidence of *gBRCA1/2* mutations was high in this cohort. The high prevalence of HRD and *gBRCA1/2* mutations may be attributable to the enrichment effects of the platinum-sensitive recurrence setting on *gBRCA1/2* mutations and HRD and the increased prevalence of *gBRCA1/2* mutations in the Chinese patients with ovarian cancer [18, 21–23]. HRD has been recommended as a predictive biomarker for PARP inhibitors benefits in prospective setting as maintenance therapy in recurrent ovarian cancer, especially in the first line [22, 24–27]. However, the evidence derived from the Asian patients has been scarce. Our study for the first time demonstrates that HRD is indeed associated with enhanced olaparib efficacy in the Asian patients with PSROC, thereby complementing and reinforcing the global evidence that links HRD with enhanced olaparib efficacy.

Currently, there is no standard threshold to define PD-L1 positivity in ovarian cancer, whether in terms of the percentage of cells stained positive or the type of cells considered (IC, TC, or both) [28]. We unravelled that in the Asian patients with PSROC, PD-L1 was prevalently expressed on IC, which aligned with previous literature stating that most PD-L1 expression was observed in inflammatory infiltrate [29] or stromal tumour-infiltrating lymphocytes [30]. However, the mechanisms underlying this pattern of PD-L1 expression in ovarian cancer remain underexplored. Using IC ≥ 1% as the threshold for PD-L1 expression positivity, we reported the previously uncharacterised association between PD-L1 expression and olaparib efficacy. Positive PD-L1 expression was associated with impaired olaparib efficacy in *gBRCAm*/HRRm patients but associated with improved olaparib efficacy in *BRCAwt*/HRRwt population. Those data indicate that genetic profiling of *gBRCA* mutations, when aided by an easily accessible PD-L1 IHC assay, might enable a more accurate estimation of olaparib efficacy in clinical practice, paving the way for clinical trials exploring the efficacy of PARP inhibitors plus PD-1/PD-L1 blockade with *gBRCA1/2* mutations and PD-L1 expression as stratification factors. However, these results warranted further investigations in future clinical trials with larger sample size.

To expound the association between PD-L1 expression on immune cells and olaparib efficacy in *gBRCA* subgroups, we hypothesized a theory of DNA damage accumulation in T cells (Additional file 2: Fig. S3). First, T cells within *gBRCAm* patients were deficient in *BRCA*-associated DNA damage repair and exhibited enhanced baseline DNA damage levels compared with *BRCA1/2*-proficient T cells. Second, PD-L1 expression on immune cells reflected IFNγ-induced adaptive regulation of PD-L1 expression and the presence of pre-existing immune responses including T cell activation [31], which has been demonstrated to accumulate DNA damage [32]. Third, PARP inhibition might lead to irreversible T cell death in activated *BRCA1/2*-deficient T cells, whilst *BRCA1/2*-proficient T cells will survive PARP inhibition even when they have been activated. Lastly, the anti-tumour efficacy of PARP inhibitors partially depends on T cell activity [9]. In summary, PD-L1 expression on immune cells was associated with decreased olaparib efficacy in *gBRCAm* patients. In *BRCAwt* patients, pre-existing T cell activation might indicate an immunoreactive niche that favours olaparib efficacy, and PD-L1 expression on immune cells was correlated with improved olaparib efficacy. Test of interaction revealed that there was statistically significant interaction between PD-L1 expression and *gBRCA1/2* mutations or HRR mutation status, whilst no significant interaction was found between PD-L1 expression and HRD status (Additional file 2: Table S14). This hypothesis needs further experimentations to enable reliable validations.

Previous studies have validated the feasibility of ctDNA in detecting mutations of HRR genes in breast and prostate cancers [33–35], but relevant studies are scarce in ovarian cancer. Our study obtained high concordance between ctDNA and tumour samples to detect *BRCA*/HRR mutations. Moreover, ctDNA-derived *BRCA*/HRR mutations could effectively inform treatment benefits with olaparib. Though ctDNA could accurately detect *gBRCA1/2* mutations, its potential to detect somatic *BRCA1/2* mutations is unclear and needs to be explored in future studies. The incapability of ctDNA to seek somatic *BRCA1/2* mutations might result from the spatial and temporal heterogeneity of somatic *BRCA1/2* mutations in a patient’s tumour and the rare chances of ovarian cancer to disseminate through the vasculature in contrast to most other types of carcinomas [36–38]. Due to the limited number of patients whose somatic *BRCA1/2* mutations were identified by ctDNA (*N* = 2), we were unable to correlate the detection of somatic *BRCA1/2* mutations with patients’ clinical characteristics.

This study had several limitations. First, due to the moderate PD-L1 expression positivity and the proportion of patients with unknown HRD status, the sample size of HRD-negative PD-L1-positive patients was limited (*N* = 5), which warranted studies with larger patient numbers. Second, the L-MOCA trial was a single-arm study without a placebo-controlled arm. Considering the proven efficacy of olaparib compared with placebo by previous randomised controlled trials in patients with PSROC, the single-arm study design minimises impact on patient survival whilst providing reliable exploratory results. Third, significant features proven to influence olaparib efficacy such as CTLs infiltration were not included in this preplanned exploratory biomarker analysis. Lastly, since only the Asian patients are included in the study, data from other ethnicities are needed to validate the findings.

## Conclusions

In conclusion, this preplanned exploratory biomarker analysis of the prospective phase III L-MOCA trial investigated the values of HRD status and PD-L1 expression to inform olaparib efficacy. Our results highlighted the importance of HRD to portend increased olaparib benefits in the Asian patients with PSROC. Meanwhile, we revealed the divergent association between PD-L1 expression and olaparib efficacy in patients with different genetic background.

### Supplementary Information

**Additional file 1.** Study protocol of the L-MOCA trial. 

**Additional file 2:** **Figure S1.** PD-L1 was mainly expressed on immune cells. **Figure S2.** The association between PD-L1 expression and olaparib efficacy in HRR mutation subgroups. **Figure S3.** The hypothesis of DNA damage accumulation in T cells. **Table S1.** ACTHRD assay experimental quality control parameters. **Table S2.** Summary of HRD testing results. **Table S3.** Summary of subject demographic and baseline factors by whether HRD was tested. **Table S4.** Summary of subjects with PD-L1 testing results. **Table S5.** Summary of demographics and baseline characteristics for different germline *BRCA* and PD-L1 subgroups. **Table S6.** Summary of demographics and baseline characteristics for different HRD and PD-L1 subgroups. **Table S7.** Summary of baseline pathology and extent of disease for different germline *BRCA* and PD-L1 subgroups. **Table S8.** Summary of baseline pathology and extent of disease for different HRD and PD-L1 subgroups.**Table S9.** Summary of previous anti-cancer therapy for ovarian cancer for different germline *BRCA* and PD-L1 subgroups. **Table S10.** Summary of previous anti-cancer therapy for ovarian cancer for different HRD and PD-L1 subgroups. **Table S11.** Summary of germline and somatic *BRCA* mutations in HRD-positive patients. **Table S12.** Concordance for detecting *BRCA*/HRR mutations in ctDNA and tumour samples. **Table S13**. PFS analysis in ctDNA-detected *BRCA* and HRR subgroups. **Table S14**. Summary of results of test of interaction.

## Data Availability

The de-identified data analysed in this study are available from the corresponding author on reasonable request.

## Abbreviations

*BRCAm*: *BRCA1/2* mutations
*BRCAwt*: Wild-type* BRCA1/2*
CNVs: Copy number variations
CPS: Combined positive score
ctDNA: Circulating tumour DNA
CTLs: Cytotoxic T lymphocytes
FDA: Food and Drug Administration
FFPE: Formalin-fixed paraffin-embedded
*gBRCAm*: Germline *BRCA1/2* mutations
gLOH: Genome-wide loss of heterozygosity
HRD: Homologous recombination deficiency
HRR: Homologous recombination repair
HRRm: Homologous recombination repair gene mutations
HRRwt: Wild-type homologous recombination repair genes
IC: Immune cells
IHC: Immunohistochemistry
INDEL: Insertion/deletion
NGS: Next-generation sequencing
NPA: Negative percentage agreement
PARP: Poly (ADP-ribose) polymerase
PD-L1: Programmed cell death ligand 1
PFS: Progression-free survival
PPA: Positive percentage agreement
PSROC: Platinum-sensitive relapsed ovarian cancer
RECIST: Response Evaluation Criteria in Solid Tumours
SNPs: Single-nucleotide polymorphisms
SNV: Single-nucleotide variant
TC: Tumour cells
WBCs: White blood cells
