# Supplementary material for: Exploratory biomarker analysis in the phase III L-MOCA study of olaparib maintenance therapy in patients with platinum-sensitive relapsed ovarian cancer
Source: BMC Med. 2024 May 16;22:199. doi: 10.1186/s12916-024-03409-9 (PMC11100112; doi:10.1186/s12916-024-03409-9)
Supplement: Supplementary file 1 — Additional file 1. Study protocol of the L-MOCA trial. [file 12916_2024_3409_MOESM1_ESM.docx]

|  | Clinical Study Protocol | |
| --- | --- | --- |
|  | Drug Substance | Olaparib |
|  | Study Code | D0816C00016 |
|  | Version | 4.0 |
|  | Date | 30th Jan 2023 |
|  |  |  |
|  | | |
| An Open Label, Single Arm, Multicentre Study to Assess the Clinical Efficacy and Safety of Lynparza (Olaparib) Tablets Maintenance Monotherapy in Platinum Sensitive Relapsed Ovarian Cancer Patients who are in Complete or Partial Response Following Platinum based Chemotherapy (L-MOCA) | | |
|  | | |
| title page | | |
|  | | |
| Sponsor: AstraZeneca Investment (China) Co. Ltd  199 Liangjing Road, Pudong New District, Shanghai, China | | |
|  | | |
|  | | |

Version History

| Version 4.0，30th Jan 2023 | |
| --- | --- |
| Main change | Reason |
| 8.5.4 Interim OS analysis are planned 3 years after last patients enrolled if 60% OS maturity is not achieved by then. | Add opportunity for earlier OS readout in trial |
| 8.5.3 Add the OS in subgroup analysis | Further analysis for OS results |
| Add the“number of prior chemotherapy lines” in subgroup analyis | Futher analysis for the Olaparib efficacy in patients with different chemotherapy lines |

This Clinical Study Protocol has been subject to a peer review according to AstraZeneca Standard procedures. The clinical study protocol is publicly registered and the results are disclosed and/or published according to the AstraZeneca Global Policy on Bioethics and in compliance with prevailing laws and regulations.

PROTOCOL SYNOPSIS

|  |
| --- |
| An Open Label, Single Arm, Multicentre Study to Assess the Clinical Efficacy and Safety of Lynparza (Olaparib) Tablets Maintenance Monotherapy in Platinum Sensitive Relapsed Ovarian Cancer Patients who are in Complete or Partial Response Following Platinum based Chemotherapy (L-MOCA) |
|  |

International Co-ordinating Investigator

| Name | Professional Title | Role in Study | Affiliation | | Email Address |
| --- | --- | --- | --- | --- | --- |
| Ding Ma | Professor | International Co-ordinating Investigator | | Tongji Medical College, Huazhong University of Science and Technology | dma@tjh.tjmu.edu.cn |
|  |  |  |  |  |  |

Study site(s) and number of patients planned

The study will be conducted in 2 Asian countries. Approximately 20-35 centres will be initiated to enrol up to approximately 220 patients.

| Study period | Timeline |  |
| --- | --- | --- |
| Estimated date of first patient enrolled | Q2 2018 |  |
| Estimated date of last patient enrolled | Q4 2018 |  |
| Estimated date of PFS analysis* | Q2 2020 |  |

* Estimated date of 132^th^ PFS event

Study design

This is a prospective, open-label, single arm, multi-centre interventional study to assess the clinical efficacy and safety of olaparib maintenance monotherapy and will be conducted in patients with platinum sensitive relapsed (PSR) high grade epithelial ovarian, fallopian tube, or primary peritoneal cancer who are in response (complete response or partial response) to platinum-based chemotherapy.

**Figure 1 Study Flow Chart**

Notes: OC ovarian cancer; PR partial response; CR complete response; PFS progression free survival; PFS2 time to second progression; TFST time to first subsequent treatment; TSST time to second subsequent treatment; OS overall survival; TDT time to discontinuation of treatment;

Patients will be assigned olaparib tablets p.o. 300 mg twice daily. They should initiate olaparib treatment within 8 weeks after their last dose of platinum-containing chemotherapy (last dose is the day of the last infusion).

Patients must have clinical and objective radiological tumour assessments according to Response Evaluation Criteria in Solid Tumors (RECIST 1.1) criteria at baseline and every 12 weeks relative to date of enrolment, until objective radiological disease progression as determined by the investigator. Patients could continue to receive olaparib for as long as determined by the investigator, until objective radiological disease progression or as long as in the investigator’s opinion they are benefiting from treatment in relation to other clinical assessments and they do not meet any other discontinuation criteria. Once a patient has discontinued olaparib she will be managed as per local clinical practice but will remain in the study and data will be collected on subsequent treatments, progression, overall survival and safety.

Objectives

| **Primary Objective:** | **Outcome Measure:** |
| --- | --- |
| To assess the efficacy of olaparib maintenance monotherapy by investigator assessed progression free survival (PFS) according to RECIST 1.1 criteria. | Time from first dosing date of olaparib to date of disease progression or death from any cause (if this occurs before disease progression) |

| **Secondary Objective:** | **Outcome Measure:** |
| --- | --- |
| To assess the efficacy of olaparib maintenance monotherapy of *BRCA*m population by investigator assessed progression free survival (PFS) according to RECIST 1.1 criteria. | Time from first dosing date of olaparib to date of disease progression or death from any cause (if this occurs before disease progression) in BRCAm population |
| To assess the clinical efficacy of olaparib maintenance monotherapy by assessment of:  a) overall survival (OS),  b) time to investigator- assessed second progression (PFS2), or death, in patients with ovarian cancer. | a) Time from first dosing date of olaparib to date of death from any cause  b) Time from first dosing date of olaparib to date of second progression event or death from any cause (if this occurs before second progression event) |
| To assess the clinical efficacy of olaparib maintenance monotherapy by assessment of  a) time to first subsequent therapy or death (TFST),  b) time to second subsequent therapy or death (TSST) and  c) time to olaparib discontinuation or death (TDT) in patients with ovarian cancer. | a) Time from first dosing date of olaparib to date of first subsequent treatment commencement or death from any cause (if this occurs before commencement of first subsequent treatment)  b) Time from first dosing date of olaparib to date of second subsequent treatment commencement or death from any cause (if this occurs before commencement of second subsequent treatment)  c) Time from first dosing date of olaparib to date of olaparib discontinuation or death from any cause (if this occurs before discontinuation of olaparib maintenance therapy) |
| **Safety Objective:** | **Outcome Measure:** |
| To evaluate the safety and tolerability of olaparib | AEs/SAEs  Collection of clinical chemistry/hematology parameters |
| **Exploratory Objective:** | **Outcome Measure:** |
| To explore biomarkers in tumor tissues or blood predictive of sensitivity/resistance to the treatment of olaparib | a) Immune-markers PD-L1 expression in tumor tissues  b) *BRCA* and HRR mutation status in the cell-free DNA from blood samples  c) HRD status in tumor samples  d) Relation between having a HRD test and patient demographic and clinical characteristics |

Target patient population

Eligible patients will be those with high grade (serous or endometrioid) epithelial ovarian cancer, primary peritoneal and/or fallopian tube cancer.

Patients must have completed 2 previous lines of platinum-based therapy (e.g., containing carboplatin or cisplatin) and platinum sensitive relapsed before entry to the study. Eligible patients must be in complete or partial response according to RECIST 1.1 criteria following the platinum-based chemotherapy prior to enrolment in the study.

*Blood sample and/or tumor samples should be provide for BRCA* (Breast Cancer Susceptibility genes) , HRR (Homologous Recombination Repair) testing when patients are enrolled in this study.

Duration of treatment

Patients should continue to receive olaparib until objective radiological disease progression according to RECIST 1.1 criteria as assessed by the investigator or as long as in the investigator’s opinion they are benefiting from treatment and they do not meet any other discontinuation criteria. Once patients have been discontinued from olaparib, other treatment options will be at the discretion of the investigator.

**Investigational product, dosage and mode of administration**

Olaparib will be available as the tablet formulation as follows:

- Tablets containing 150 mg olaparib. Patients will be administered olaparib orally at a dose of 300 mg twice daily (bid), equivalent to a total daily dose of 600 mg. The planned dose of 300 mg bid will be made up of two 150 mg tablets bid.

Treatment may be interrupted to manage adverse reactions such as nausea, vomiting, diarrhoea, and anaemia and dose reduction can be considered. For more information please refer to Section 6.8 or to the investigator brochure as appropriate.

**Statistical methods**

The sample size of approximately 220 patients is driven by the need to enrol an adequate number of patients to help understand the efficacy and safety of olaparib with the tablet formulation. Around 46 patients with *BRCA*m ovarian cancer will be enrolled assuming that *BRCA* mutation rate is 21%.

The primary analysis will be performed after the accumulation of 132 progression or death events which corresponds to 60% maturity. Assuming a median PFS of 8.4 months (Ledermann et al 2012), 132 PFS events will provide a 95% confidence interval of (7.1 m, 10.0 m) for the median PFS. Assuming a median PFS of 19.1 months (SOLO2 data, 2017 SGO oral presentation) in the *BRCA* mutated patients, 27 PFS events (60% of 46 *BRCA* mutated patients) will provide a 95% confidence interval of (13.1 m, 27.9 m) for the median PFS (Collett 1994).

Data will be summarized using descriptive statistics as appropriate. Continuous variables will be summarised by the number of observations (n), mean, standard deviation (SD), median, quartiles (Q1 and Q3), minimum, and maximum. Categorical variables will be summarised by frequency counts and percentages for each category. A Kaplan-Meier (KM) plot of PFS as well as other time-to-event outcomes will be presented. The median event times and 95% confidence intervals will be provided. Subgroup analyses will be performed according to different mutation types of *BRCA* and HRR, respectively, as well as other baseline characteristics as appropriate; these subgroups will be described in the Statistical Analysis Plan.

| TABLE OF CONTENTS | Page |
| --- | --- |

title page 1

Version History ………………………………………………………………………..2

PROTOCOL SYNOPSIS 4

TABLE OF CONTENTS 9

1. introduction 19

1.1 Background and rationale for conducting this study 19

1.1.1 Ovarian cancer and its treatment 19

1.1.2 *BRCA/HRR* mutation and HRD status in ovarian cancer 19

1.1.3 PARP inhibition as a target for *BRCA* mutation positive ovarian cancer 20

1.1.4 Pre-clinical experience 21

1.1.5 Toxicology and safety pharmacology summary 21

1.1.6 Clinical experience 22

1.2 Research hypothesis 22

1.3 Rationale for study design and doses 22

1.4 Benefit/risk and ethical assessment 23

1.5 Study Design 24

2. Study objectives 26

2.1 Primary objective 26

2.2 Secondary objectives 26

2.3 Safety objectives 27

2.4 Exploratory objectives 27

3. PATIENT SELECTION, enrolment, restrictions, discontinuation and WITHDRAWAL 27

3.1 Inclusion criteria 27

3.2 Exclusion criteria 29

3.3 Patient enrolment 31

3.4 Procedures for handling incorrectly enrolled patients 31

3.5 Methods for assigning treatment groups 31

3.6 Methods for ensuring blinding 31

3.7 Methods for unblinding 31

3.8 Restrictions 31

3.8.1 Grapefruit juice 32

3.8.2 Contraception 32

3.9 Discontinuation of investigational product 32

3.9.1 Procedures for discontinuation of a patient from investigational product 32

3.10 Criteria for withdrawal 33

3.10.1 Screen failures 33

3.10.2 Withdrawal of the informed consent 34

3.11 Discontinuation of the study 34

4. Study plan and timing of procedures 34

4.1 Study schedule 34

4.2 Enrolment/screening period 41

4.3 Treatment period 41

4.4 Follow-up period 41

4.4.1 Follow-up 30 days after last dose of investigational product (IP) 41

4.4.2 Survival 41

4.4.3 Second progression (PFS2) 42

4.4.4 Patient management post primary analysis 42

4.4.5 Patient management post final analysis 42

5. study assessments 43

5.1 Efficacy assessments 43

5.1.1 CT and MRI scans tumour assessments (Modified RECIST 1.1) 43

5.1.2 Tumour evaluation 44

5.2 Safety assessments 45

5.2.1 Laboratory safety assessments 45

5.2.1.1 Coagulation 46

5.2.1.2 Bone marrow or blood cytogenetic samples 46

5.2.1.3 Disease specific tumour marker samples (CA-125) 47

5.2.2 Physical examination 47

5.2.3 ECG 47

5.2.4 Vital signs 47

5.2.4.1 Pulse and blood pressure 48

5.2.4.2 Body temperature 48

5.2.5 Other safety assessments 48

5.2.5.1 Serum or urine pregnancy test 48

5.3 Pharmacokinetics 48

5.3.1 Collection of samples 48

5.3.2 Determination of drug concentration 48

5.3.3 Storage and destruction of pharmacokinetic samples 48

5.4 Pharmacodynamics 48

5.4.1 Collection of samples 48

5.4.2 Storage, re-use and destruction of pharmacodynamic samples 48

5.5 Pharmacogenetics 49

5.5.1 Collection of pharmacogenetic samples 49

5.5.2 Storage, re-use and destruction of pharmacogenetic samples 49

5.6 Biomarker analysis 49

5.6.1 Blood sample for germline *BRCA* and other non-*BRCA* HRR gene panel testing 49

5.6.2 Tumour sample for somatic *BRCA* and other non-*BRCA* HRR gene panel testing 49

5.6.3 Exploratory blood sample for circulating tumour DNA analysis 49

5.6.4 Tumour sample for exploratory inmmunohistochemistry analysis of PD-L1 expression 49

5.6.5 Exploratory blood and tumor samples for HRD testing 50

5.6.6 Withdrawal of informed consent for provided biological samples 50

6. safety reporting and medical management 50

6.1 Definition of adverse events 50

6.1.1 Olaparib adverse events of special interest 51

6.2 Definitions of serious adverse event 51

6.3 Recording of adverse events 51

6.3.1 Time period for collection of adverse events 51

6.3.1.1 Adverse events after the 30 day follow up period 52

6.3.2 Follow-up of unresolved adverse events 52

6.3.3 Variables 52

6.3.4 Causality collection 53

6.3.5 Adverse events based on signs and symptoms 54

6.3.6 Adverse events based on examinations and tests 54

6.3.7 Hy’s Law 54

6.3.8 Disease progression 55

6.3.9 New cancers 55

6.3.10 Lack of efficacy 55

6.3.11 Deaths 55

6.4 Reporting of serious adverse events 56

6.5 Overdose 56

6.6 Pregnancy 57

6.6.1 Maternal exposure 57

6.6.2 Paternal exposure 58

6.7 Medication Error 58

6.8 Management of IP related toxicities 59

6.8.1 Management of haematological toxicity 59

6.8.1.1 Management of anaemia 59

6.8.1.2 Management of neutropenia, leukopenia and thrombocytopenia 60

6.8.1.3 Management of of prolonged haematological toxicities while on study treatment 61

6.8.2 Management of non-haematological toxicity 61

6.8.2.1 Management of new or worsening pulmonary symptoms 61

6.8.2.2 Management of nausea and vomiting 62

6.8.2.3 Interruptions for intercurrent non-toxicity related events 62

6.9 Study governance and oversight 63

6.9.1 Steering Committee 63

6.9.2 Data Monitoring Committee 63

6.9.3 Scientific Advisory Committee 63

7. investigational product and other Treatments 63

7.1 Identity of investigational product(s) 63

7.2 Dose and treatment regimens 63

7.3 Labelling 65

7.4 Storage 65

7.5 Compliance 65

7.6 Accountability 65

7.7 Concomitant and other treatments 66

7.8 Post Study Access to Olaparib 69

8. Statistical analyses by Astrazeneca 69

8.1 Statistical considerations 69

8.2 Sample size estimate 69

8.3 Definitions of analysis sets 69

8.4 Outcome measures for analyses 70

8.4.1 Calculation or derivation of efficacy variable(s) 70

8.4.2 Primary endpoint 70

8.4.3 Secondary endpoints 70

8.4.4 Calculation or derivation of safety variable(s) 71

8.4.5 Other significant adverse events (OAE) 72

8.5 Methods for statistical analyses 72

8.5.1 Analysis of the primary variable (s) 72

8.5.2 Analysis of the secondary variable(s) 72

8.5.3 Subgroup analysis (if applicable) 73

8.5.4 Interim analysis 73

8.5.5 Exploratory analysis (if applicable)) 73

9. STUDY AND DATA MANAGEMENT BY ASTRAZENECA 73

9.1 Training of study site personnel 73

9.2 Monitoring of the study 74

9.2.1 Source data 74

9.2.2 Study agreements 74

9.2.3 Archiving of study documents 75

9.2.4 Deviation from the clinical study protocol 75

9.3 Study timetable and end of study 75

9.4 Data management by AstraZeneca 76

10. Ethical and regulatory requirements 77

10.1 Ethical conduct of the study 77

10.2 Patient data protection 77

10.3 Ethics and regulatory review 77

10.4 Informed consent 78

10.5 Changes to the protocol and informed consent form 78

10.6 Audits and inspections 79

11. LIST OF References 79

LIST OF TABLES

Table 1 Study Schedule Screening 34

Table 2 Study Schedule – On Study Treatment and Discontinuation 36

Table 3 Study Schedule – Follow up Post Discontinuation of Study Treatment 39

Table 4 Laboratory Safety Variables 46

Table 5 Management of anaemia 59

Table 6 Management of neutropenia, leukopenia and thrombocytopenia 60

Table 7 Dose reductions for study treatment 63

LIST OF FIGURES

Figure 1 Study Flow Chart 25

LIST OF APPENDICES

Appendix A Additional Safety Information 83

Appendix B Actions Required in Cases of Increases in Liver Biochemistry and Evaluation of Hy’s Law 85

Appendix C Acceptable Birth Control Methods 90

List of abbreviations and definition of terms

The following abbreviations and special terms are used in this study Clinical Study Protocol.

| Abbreviation or special term | Explanation |
| --- | --- |
| ACMG | American College of Medical Genetics and Genomics |
| AE | Adverse event |
| AESI | Adverse events of special interest |
| ALP | Alkaline Phosphatase |
| ALT | Alanine Aminotransferase |
| AML | Acute Myeloid Leukemia |
| ANC | Absolute neutrophil count |
| APTT | Activated Partial Thromboplastin Time |
| AST | Aspartate Aminotransferase |
| Bid | Twice daily |
| BP | Blood Pressure |
| BRCA | Breast cancer susceptibility gene |
| BUN | Blood Urea Nitrogen |
| CHO | Chinese Hamster Ovary |
| CI | Confidence Interval |
| CR | Complete Response |
| CrCl | Creatinine clearance |
| CRF | Case Report Form |
| CRO | Contract Research Organisation |
| eCRF | Electronic Case Report Form |
| CSA | Clinical Study Agreement |
| CSR | Clinical Study Report |
| CT | Computed Tomography |
| CTCAE | Common Terminology Criteria for Adverse Event |
| DAE | Discontinuation of Investigational Product due to Adverse Event |
| DCIS | Ductal carcinoma *in situ* |
| DCO | Date of Discontinuation |
| DMP | Data Management Plan |
| DNA | Deoxyribonucleic acid |
| DoR | Duration of Response |
| DSB | Double Strand Breaks |
| dUCBT | Double umbilical cord blood transplantation |
| EC | Ethics Committee, synonymous to Institutional Review Board (IRB) and Independent Ethics Committee (IEC) |
| ECG | Electrocardiogram |
| ECOG | Eastern Cooperative Oncology Group: a performance status using scales and criteria to assess how a patient’s disease is progressing |
| EDC | Electronic Data Capture |
| ENGOT | European Network of Gynaecological Oncological Trial Groups |
| EU | European Union |
| FACIT | Functional Assessment of Chronic Illness Therapy |
| FACT-O | Functional Assessment of Cancer Therapy-Ovarian |
| FAS | Full Analysis Set |
| FFPE | Formalin fixed, paraffin embedded |
| FSH | Follicle Stimulating hormone |
| FSI | First Subject In (enrolled) |
| gBRCAm | Germline BReast CAncer Gene mutation |
| GCP | Good Clinical Practice |
| G-CSF | Granulocyte colony stimulating factor |
| Hb | Haemoglobin |
| HIV | Human Immunodeficiency Virus |
| HR | Hazard Ratio |
| HRCT | High Resolution Computed Tomography |
| HRD | Homologous Recombination Deficiency |
| HRT | Hormone Replacement Therapy |
| HRR | Homologous Recombination Repair |
| IB | Investigator’s Brochure |
| ICH | International Conference on Harmonisation |
| INR | International Normalised Ratio |
| International Co‑ordinating investigator | If a study is conducted in several countries the International Co-ordinating Investigator is the Investigator co-ordinating the investigators and/or activities internationally. |
| IP | Investigational Product |
| IVRS | Interactive Voice Response System |
| IWRS | Interactive Web Response System |
| LH | Luteinizing Hormone |
| MCV | Mean Cell Volume |
| MDS | Myelodysplastic Syndrome |
| MRI | Magnetic Resonance Imaging |
| NCI | National Cancer Institute |
| NE | Not Evaluable |
| NTL | Non-target Lesion |
| OAE | Other Significant Adverse Event |
| ORR | Objective Response Rate |
| OS | Overall Survival |
| PARP | Poly-adenosine 5’diphosphoribose (ADP) Polymerase |
| PD | Progression of Disease |
| PFS | Progression Free Survival |
| PFS2 | Time to second progression |
| PI | Principal Investigator |
| p.o. | Administered by mouth |
| PPS | Per-protocol analysis set |
| PR | Partial Response |
| PRO | Patient Reported Outcome |
| PSR | Platinum Sensitive Relapsed |
| QoL | Quality of Life |
| RECIST | Response Evaluation Criteria in Solid tumours. This study will use RECIST version 1.1. |
| SAE | Serious adverse event |
| SAP | Statistical Analysis Plan |
| SD | Stable Disease |
| sBRCAm | Somatic BReast CAncer gene mutation |
| SGOT | Serum Glutamic Oxaloacetic Transaminase |
| SGPT | Serum Glutamic Pyruvate Transaminase |
| SSB | Single Strand Breaks |
| TDT | Time to Discontinuation of Treatment |
| TFST | Time to First Subsequent Treatment |
| TL | Target Lesion |
| TOI | Trial Outcome Index |
| TSST | Time to Second Subsequent Treatment |
| ULN | Upper Limit of Normal |
| US | United States |
| WBDC | Web Based Data Capture |
| Wt | Wild type (patients without evidence of *BRCA* 1 or *BRCA* 2 deleterious or suspected deleterious mutations) |

# introduction

## 1.1 Background and rationale for conducting this study

### Ovarian cancer and its treatment

Ovarian cancer is the fifth most common cause of death from cancer in women (Colombo et al 2010; NCCN Clinical Practice Guidelines in Oncology). In China, approximately 52,100 new cases of ovarian cancer and approximately 22,500 deaths are reported annually, ranking ovarian cancer as the leading cause of death from gynaecological cancer (Chen et al, 2015). In the United States (US), nearly 80% of the patients are diagnosed with advanced disease (regional or distant), while only 15% are diagnosed at the localized stage (Howlader et al, 2014). Approximately 44% of women diagnosed with ovarian cancer (at any stage) survive 5 years from diagnosis, while among those diagnosed at the distant stage of the disease 5-year survival is ~27% (Siegel et al, 2014). In China, 5-year survival rate is nearly 39% (Zeng et al, 2015).

The standard therapy for advanced ovarian cancer consists of radical debulking surgery followed by post-operative platinum-based first-line chemotherapy. Since 1996, platinum and paclitaxel combination therapy has become the standard-of-care first-line chemotherapy regimen (McGuire et al 1996). Worldwide, the use of carboplatin has replaced that of cisplatin because of carboplatin’s superior tolerability profile together with equal effectiveness. However, the success of this approach is limited and approximately 70% of patients fail to achieve complete responses, or eventually relapse, after a varying disease-free interval.

Should a relapse or a progression after first-line therapy occur, cure of the disease is rarely possible. In these cases, second-line chemotherapy is usually offered, with a palliative intent. Recurrence-free interval influences the choice of possible second-line chemotherapy, specifically the value of platinum re-exposure (Blackledge et al 1989, Gore et al 1990, Markman et al 1991) and is of prognostic importance for the ongoing course of the illness (Eisenhauer et al 1997). Patients with ovarian cancer who developed recurrence >6 months after completion of first line platinum chemotherapy are characterised as having platinum-sensitive disease.

For patients with platinum-sensitive disease, response rates of 20% to 30% may be seen with platinum re-treatment in those with a platinum-free interval of 6 to 12 months. In those patients with treatment-free intervals of >12 months, response rates in the range of 30% to 70% may be seen, and some patients may benefit from durable second remissions. Carboplatin in combination with gemcitabine, pegylated liposomal doxorubicin or paclitaxel represents the main treatment options for patients with relapsed platinum-sensitive disease, being repeated as long as the patients remain platinum sensitive.

### *BRCA/HRR* mutation and HRD status in ovarian cancer

An important risk factor for ovarian cancer is genetic predisposition with *BRCA*1 or *BRCA*2 mutations (i.e., g*BRCA*m) which account for the majority of hereditary ovarian cancers. If a lifetime risk for ovarian cancer among women in the general population is estimated to be 1.4 percent (14 out of 1,000), a woman with *BRCA*1 or *BRCA*2 deleterious mutation has a lifetime risk of 15 to 40 percent (150–400 out of 1,000) (NCI; *BRCA*1 and *BRCA*2: Cancer Risk and Genetic Testing). *BRCA* mutated ovarian cancer patients can also develop ovarian cancer earlier in their life than those without the mutation. Deficiency in *BRCA* ultimately leads to the accumulation of genetic alterations as a result of the failure of cells to arrest and repair deoxyribonucleic acid (DNA) damage or to undergo apoptosis, resulting in tumourigenesis. If all ovarian cancer patients underwent g*BRCA* testing, current estimates indicate that 14% of the overall ovarian cancer population would have g*BRCA*1/2 mutations, and the proportion of patients with g*BRCA* mutations may be as high as 22% in patients with high grade epithelial ovarian cancer. In addition, a population of ovarian cancer patients whose tumours harbour *BRCA*1 and *BRCA*2 mutations that are not detected in the germline (~7%) also exist and are defined as somatic *BRCA* mutations (s*BRCA*m).

Patients with *BRCA*-mutated ovarian cancer currently have identical treatment options as sporadic ovarian cancer patients. They seem to have a better prognosis compared with the overall relapsed ovarian cancer patient population but the pattern of disease is similar, with patients eventually dying from their disease. Ovarian cancer patients with *BRCA* mutation represent a small, well defined and medically recognised subpopulation.

Throughout the protocol the term ‘*BRCA* mutation’ is used to refer to a *BRCA1* or *BRCA2* mutation (detected either in the germline or in the tumour) classified as ‘deleterious’ or ‘suspected deleterious’ in accordance with the American College of Medical Genetics and Genomics (ACMG) recommendations for standards for interpretation and reporting of sequence variants (Richards et al 2008).

Germline or somatic mutations in HRR genes are present in almost one-third of ovarian carcinomas, including both serous and non-serous histologies. Somatic *BRCA*1/2 mutations and mutations in other HRR genes have a similar positive impact on overall survival and platinum responsiveness as germline *BRCA*1/2 mutations. The similar rate of HRR mutations in non-serous carcinomas supports their inclusion in PARP inhibitor clinical trials. 31% of ovarian carcinomas had a deleterious germline (24%) and/or somatic (9%) mutation in one or more of the 13 HR genes: *BRCA*1, *BRCA*2, ATM, BARD1, BRIP1, CHEK1, CHEK2, FAM175A, MRE11A, NBN, PALB2, RAD51C, and RAD51D. The presence of germline and somatic HRR mutations was highly predictive of primary platinum sensitivity and improved overall survival, with median overall survival 66 months in germline HRR mutation carriers, 59 months in cases with a somatic HR mutation, and 41 months for cases without an HRR mutation (Pennington et al 2014).

Approximately 50% of epithelial ovarian cancers (EOC) exhibit homologous recombination deficiency (HRD) due to genetic alterations of HR pathway genes, promoter methylation, and other undefined reasons. Defective HR is an important therapeutic target in EOC as exemplified by the efficacy of platinum analogues in this disease, as well as the advent of PARP inhibitors. (Konstantinopoulos et al 2015)

### PARP inhibition as a target for *BRCA* mutation positive ovarian cancer

Investigators should be familiar with the current olaparib (AZD2281) Investigator Brochure (IB).

Olaparib (AZD2281, KU-0059436) is a potent Polyadenosine 5’diphosphoribose [poly (ADP ribose)] polymerisation (PARP) inhibitor (PARP-1, -2 and -3) that is being developed as an oral therapy, both as a monotherapy (including maintenance) and for combination with chemotherapy and other anti-cancer agents.

PARP inhibition is a novel approach to targeting tumours with deficiencies in DNA repair mechanisms. PARP enzymes are essential for repairing DNA single strand breaks (SSBs). Inhibiting PARPs leads to the persistence of SSBs, which are then converted to the more serious DNA double strand breaks (DSBs) during the process of DNA replication. During the process of cell division, DSBs can be efficiently repaired in normal cells by homologous recombination repair (HRR). Tumours with HR deficiencies (HRD), such as ovarian cancers in patients with *BRCA* mutations, cannot accurately repair the DNA damage, which may become lethal to cells as it accumulates. In such tumour types, olaparib may offer a potentially efficacious and less toxic cancer treatment compared with currently available chemotherapy regimens.

*BRCA*1 and *BRCA*2 defective tumours are intrinsically sensitive to PARP inhibitors, both in tumour models in vivo (Rottenberg et al 2008, Hay et al 2009) and in the clinic (Fong et al 2009). The mechanism of action for olaparib results from the trapping of inactive PARP onto the single-strand breaks preventing their repair (Helleday 2011; Murai et al 2012). Persistence of SSBs during DNA replication results in their conversion into the more serious DNA DSBs that would normally be repaired by HR repair. Olaparib has been shown to inhibit selected tumour cell lines in vitro and in xenograft and primary explant models as well as in genetic *BRCA* knock out models, either as a stand-alone treatment or in combination with established chemotherapies.

### Pre-clinical experience

The pre-clinical experience is fully described in the current version of the olaparib Investigational Brochure (IB).

### Toxicology and safety pharmacology summary

Olaparib has been tested in a standard range of safety pharmacology studies e.g., dog cardiovascular and respiratory function tests, and the rat Irwin test. There were no noticeable effects on the cardiovascular or respiratory parameters in the anaesthetised dog or any behavioural, autonomic or motor effects in the rat at the doses studied.

Rodent and dog toxicology studies have indicated that the primary target organ of toxicity is the bone marrow with recovery seen following withdrawal of olaparib. Ex vivo studies have confirmed that olaparib is cytotoxic to human bone marrow cells.

Olaparib was not mutagenic in the Ames test but was clastogenic in the Chinese hamster ovary (CHO) chromosome aberration test in vitro. When dosed orally, olaparib also induced micronuclei in the bone marrow of rats. This profile is consistent with the potential for genotoxicity in man.

Reproductive toxicology data indicate that olaparib can have adverse effects on embryofoetal survival and development at dose levels that do not induce significant maternal toxicity.

Further information can be found in the current version of the olaparib IB.

### Clinical experience

Clinical experience with olaparib is fully described in the current version of the olaparib IB.

## Research hypothesis

Olaparib administered as monotherapy in the maintenance setting improves progression free survival compared to placebo in patients with relapsed ovarian cancer who have a complete or partial response to platinum-based chemotherapy.

## Rationale for study design and doses

Based on the results of a pivotal phase II trial (D0810C00019), described in more detail below, olaparib as the capsule formulation at a daily recommended dose of 400mg twice daily (a total daily dose of 800mg) has been approved in EU as monotherapy for the maintenance treatment of adult patients with platinum sensitive relapsed (PSR) *BRCA* mutated (germline and/or somatic) high grade epithelial ovarian, fallopian tube, or primary peritoneal cancer who are in response (complete response or partial response) to platinum-based chemotherapy. In this phase II trial, Olaparib as maintenance treatment significantly improved progression-free survival among patients with platinum-sensitive, relapsed, high-grade serous ovarian cancer, not limited to *BRCA*m population.

The phase II study D0810C00019 is a randomised, double-blind, placebo-controlled study to evaluate maintenance treatment with olaparib (capsule formulation) in patients with platinum-sensitive relapsed high grade epithelial ovarian cancer who had received ≥2 previous platinum regimens and were in partial or complete response following their last platinum-containing regimen. The primary endpoint was investigator-assessed PFS. In total, 265 patients were randomised to olaparib 400 mg bid (136) or placebo (129). The primary analysis was carried out following 153 PFS events and demonstrated that maintenance treatment with olaparib led to a significant PFS improvement vs placebo [hazard ratio (HR) 0.35 (95% confidence interval (CI) 0.25 -0.49); median: 8.4 vs 4.8 months; p<0.00001] (Ledermann et al 2012). A subgroup analysis (pre-specified in the [SAP]) suggested that olaparib may lead to a greater clinical benefit in patients with a known germline *BRCA*m. g*BRCA*m status was determined retrospectively for all consenting patients (n=166) using blood samples taken before randomisation. s*BRCA*m status was determined from archival tumour samples of 196 patients. Since g*BRCA*wt patients may develop somatic tumour *BRCA* mutations, efficacy analyses were performed by known g*BRCA* mutation status and known total *BRCA* mutation status. g*BRCA*m patients had the greatest PFS benefit with olaparib maintenance vs placebo (HR, 0.17; 95% CI 0.09-0.31; median: 11.2 vs 4.1 months; P<0.001). The PFS benefit was consistent when s*BRCA*m patients were included to include an overall of 136 patients with *BRCA*m disease (HR, 0.18; 95% CI 0.11-0.31; median: 11.2 vs 4.3 months; P<0.0001). In an updated analysis (Ledermann et al 2016), an OS (77% maturity) advantage was seen with maintenance olaparib versus placebo in all patients (HR 0.73 [95% CI 0.55–0.96]; median overall survival was 29.8 months [95% CI 26.9–35.7] for those treated with olaparib vs 27.8 months [24.9–33.7] for those treated with placebo), and in patients with *BRCA*m (HR 0.62 [95% CI 0.41–0.94]; 34.9 months [95% CI 29.2–54.6] vs 30.2 months [23.1–40.7]). 11 (15%) of 74 patients with *BRCA*m received maintenance olaparib for 5 years or more. OS nominal p=0.025, which did not meet the required threshold for statistical significance [p<0・0095], however 30% g*BRCA*m placebo patients received a subsequent PARP inhibitor, confounding the OS data in this subgroup. Olaparib tolerability was similar in *BRCA*m patients and the overall population.

The phase II study D0810C000019 demonstrated the efficacy of olaparib maintenance when using the capsule formulation (8 capsules twice daily). A more patient friendly tablet formulation (2 tablets twice daily) has been developed. Study D0816C00002 (SOLO2) was a Phase III randomised, double-blind, placebo-controlled study of olaparib 300 mg bd (tablet formulation) as maintenance therapy in high grade ovarian cancer patients who were in response to their most recent platinum-based chemotherapy after ≥2 lines of treatment and who had a deleterious or suspected deleterious g*BRCA*m. The phase III confirmatory trial is conducted with the new tablet formulation. Results from the trial demonstrate a clinically-meaningful and statistically-significant improvement of progression-free survival (PFS) among patients treated with Lynparza compared to placebo and provide additional evidence to support the potential use of Lynparza in this patient population. Olaparib achieved a highly statistically significant and clinically meaningful prolongation in PFS compared with placebo, by Investigator assessment (HR, 0.30; 95% CI 0.22-0.41; median: 19.1 vs 5.5 months; P<0.0001); BICR assessment of PFS (HR, 0.25; 95% CI 0.18-0.35; median: 30.2 vs 5.5 months; P<0.0001) confirmed these results.

Further information is provided in the IB.

The current study will allow patients to complete their platinum-containing regimen prior to enrolment and then initiate maintenance treatment with olaparib. This study is to explore the use of Olaparib as maintenance monotherapy in PSR patients and to provide valuable further insight into patients’ benefit and tolerability.

For the current study, it is expected that participating patients may know their *BRCA* and HRR status after enrolment and will have undergone counselling in accordance with local hospital practice.

## Benefit/risk and ethical assessment

Olaparib is considered to have a positive benefit-risk profile in this study target population of relapsed platinum sensitive ovarian cancer patients based on the randomised phase II data in the same patient population demonstrating a significant and clinically meaningful prolongation of progression free survival, with an acceptable tolerability profile for use in the maintenance setting. Platinum-containing therapy is considered the treatment of choice for patients with recurrent platinum sensitive ovarian cancer, however the duration of response (DoR) and the prolongation of the progression free interval are usually brief and these chemotherapy regimens cannot be continued until progression as they are associated with neurological, renal and haematological toxicity and cannot generally be tolerated for more than about 6 cycles. Since chemotherapy is not a viable treatment option in the maintenance setting, there is a need for a maintenance treatment (following completion of chemotherapy) with adequate tolerance profile that can be taken until disease progression to extend the progression free interval in this patient population.

The current study design will allow patients to complete their platinum-containing regimen as per normal clinical practice prior to enrolment. The use of olaparib as a maintenance therapy after completion of chemotherapy may provide further benefit to patients in terms of prolongation of the progression free interval, increasing the interval between lines of chemotherapy, delaying further hospitalisation and the cumulative toxicities associated with chemotherapy. The tablet formulation is considered to be a more patient friendly formulation for long term use requiring patients to take up to 2 tablets twice daily as compared to the capsule formulation requiring 8 capsules twice daily. Considering that ovarian cancer is the leading cause of death from gynaecological tumours in the world, the clinically meaningful efficacy benefit of olaparib in the *BRCA* mutated and PSR patient population and the known tolerability profile of olaparib monotherapy, the current study is considered to have a positive benefit-risk profile. The known risks to olaparib monotherapy in terms of safety and tolerability are considered justifiable. Refer to the current IB for a complete description of the safety and tolerability profile.

## Study Design

This is a prospective, open-label, single arm, multi-centre interventional study to assess the clinical efficacy and safety of olaparib maintenance monotherapy as the tablet formulation in PSR high grade (serous or endometrioid) epithelial ovarian cancer patients (including patients with primary peritoneal and / or fallopian tube cancer).

Figure 1 Study Flow Chart

Notes: OC ovarian cancer; PR partial response; CR complete response; PFS progression free survival; PFS2 time to second progression; TFST time to first subsequent treatment; TSST time to second subsequent treatment; OS overall survival; TDT time to discontinuation of treatment;

Patients will be assigned to olaparib tablets p.o. 300 mg twice daily. They should initiate olaparib treatment within 8 weeks after their last dose of chemotherapy (last dose is the day of the last infusion).

Patients must have clinical and objective radiological tumour assessments according to RECIST 1.1 criteria at baseline and every 12 weeks relative to date of enrolment, until objective radiological disease progression as determined by the investigator. Patients could continue to receive olaparib for as long as determined by the investigator, until objective radiological disease progression or as long as in the investigator’s opinion they are benefiting from treatment in relation to other clinical assessments and they do not meet any other discontinuation criteria.

Once a patient has discontinued olaparib she will be managed as per local clinical practice but will remain in the study and data will be collected on subsequent treatments, progression and survival.

# Study objectives

## Primary objective

| **Primary Objective:** | **Outcome Measure:** |
| --- | --- |
| To assess the efficacy of olaparib maintenance monotherapy by investigator assessed progression free survival (PFS) according to RECIST 1.1 criteria. | Time from first dosing date of olaparib to date of disease progression or death from any cause (if this occurs before disease progression) |

## Secondary objectives

| **Secondary Objective:** | **Outcome Measure:** |
| --- | --- |
| To assess the efficacy of olaparib maintenance monotherapy of *BRCA*m population by investigator assessed progression free survival (PFS) according to RECIST 1.1 criteria. | Time from first dosing date of olaparib to date of disease progression or death from any cause (if this occurs before disease progression) in BRCAm population |
| To assess the clinical efficacy of olaparib maintenance monotherapy by assessment of:  a) overall survival (OS),  b) time to investigator- assessed second progression (PFS2), or death, in patients with ovarian cancer. | a) Time from first dosing date of olaparib to date of death from any cause  b) Time from first dosing date of olaparib to date of second progression event or death from any cause (if this occurs before second progression event) |
| To assess the clinical efficacy of olaparib maintenance monotherapy by assessment of  a) time to first subsequent therapy or death (TFST),  b) time to second subsequent therapy or death (TSST) and  c) time to olaparib discontinuation or death (TDT) in patients with ovarian cancer. | a) Time from first dosing date of olaparib to date of first subsequent treatment commencement or death from any cause (if this occurs before commencement of first subsequent treatment)  b) Time from first dosing date of olaparib to date of second subsequent treatment commencement or death from any cause (if this occurs before commencement of second subsequent treatment)  c) Time from first dosing date of olaparib to date of olaparib discontinuation or death from any cause (if this occurs before discontinuation of olaparib maintenance therapy) |

## Safety objectives

| Safety Objective: | Outcome Measure: |
| --- | --- |
| To evaluate the safety and tolerability of olaparib. | AEs/SAEs  Collection of clinical chemistry/haematology parameters |

## Exploratory objectives

| **Exploratory Objective:** | **Outcome Measure :** |
| --- | --- |
| To explore biomarkers in tumor tissues or blood predictive of sensitivity/resistance to the treatment of olaparib | a) Immune-markers PD-L1 expression in tumor tissues  b) *BRCA* and HRR mutation status in the cell-free DNA from blood samples  c) HRD status in tumor samples  d）Relation between having a HRD test and patient demographic and clinical characteristics |

# PATIENT SELECTION, enrolment, restrictions, discontinuation and WITHDRAWAL

Each patient should meet all of the inclusion and none of the exclusion criteria for this study. Under no circumstances can there be exceptions to this rule.

## Inclusion criteria

For inclusion in the study patients should fulfil the following criteria:

1. Provision of informed consent prior to any study specific procedures
2. Age 18 years or over
3. Patients with platinum sensitive relapsed high grade (serous or endometrioid) epithelial ovarian cancers (including primary peritoneal and/or fallopian tube cancer)

- Platinum sensitive disease is defined as disease progression ≥6 months after completion of their last dose of platinum based chemotherapy

1. Patients should have received at least 2 previous lines of platinum containing therapy prior to enrolment:

- For the last chemotherapy course immediately prior to enrolment on the study, patients must be, in the opinion of the investigator, in response (partial or complete radiological response according to RECIST 1.1 criteria) and no evidence of a rising CA-125, following completion of this chemotherapy course.
- Pre-treatment CA-125 measurements must meet criterion specified below:
- If the first value is within upper limit of normal (ULN) the patient is eligible to be randomised and a second sample is not required
- If the first value is greater than ULN a second assessment must be performed at least 7 days after the 1st. If the second assessment is ≥ 15% more than the first the patient is not eligible

1. Have availability of 10 ml blood for germline *BRCA*m/HRRm testing and tumor sample for somatic *BRCA*m/HRRm testing: paraffin-embedded archived tumor tissue block (preferred) or, if a block is not possible, it would be better to have qualified 15 5-μm unstained sections.
2. Patients must have normal organ and bone marrow function measured within 28 days prior to administration of study treatment as defined below:

- Haemoglobin ≥ 10.0 g/dL with no blood transfusions in the past 28 days
- Absolute neutrophil count (ANC) ≥ 1.5 x 10^9^/L
- Platelet count ≥ 100 x 10^9^/L
- Total bilirubin ≤ 1.5 x institutional upper limit of normal (ULN)
- Aspartate aminotransferase (AST) (Serum Glutamic Oxaloacetic Transaminase (SGOT)) / Alanine aminotransferase (ALT) (Serum Glutamic Pyruvate Transaminase (SGPT)) ≤ 2.5 x institutional upper limit of normal unless liver metastases are present in which case they must be ≤ 5x ULN
- Patients must have creatinine clearance estimated using the Cockcroft-Gault equation of ≥51 mL/min:

Estimated creatinine clearance = (140-age [years]) x weight (kg) (x F)^a^

serum creatinine (mg/dL) x 72

^a^ where F=0.85 for females and F=1 for males.

1. Eastern Cooperative Oncology Group (ECOG) performance status 0-1.
2. Patients must have a life expectancy ≥ 16 weeks.
3. Postmenopausal or evidence of non-childbearing status for women of childbearing potential: negative urine or serum pregnancy test within 28 days of study treatment and confirmed prior to treatment on day 1.

Postmenopausal is defined as:

- Amenorrheic for 1 year or more following cessation of exogenous hormonal treatments
- Luteinizing hormone (LH) and Follicle stimulating hormone (FSH) levels in the post menopausal range for women under 50
- radiation-induced oophorectomy with last menses >1 year ago
- chemotherapy-induced menopause with >1 year interval since last menses
- surgical sterilisation (bilateral oophorectomy or hysterectomy)

1. Patients is willing and able to comply with the protocol for the duration of the study including undergoing treatment and scheduled visits and examinations.

## Exclusion criteria

Patients should not enter the study if any of the following exclusion criteria are fulfilled:

1. Involvement in the planning and/or conduct of the study (applies to both AstraZeneca staff and/or staff at the study site)
2. Previous enrolment in the present study
3. Participation in another clinical study with an investigational product during the most recent chemotherapy course
4. Any previous treatment with PARP inhibitor, including olaparib
5. Other malignancy within the last 5 years except: adequately treated non-melanoma skin cancer, curatively treated in situ cancer of the cervix, ductal carcinoma in situ (DCIS), Stage 1, grade 1 endometrial carcinoma, or other solid tumours including lymphomas (without bone marrow involvement) curatively treated with no evidence of disease for ≥5 years. Patients with a history of localised triple negative breast cancer may be eligible, provided they completed their adjuvant chemotherapy more than three years prior to registration, and that the patient remains free of recurrent or metastatic disease
6. Resting ECG with QTc > 470 msec on 2 or more time points within a 24 hour period or family history of long QT syndrome
7. Patients receiving any systemic chemotherapy or radiotherapy (except for palliative reasons) within 3 weeks prior to study treatment
8. Concomitant use of known strong CYP3A inhibitors (eg. itraconazole, telithromycin, clarithromycin, protease inhibitors boosted with ritonavir or cobicistat, indinavir, saquinavir, nelfinavir, boceprevir, telaprevir) or moderate CYP3A inhibitors (eg. ciprofloxacin, erythromycin, diltiazem, fluconazole, verapamil). The required washout period prior to starting olaparib is 2 weeks.
9. Concomitant use of known strong (eg. phenobarbital, enzalutamide, phenytoin, rifampicin, rifabutin, rifapentine, carbamazepine, nevirapine and St John’s Wort ) or moderate CYP3A inducers (eg. bosentan, efavirenz, modafinil). The required washout period prior to starting olaparib is 5 weeks for enzalutamide or phenobarbital and 3 weeks for other agents.
10. Persistent toxicities (>Common Terminology Criteria for Adverse Event (CTCAE) grade 2) caused by previous cancer therapy, excluding alopecia.
11. Patients with myelodysplastic syndrome/acute myeloid leukaemia or with features suggestive of MDS/AML.
12. Patients with symptomatic uncontrolled brain metastases. A scan to confirm the absence of brain metastases is not required. The patient can receive a stable dose of corticosteroids before and during the study as long as these were started at least 4 weeks prior to treatment. Patients with spinal cord compression unless considered to have received definitive treatment for this and evidence of clinically stable disease for 28 days.
13. Major surgery within 2 weeks of starting study treatment, or patients have not recovered from any effects of any major surgery.
14. Patients considered a poor medical risk due to a serious, uncontrolled medical disorder, non-malignant systemic disease or active, uncontrolled infection. Examples include, but are not limited to, uncontrolled ventricular arrhythmia, recent (within 3 months) myocardial infarction, uncontrolled major seizure disorder, unstable spinal cord compression, superior vena cava syndrome, extensive interstitial bilateral lung disease on High Resolution Computed Tomography (HRCT) scan or any psychiatric disorder that prohibits obtaining informed consent.
15. Patients unable to swallow orally administered medication and patients with gastrointestinal disorders likely to interfere with absorption of the study medication.
16. Breast feeding women.
17. Immunocompromised patients, e.g., patients who are known to be serologically positive for human immunodeficiency virus (HIV).
18. Patients with a known hypersensitivity to olaparib or any of the excipients of the product.
19. Patients with known active hepatitis (i.e. Hepatitis B or C) due to risk of transmitting the infection through blood or other body fluids
20. Previous allogenic bone marrow transplant or double umbilical cord blood transplantation (dUCBT)
21. Whole blood transfusions in the last 120 days prior to entry to the study (packed red blood cells and platelet transfusions are acceptable, for timing refer to inclusion criteria no.6)

Procedures for withdrawal of incorrectly enrolled patients see Section 3.4.

## Patient enrolment

Investigator(s) should keep a record of the patient screening log, of patients who entered pre-study screening.

The Investigator(s) will:

- Obtain signed informed consent from the potential patient before any study specific procedures are performed.
- Assign potential patient a unique enrolment number, beginning with ‘E#’.
- Determine patient eligibility. See Section 3.

If a patient withdraws from participation in the study, then her enrolment number cannot be reused.

## Procedures for handling incorrectly enrolled patients

Where a patient does not meet all the eligibility criteria but is incorrectly enrolled and started on treatment, the Investigator should inform the AstraZeneca study physician immediately, and a discussion should occur between the AstraZeneca study team medical advisor and the investigator regarding whether to continue or discontinue the patient from treatment.  The AstraZeneca study physician must ensure all decisions are appropriately documented.

## Methods for assigning treatment groups

Not applicable.

## Methods for ensuring blinding

Not applicable.

## Methods for unblinding

Not applicable.

## Restrictions

Patients of child bearing potential and their partners, who are sexually active, must agree to the use of two highly effective forms of contraception during the period of taking olaparib and for 1 month after last dose of study drug.

### Grapefruit juice

It is not recommended to consume grapefruit juice while on olaparib therapy.

### Contraception

Women of childbearing potential and their partners, who are sexually active, must agree to the use of one highly effective forms of contraception and their partners must use a male condom (as described in Appendix C). This should be started from the signing of the informed consent and continue throughout the period of taking study treatment and for at least 6 months after last dose of study drug(s), or they must totally/truly abstain from any form of sexual intercourse (as described in Appendix C).

For details of acceptable methods of contraception refer to Appendix C Acceptable Birth Control Methods.

## Discontinuation of investigational product

Patients may be discontinued from investigational product (IP) in the following situations:

Patient decision. The patient is at any time free to discontinue treatment, without prejudice to further treatment

Adverse Event

Bone marrow findings consistent with myelodysplastic syndrome (MDS)/acute myeloid leukaemia (AML)

Severe non-compliance with the study protocol

Disease progression (unless in the investigator’s opinion the patient is benefiting from the treatment and does not meet any other discontinuation criteria)

### Procedures for discontinuation of a patient from investigational product

At any time, patients are free to discontinue investigational product or withdraw from the study, without prejudice to further treatment. A patient that decides to discontinue IP will always be asked about the reason(s) and the presence of any adverse events. Adverse events will be followed up and all unused study drug should be returned by the patient.

By discontinuing from IP, the patient is not withdrawn from the study unless the patient withdraws consent to participation in the study. Patients should be followed for progression (if discontinuation in the absence of progression), PFS2 and OS following treatment discontinuation as per the protocol schedule.

Any patient discontinuing investigational product should be seen at 30 days post discontinuation for the evaluations outlined in the study schedule. The patient’s tumour status should be assessed clinically and, if appropriate, disease progression should be confirmed by radiological assessment. After discontinuation of study medication, the principal Investigator/Sub-Investigator will perform the best possible observation(s), test(s) and evaluation(s) as well as give appropriate medication and all possible measures for the safety of the patient. In addition, they will record on the eCRF the date of discontinuation, the reasons, manifestation and treatment at the time of discontinuation. If patients discontinue study treatment, the AstraZeneca monitor must be informed immediately. Patients will be required to attend the treatment discontinuation visit. The patient should return all study medication.

After discontinuation of the study medication at any point in the study, all ongoing AEs or SAEs must be followed until resolution unless, in the Investigator’s opinion the condition is unlikely to resolve due to the patients underlying disease, or the patient is lost to follow up. All new AEs and SAEs occurring during the 30 calendar days after the last dose of study medication must be reported (if SAEs, they must be reported to AstraZeneca within 24 hours as described in Section [6.4](#_Reporting_of_serious)) and followed to resolution as above. Patients should be seen at least 30 days after discontinuing study medication to collect and / or complete AE information. For guidance on reporting adverse events after the 30 day follow up period see Section 6.3.1.1.Any patient who has not yet shown disease progression at withdrawal from IP should continue to be followed as detailed in Table 2.

All patients should be followed for survival, up to the final analysis.

If a patient is withdrawn from study, see Section 3.10.

## Criteria for withdrawal

Reasons for withdrawal from the study:

Voluntary withdrawal by the patient who is at any time free to discontinue their participation in the study, without prejudice to further treatment.

Incorrectly enrolled patients i.e., the patient does not meet the required inclusion/exclusion criteria for the study.

Patient lost to follow-up.

Death.

Withdrawal of the informed consent.

### Screen failures

Screening failures are patients who do not fulfil the eligibility criteria for the study, and therefore must not be enrolled. These patients should have the reason for study withdrawal recorded as ‘Did not meet Eligibility Criteria’ (i.e., patient does not meet the required inclusion/exclusion criteria), and screening failure/ study withdrawal should be noted. This reason for study withdrawal is only valid for screen failures (not enrolled patients). Patients who were screen failures may be re-enrolled and re-screened if in the opinion of the Investigator, the reason(s) for earlier withdrawal no longer applies. Patients cannot re-enter the study if dosed and subsequently withdrawn from the study.

### Withdrawal of the informed consent

Patients are free to withdraw informed consent from the study at any, without prejudice to further treatment.

A patient who withdraws consent will always be asked about the reason(s) and the presence of any adverse events (AE). The Investigator will follow up AEs outside of the clinical study.

If a patient withdraws from participation in the study, then his/her unique enrolment number cannot be reused. Withdrawn patients will not be replaced.

If a patient withdraws consent, they will be specifically asked if they are withdrawing consent to:

- Withdrawal of further participation in the study including any further follow up (e.g., survival calls)
- withdrawal of consent to the use of their study generated data
- withdrawal to the use of any samples

## Discontinuation of the study

The study may be stopped if, in the judgment of AstraZeneca, trial patients are placed at undue risk because of clinically significant findings that are not considered to be consistent with continuation of the study.

Regardless of the reason for termination, all data available for the patient at the time of discontinuation of follow-up must be recorded in the CRF. All reasons for discontinuation of treatment must be documented.

In terminating the study, the Sponsor will ensure that adequate consideration is given to the protection of the patients’ interests.

# Study plan and timing of procedures

**4.1 Study schedule**

Table 1 Study Schedule Screening

| **Day** | -28 to –1  **(First visit)** |
| --- | --- |
| Informed consent | X |
| Demographics | X |
| Medical and surgical history | X |
| Prior cancer therapies including radiotherapy, response to current chemotherapy regimen | X |
| History of blood transfusions^a^ | X |
| Inclusion/exclusion criteria | X |
| ECOG Performance Status (0-1) | X |
| Physical examination | X |
| Vital signs, body weight, (includes blood pressure [BP] and pulse) | X |
| ECG^b^ | X |
| Haematology / clinical chemistry^c^ | X |
| Urinalysis | X |
| Pregnancy test^d^ | X |
| Tumour Assessment (CT or MRI according to RECIST 1.1 criteria)^e^ | X |
| Blood sample for disease specific marker (CA-125) | X |
| Blood sample for determination of *gBRCA* and other non *BRCA* germline HRR status^fh^ | X |
| Tumour sample for determination of *sBRCA* and other non-*BRCA* HRR gene mutation status^gh^ | X |
| Adverse Events (from time of consent) | X |
| Relevant concomitant medications | X |

^a^ Include history of blood transfusion within previous 120 days from start of study treatment and the reasons e.g. bleeding or myelosuppression.

^b^ ECG should be performed once the patient has been in the supine position for at least 5 minutes.

^c^ Coagulation test should be performed at screening and if clinically indicated. For a list of all required laboratory tests please refer to Section [5.2.1](#_Laboratory_safety_assessments).

^d^ Women of child-bearing potential must have a negative urine or serum pregnancy test within 28 days prior to starting treatment and a confirmatory test before treatment on Day 1. If results are positive, the patient is ineligible/must be discontinued from the study.

^e^ RECIST assessments will be performed using CT or MRI scans of neck, chest, abdomen, pelvis. Any other areas of disease involvement should be additionally imaged based on the signs and symptoms of individual patients. Baseline assessments should be performed no more than 28 days before the start of study treatment, and ideally should be performed as close as possible to the start of study treatment.

^f^ The remaining samples will be used for exploratory analysis: BRCA and HRR mutation status in the cell-

free DNA

^g^ The remaining samples will be used for exploratory analysis:Immune-markers PD-L1

h The remaining samples will be used for exploratory analysis: HRD status

| Table 2 Study Schedule – On Study Treatment and Discontinuation | | | | | | | | |
| --- | --- | --- | --- | --- | --- | --- | --- | --- |
| Visit Number | 2 | 3 | 4 | 5 | 6 | Visit No. 7 onwards  Subsequent on treatment visits every 4 or 12 weeks^a^  Tumour assessment visits every 12 or 24 weeks^a,f^ | Study treatment discontinued | Follow-up 30 days after last dose of IP |
| Day | 1 | 8 | 15 | 22 | 29 | Day 1of next visit period  (Visit 7 equals day 57 then visit 8 equals day 85 etc) |  |  |
| Visit Window |  | *±3d* | *±3d* | *±3d* | *±3d* | *±3d* | *±7d* | *±7d* |
| Enrolment | X |  |  |  |  |  |  |  |
| Physical exam^c^ | X^b^ |  |  |  |  |  |  |  |
| Vital signs, body weight (includes BP, pulse and temperature) ^c^ | X^b^ |  |  |  |  |  |  |  |
| ECOG performance status | X |  |  |  |  | X | X |  |
| ECG^c^ | X^b^ |  |  |  |  |  |  |  |
| Haematology / clinical chemistry^d,k^ | X^b^ | X | X | X | X | X | X | X |
| Urinalysis^c^ | X^b^ |  |  |  |  |  |  |  |
| Pregnancy test ^e^ | X |  |  |  | X | X |  |  |
| Blood sample for disease specific marker (CA-125) ^k^ | X |  |  |  | X | X | X |  |
| Tumour Assessment (CT or MRI according to modified RECIST 1.1)^f^ |  |  |  |  |  | X^f^  [Every 12 weeks (±7d) ^f^] |  |  |
| Adverse Events ^g,k^ | X | X | X | X | X | X | X | X |
| Concomitant medications including blood transfusions | X | X | X | X | X | X | X | X |
| Olaparib dispensed/ returned ^h,k^ | X |  |  |  | X^h^ | X^h^ | X |  |
| Subsequent cancer therapy following discontinuation of study treatment ^i^ |  |  |  |  |  |  |  | X |
| Time to second progression ^j,k^ |  |  |  |  |  | X |  |  |
| Survival ^k^ |  |  |  |  |  | X |  |  |

^a^ Visit to take place on Day 1 of a 4 week (28 day) visit period up to 120 weeks (if not progressed and still on treatment, or on study treatment post progression), then on day 1 of a 12 week visit period relative to date of enrolment.

^b^ If assessed within 7 days before enrolment and meets the stated eligibility criteria (if applicable), it does not need to be repeated on Day 1 of study treatment unless investigator believes that it is likely to have changed significantly.

^c^ To be additionally performed if clinically indicated at any other time.

^d^ Safety blood samples do not need to be repeated on Day 1 of study treatment if assessed at least 3 weeks after the last dose of chemotherapy but within 7 days before starting study treatment, unless the investigator believes that it is likely to have changed significantly. Coagulation test should be performed at screening and if clinically indicated. For a list of all required laboratory tests please refer to Section [5.2.1](#_Laboratory_safety_assessments).

^e^ Pregnancy tests on blood or urine samples will be performed for women of childbearing potential within 28 days prior to the start of study treatment, on Day 1 of the study prior to commencing treatment, at the time points shown in Table 2 during study treatment and at the 30 day follow up visit If results are positive the patient is ineligible/must be discontinued from study treatment immediately.

^f^ Follow-up very 12 weeks after start of treatment until objective disease progression as defined by RECIST 1.1, up to 72 weeks, then every 24 weeks relative to date of enrolment. Any other sites at which new disease is suspected should also be appropriately imaged. If an unscheduled assessment was performed and the patient has not progressed, every attempt should be made to perform the subsequent assessments at their scheduled visits.

^g^ All ongoing adverse events/serious adverse events (AEs/SAEs) and any new AEs/SAEs identified during the 30 calendar days follow up period after last dose of study medication must be followed to resolution.

^h^ Sufficient study treatment should be dispensed for at least each treatment period plus overage.

^i^ All anti-cancer treatments (including, but not limited to, chemotherapy and targeted agents), and the investigators opinion of response to them plus the date of progression, post discontinuation of study treatment need to be recorded.

^j^ Only patients on treatment post progression are required to undergo this procedure.

^K^ Patients on treatment post progression are only required to undergo these procedures.

| Table 3 Study Schedule –Follow up Post Discontinuation of Study Treatment | | |
| --- | --- | --- |
| Visit Number | **Off treatment follow up**  **Study treatment discontinued due to reasons other than disease progression**  **Visits every 12 weeks Follow up for 1st progression Tumour assessment visits every 12**  weeks or 24 weeksa | **Time to second progression (PFS2) and Survival for:**  • Patients who have discontinued study treatment due to disease progression  • Patients who have progressed off treatment  • Patients who were on study treatment post progression and / or after primary (PFS) analysis who subsequently discontinued study treatment  Every 12 weeks post discontinuation of study treatment |
| Visit Window | ±7d | ±7d |
| Blood sample for disease specific marker (CA-125) | X |  |
| Tumour Assessment (CT or MRI according to modified RECIST v1.1) a | X |  |
| Adverse Events | X | X |
| ECOG performance status | X | X |
| Subsequent cancer therapy following discontinuation of study treatment ^b^ | X | X |
| Time to second progression |  | X |
| Survival ^cd^ | X | X |

a RECIST follow-up assessments will be performed every 12 weeks (±1 week), up to 72 weeks, then every 24 weeks (±1 week) relative to date of enrolment. Follow-up assessment will include CT or MRI assessments of abdomen and pelvis for all patients. Follow-up chest CT will be performed (in those patients with thoracic lesions or upper abdomen lymphadenopathy identified at baseline assessment). Any other sites at which new disease is suspected should also be appropriately imaged. Patients must be followed until RECIST disease progression. If an unscheduled assessment was performed and the patient has not progressed, every attempt should be made to perform the subsequent assessments at their scheduled visits. Following disease progression further RECIST assessments will not be performed and assessment of disease will be as per local clinical practice.

b All anti-cancer treatments (including, but not limited to, chemotherapy and targeted agents), and the Investigators opinion of response to them and the date of progression need to be recorded.

c The status of ongoing, withdrawn (from the study) and “lost to follow-up” patients at the time of an overall survival analysis should be obtained by the site personnel by checking the patients notes, hospital records, contacting the patients general practitioner and checking publicly available death registries. In the event that the patient has actively withdrawn consent to the processing of their personal data the vital status of the patient can be obtained by site personnel from publicly available resources where it is possible to do so under applicable local laws.

d In addition to their regular 12 weekly contact, patients will be contacted in the 7 days following a specified date (data cut off date) for each survival analysis.

## Enrolment/screening period

Procedures will be performed according to the Study Plan in Table 1.

Each potential patient will provide written informed consent prior to any study specific procedures and undergo assessments applicable for the visit.

Patients will have to consent to undergo germline and tumor *BRCA* testing and other non *BRCA* HRR gene panel testing as required.

All patients will be asked to provide consent to perform exploratory analysis. This consent is included in the main patient informed consent form. However consent for providing this sample is optional and will not prevent patients from enrolment into the study. If patients provide consent to perform exploratory analysis, PD-L1 expression of tumor samples, *BRCA* and HRR mutation status in the cell-free DNA from blood samples, and HRD status in tumor samples will be tested after last patient enrolment.

## Treatment period

Descriptions of the procedures for this period are included in the Study Plan, Table 2 and 3.

## Follow-up period

Patients should be discontinued from olaparib if any discontinuation criteria are fulfilled (see Section 3.9). The assessments to be carried out at the visit are detailed in the study schedule (Table 2 and 3).

### Follow-up 30 days after last dose of investigational product (IP)

A follow-up visit should be conducted 30 days after the last dose of olaparib. Any serious and/or non-serious AEs ongoing at the time of the Discontinuation Visit or which have occurred during the defined 30-day follow-up period must be followed-up. Appropriate safety evaluations should be repeated and/or additional tests performed at any time when clinically indicated, or at the discretion of the investigator, until resolution, unless, in the investigator’s opinion, the condition is unlikely to resolve due to the patient’s underlying disease. If the patient is lost to follow-up, then this should be noted in the eCRF. The assessments to be carried out at the 30-day follow up visit are detailed in the study schedule (Table 2).

### Survival

Assessments for survival should be made every 12 weeks following disease progression. Survival information may be obtained via telephone contact with the patient, patient’s family or by contact with the patient’s current physician. Survival data will be collected up to the time of the final overall survival (OS) analysis. In addition, patients should be contacted in the week following the data cut-off for the primary PFS and final survival analyses to provide complete survival data.

Patients will be followed up as per Table 2 and 3 to the point of the final analysis. At this point investigators will be notified that no further data collection for the study is required. Monitoring and recording of SAEs will continue as per Section 6.4.

The status of ongoing, withdrawn (from the study) and “lost to follow-up” patients at the time of an overall survival analysis should be obtained by the site personnel by checking the patients notes, hospital records, contacting the patients general practitioner and checking publicly available death registries. In the event that the patient has actively withdrawn consent to the processing of their personal data the vital status of the patient can be obtained by site personnel from publicly available resources where it is possible to do so under applicable local laws.

### Second progression (PFS2)

Patients should be assessed every 12 weeks for a second progression (using the patient’s status at first progression as the reference for assessment of second progression). A patient’s progression status is defined according to local standard clinical practice and may involve any of; objective radiological, CA-125, clinical progression or death. The date of PFS2 assessment and investigator opinion of progression status (progressed or non-progressed) at each assessment will be recorded in the eCRF.

### Patient management post primary analysis

The data cut off for the statistical analysis for the primary objective in the patients enrolled will be at 60% maturity for PFS analysis.

Patients on olaparib at the time of the data cut-off will continue to receive olaparib until they meet any discontinuation criteria as per Section 3.9.

Patients on study treatment will be followed for core safety assessments and disease progression. These patients should be followed according to Table 2 and 3.

All patients (patients still on olaparib and patients withdrawn from olaparib) will be followed for survival and disease progression.

### Patient management post final analysis

The data cut off for the final statistical analysis of the study will be when approximately 60% OS maturity.

At this time point, the clinical study database will close to new data. Patients who are receiving olaparib can either choose to discontinue from the study or where the investigator believes patients are gaining clinical benefit, patients may continue to receive olaparib. All patients will receive follow-up care in accordance with standard local clinical practice.

AstraZeneca will continue to supply olaparib to patients after completion of this study if patients are considered to continue to benefit of Olaparib treatment and no other discontinuation criteria are met.

SAEs will continue to be reported to AstraZeneca Patient Safety Department, for any patients who continue on olaparib until 30 days after olaparib is discontinued, in accordance with Section 6.4. Additionally as stated, any SAE or non-serious adverse event that is ongoing at the end of the study must be followed up to resolution unless the event is considered by the investigator to be unlikely to resolve, or the patient is lost to follow-up. If an investigator learns of any SAEs, including death, at any time after a patient has completed the study, and he/she considers there is a reasonable possibility that the event is causally related to the investigational product, the investigator should notify AstraZeneca, Patient Safety.

Drug accountability should continue to be performed until the patient stops olaparib completely.

# study assessments

The Electronic Data Capture (EDC) system will be used for data collection and query handling. The investigator will ensure that data are recorded on the eCRF as specified in the study protocol and in accordance with the instructions provided.

The investigator ensures the accuracy, completeness, and timeliness of the data recorded and of the provision of answers to data queries according to the Clinical Study Agreement. The investigator will sign the completed eCRF. A copy of the completed eCRF will be archived at the study site.

## Efficacy assessments

### CT and MRI scans tumour assessments (Modified RECIST 1.1)

Following the baseline assessment, subsequent tumour assessments according to modified RECIST 1.1 should be performed every 12 weeks (±1week) for the first 72 weeks and then every 24 weeks (± 1 week) thereafter, relative to the date of enrolment, up to objective disease progression by RECIST.

For those patients with no evidence of disease at baseline, following a clinical complete response to chemotherapy, progression is defined by the detection of new lesions on follow up radiological assessments (modified RECIST 1.1).

The imaging modalities used for RECIST assessment will be CT or MRI scans of the neck, chest, abdomen and pelvis with other regions as clinically indicated for the assessment of disease. Any other sites at which new disease is suspected should also be appropriately imaged. The methods of assessment of tumour burden used at baseline must be used at each subsequent follow-up assessment.

Radiological examinations performed in the conduct of this study should be retained at site as source data.

All treatment decisions will be based on site assessment of scans.

### It is important to follow the assessment schedule as closely as possible. If scans are performed outside of scheduled visit ± 1 week window interval and the patient has not progressed, every attempt should be made to perform the subsequent scans at their scheduled time points. Patients will be evaluated until objective radiological disease progression by modified RECIST 1.1 as per the study schedule (see Table 1 and Table 2), and then followed for second progression and survival, regardless of whether study treatment is discontinued or delayed and/or protocol violations, unless they withdraw consent.

### Tumour Evaluation

RECIST 1.1 criteria will be used to assess patient response to treatment by determining progression free survival (PFS) times according to the RECIST 1.1 guideline (Eisenhauer et al 2009).

The methods of assessment of tumour burden used at baseline CT or MRI scans of neck, chest, abdomen and pelvis must be used at each subsequent follow-up assessment. Any other areas of disease involvement should be additionally imaged based on the signs and symptoms of individual patients.

Following the baseline assessment, efficacy for all patients will be assessed by objective tumour assessments every 12 weeks after enrolment, up to 72 weeks, then every 24 weeks until objective disease progression as defined by RECIST 1.1. Any other sites at which new disease is suspected should also be appropriately imaged.

If a patient discontinues treatment (and/or receives a subsequent cancer therapy) prior to progression then the patient should still continue to be followed until objective disease progression as defined by RECIST 1.1.

Categorisation of objective tumour response assessment will be based on the RECIST 1.1 criteria of response: CR (complete response), PR (partial response), SD (stable disease) and PD (progression of disease). Target lesion (TL) progression will be calculated in comparison to when the tumour burden was at a minimum (i.e. smallest sum of diameters previously recorded on study). In the absence of progression, tumour response (CR, PR, SD) will be calculated in comparison to the baseline tumour measurements obtained before starting treatment.

For patients with non-measurable disease only at baseline, categorisation of objective tumour response assessment will be based on the RECIST 1.1 criteria of response: CR (complete response), PD (progression of disease) and Non CR/Non PD.

If the Investigator is in doubt as to whether progression has occurred, particularly with response to NTL (non-target lesion) or the appearance of a new lesion, it is advisable to continue treatment until the next scheduled assessment or sooner if clinically indicated and reassess the patient’s status. If repeat scans confirm progression, then the date of the initial scan should be declared as the date of progression.

To achieve ‘unequivocal progression’ on the basis of non-target disease, there must be an overall level of substantial worsening in non-target disease such that, even in presence of SD or PR in target disease, the overall tumour burden has increased sufficiently to merit discontinuation of therapy. A modest ‘increase’ in the size of one or more non-target lesions is usually not sufficient to quality for unequivocal progression status.

Following progression, patients should continue to be followed up for survival every 12 or 24 weeks as outlined in the study plan.

It is important to follow the assessment schedule as closely as possible. Please refer to the study plan in Table 1 and Table 2 and 3.

## Safety assessments

### Laboratory safety assessments

Blood samples for determination of clinical chemistry and haematology will be taken at the times indicated in the Study Plan.

Any clinical chemistry, haematology and urinalysis required should be performed at a local laboratory at or near to the investigational site. Sample tubes and sample sizes may vary depending on laboratory method used and routine practice at the site.

The following laboratory variable listed in Table 4 will be measured.

NB. In case a patient shows an AST or ALT ≥3xULN or total bilirubin ≥ 2xULN please refer to Appendix B ‘Actions required in cases of combined increase of Aminotransferase and Total Bilirubin – Hy’s Law’, for further instructions.

Table 4 Laboratory Safety Variables

| Haematology/Haemostasis (whole blood) | Clinical Chemistry (serum or plasma) |
| --- | --- |
| B-Haemoglobin (Hb) | S/P-Creatinine |
| B-Leukocyte count | S/P-Bilirubin, total |
| B-Absolute neutrophil count | S/P-Alkaline phosphatase (ALP) |
| B-Absolute lymphocyte count | S/P-Aspartate transaminase (AST) |
| B-Platelet count | S/P-Alanine transaminase (ALT) |
| B-Mean Cell volume (MCV) | S/P-Albumin  S/P- Calcium |
|  | S/P-Potassium |
| **Urinalysis** (dipstick) | S/P-Sodium |
| U-Hb/Erythrocytes/Blood | S/P-Urea or Blood Urea Nitrogen (BUN) |
| U-Protein/Albumin | S/P-Total Protein |
| U-Glucose |  |
|  |  |

**NB.** In case a patient shows an AST **or** ALT ≥3xULN **or** total bilirubin ≥ 2xULN please refer to Appendix B ‘Actions required in cases of combined increase of Aminotransferase and Total Bilirubin – Hy’s Law’, for further instructions.

#### Coagulation

Activated partial thromboblastin time (APTT)will be performed at screening and if clinically indicated.

International normalised ratio (INR) will be performed at screening and if clinically indicated. Patients taking warfarin may participate in this study; however, it is recommended that INR be monitored carefully at least once per week for the first month, then monthly if the INR is stable.

Each coagulation test result will be recorded in eCRF.

#### Bone marrow or blood cytogenetic samples

Bone marrow or blood cytogenetic samples may be collected for patients with prolonged haematological toxicities as defined in Section 6.8.1

Bone marrow analysis should include an aspirate for cellular morphology, cytogenetic analysis and flow cytometry, and a core biopsy for bone marrow cellularity. If it is not possible to conduct cytogenetic analysis or flow cytometry on the bone marrow aspirate, then attempts should be made to carry out the tests on a blood sample. Full reports must be provided by the investigator for documentation on the Patient Safety database. These data will be recorded in eCRF.

#### Disease specific tumour marker samples (CA-125)

As part of the routine safety blood samples, all patients will supply blood sample for CA-125 (2 mL) for assessment at the beginning of each 28 day period prior to the patient receiving study treatment.

It is important to follow the assessment schedule as closely as possible. If CA-125 assessment is performed outside of scheduled visit ± 1 week window interval, every attempt should be made to assess the CA-125 at the scheduled time points. Patients will be evaluated until objective disease progression as defined by RECIST 1.1 (Table 2 and 3).

Further assessment of CA 125 post progression will be at the discretion of the investigator according to local clinical practice.

### Physical examination

Performed at screening, baseline and as clinically indicated (see Table 1 and Table 2).

### ECG

ECGs are required within 7 days prior to starting study treatment and when clinically indicated.

Twelve-lead ECGs will be obtained after the patient has been rested in a supine position for at least 5 minutes in each case. The Investigator or designated physician will review the paper copies of each of the timed 12-lead ECGs on each of the study days when they are collected.

ECGs will be recorded at 25 mm/sec. All ECGs should be assessed by the investigator as to whether they are clinically significantly abnormal / not clinically significantly abnormal. If there is a clinically significant abnormal finding, the Investigator will record it as an AE on the eCRF. The original ECG traces must be stored in the patient medical record as source data.

### Vital signs

Weight will be assessed at screening and baseline according to the Study Schedule (see Table 1 and Table 2) and as clinically indicated at any other time.

Any changes in vital signs should be recorded as an AE, if applicable. For information on how AEs based on changes in vital signs should be recorded and reported, see Section 6.3.

#### Pulse and blood pressure

Blood pressure and pulse will be assessed at screening and baseline according to the Study Schedule (see Table 1 and Table 2) and as clinically indicated at any other time.

Blood pressure and pulse rate will be measured preferably using a semi automatic BP recording device with an appropriate cuff size after 10 minutes rest.

The date of collection and measurement will be recorded on the appropriate eCRF.

#### Body temperature

Body temperature will be measured in degrees Celsius according to local practice at screening, baseline and as clinically indicated (see Table 1 and Table 2).

The date of collection and measurement will be recorded on the appropriate eCRF.

### Other safety assessments

#### Serum or urine pregnancy test

## Pregnancy tests on blood or urine samples will be performed for women of childbearing potential within 28 days prior to the start of study treatment, on Day 1 of the study prior to commencing treatment, at the time points shown in Table 2 during study treatment and at the 30 day follow up visit Tests will be performed by the hospital’s local laboratory. If results are positive the patient is ineligible/must be discontinued from study treatment immediately.

## Pharmacokinetics

### Collection of samples

Pharmacokinetic samples will not be taken during the study.

### Determination of drug concentration

Not applicable.

### Storage and destruction of pharmacokinetic samples

Not applicable.

## Pharmacodynamics

### Collection of samples

Pharmacodynamic samples will not be taken during the study.

### Storage, re-use and destruction of pharmacodynamic samples

Not applicable.

## Pharmacogenetics

Pharmacogenetic samples will not be taken during the study.

### Collection of pharmacogenetic samples

Not applicable.

### Storage, re-use and destruction of pharmacogenetic samples

Not applicable.

## Biomarker analysis

In this study, patients will be asked to provide whole blood sample and tumour sample as part of the study screening procedures. The consent for this will be obtained prior to retrieving whole blood sample and archival tumour sample. These samples will have been collected anytime since the time of original diagnosis but prior to study entry. The patient’s consent before the use of provided biological samples for any biomarker analyses is mandatory.

Whole blood sample and tumour sample will be collected and may be analysed for exploratory biomarkers to assess correlations with disease activity, effects of study drug, clinical outcomes and toxicity.

### Blood sample for germline *BRCA* and other non-*BRCA* HRR gene panel testing

A blood sample (10 mL) will be utilized for germline analysis of *BRCA* and other non *BRCA* HRR gene mutations.

### Tumour sample for somatic *BRCA* and other non-*BRCA* HRR gene panel testing

An adequately sized (minimum of 5 mm x 5 mm x 5 mm) historical tumour tissue paraffin block from resection from the primary tumour or metastases, or 15 qualified 5 μM sections should be provided for somatic *BRCA* or other non *BRCA* HRR gene testing.

### Exploratory blood samples for circulating tumour DNA analysis

10 mL whole blood will be utilized for exploratory circulating tumour DNA analysis of mutations of *BRCA* and HRR genes,

### Tumour sample for exploratory immunohistochemistry analysis of PD-L1 expression

The remaining tumour samples after s*BRCA* and HRR testing will be utilized for the exploratory immunohistochemistry analysis of PD-L1 expression in ovarian cancer patients.

### Exploratory blood and tumor samples for HRD testing

The remaining DNA samples after germline and somatic BRCA/HRR testing will be used for exploratory HRD status analysis in ovarian cancer patients.

### Withdrawal of Informed Consent for provided biological samples

If a patient withdraws consent to the use of provided biological samples, the samples will be disposed of/destroyed, and the action documented. If samples are already analysed, AstraZeneca is not obliged to destroy the results of this research.

Archival tumour / tumour tissue sample: Optional, the patient may continue in the study.

The PI:

Ensures patients’ withdrawal of informed consent to the use of provided samples is notified immediately to AstraZeneca or designated Contract Research Organisation (CRO)

Ensures that biological samples from that patient, if stored at the study site, are immediately identified, disposed of /destroyed, and the action documented

Ensures the laboratory(ies) holding the samples is/are informed about the withdrawn consent immediately and that samples are disposed of/destroyed, the action documented and the signed document returned to the study site

Ensures that the patient and AstraZeneca are informed about the sample disposal

AstraZeneca ensures the central laboratory holding the samples is/are informed about the withdrawn consent immediately and that samples are disposed of/destroyed and the action documented and returned to the study site.

# safety reporting and medical management

The Principal Investigator is responsible for ensuring that all staff involved in the study are familiar with the content of this section.

## Definition of adverse events

An adverse event is the development of an undesirable medical condition or the deterioration of a pre-existing medical condition following or during exposure to a pharmaceutical product, whether or not considered causally related to the product. An undesirable medical condition can be symptoms (e.g., nausea, chest pain), signs (e.g., tachycardia, enlarged liver) or the abnormal results of an investigation (e.g., laboratory findings, electrocardiogram). In clinical studies, an AE can include an undesirable medical condition occurring at any time, including run-in or washout periods, even if no study treatment has been administered.

The term AE is used to include both serious and non-serious AEs.

### Olaparib adverse events of special interest

Adverse events of special interest (AESI) are events of scientific and medical interest specific to the further understanding of olaparib’s safety profile and require close monitoring and rapid communication by the investigators to AstraZeneca. An AESI may be serious or non-serious. Adverse Events of Special Interest for olaparib are the Important identified Risks of MDS/AML, Important potential risk of new primary malignancy (other than MDS/AML) and potential risk of pneumonitis.

ANY event of MDS/AML, new primary malignancy, or pneumonitis should be reported to AstraZeneca Patient Safety whether it is considered a non-serious AE (e.g. non-melanoma skin cancer) or SAE, and regardless of investigator’s assessment of causality or knowledge of the treatment arm.

A questionnaire will be sent to any investigator reporting an AESI, as an aid to provide further detailed information on the event. During the study, there may be other events identified as AESIs that require the use of a questionnaire to help characterise the event and gain a better understanding regarding the relationship between the event and study treatment.

## Definitions of serious adverse event

A serious adverse event is an AE occurring during any study phase (i.e., run-in, treatment, washout, follow-up), that fulfils one or more of the following criteria:

- Results in death
- Is immediately life-threatening
- Requires in-patient hospitalisation or prolongation of existing hospitalisation
- Results in persistent or significant disability/incapacity or substantial disruption of the ability to conduct normal life functions
- Is a congenital abnormality or birth defect
- Is an important medical event that may jeopardise the patient or may require medical intervention to prevent one of the outcomes listed above.

**Comment to Serious Assessment for Invasive and Malignant Cancers:**

Adverse Events (AEs) for malignant tumours reported during a study should generally be assessed as Serious AEs. If no other serious criteria apply, the ‘Important Medical Event’ criterion should be used. In certain situations, however, medical judgement on an individual event basis should be applied to clarify that the malignant tumour event should be assessed and reported as a Non-Serious AE.

However, the instruction above applies only when the malignant tumour event in question is a new malignant tumour (i.e., it is not the tumour for which entry into the study is a criterion and that is being treated by the IP under study and is not the development of new or progression of existing metastasis to the tumour under study). Malignant tumours that – as part of normal, if rare, progression – undergo transformation (e.g., Richter’s transformation of B cell chronic lymphocytic leukemia into diffuse large B cell lymphoma) should not be considered a new malignant tumour.

## Recording of adverse events

### Time period for collection of adverse events

Adverse Events, including Serious Adverse Events, will be collected from time of signature of informed consent, throughout the treatment period and up to and including the 30-day follow-up period. All ongoing and any new AEs/SAEs identified during the 30 calendar days follow up period after last dose of study medication must be followed to resolution.

#### Adverse events after the 30 day follow up period

For Pharmacovigilance purposes and characterisation, any case of MDS/AML or new primary malignancy occurring after the 30 day follow up period should be reported to AstraZeneca Patient Safety whether it is considered a non-serious AE (e.g. non-melanoma skin cancer) or SAE, and regardless of investigator’s assessment of causality or knowledge of the treatment arm.  Investigators will be asked during the regular follow up for overall survival if the patient has developed MDS/AML or a new primary malignancy and prompted to report any such cases.

At any time after a patient has completed the study, if an Investigator learns of any SAE including sudden death of unknown cause, and he/she considers there is a reasonable possibility that the event is causally related to the investigational product, the investigator should notify AstraZeneca, Patient Safety.

Otherwise, after study treatment completion (i.e. after any scheduled post treatment follow-up period has ended) there is no obligation to actively report information on new AEs or SAEs occurring in former study patients. This includes new AEs/SAEs in patients still being followed up for survival but who have completed the post treatment follow up period (30 days).

### Follow-up of unresolved adverse events

Any SAE or non-serious adverse event that is ongoing at the time of the 30-day follow up, must be followed up to resolution unless the event is considered by the investigator to be unlikely to resolve, or the patient is lost to follow up. AstraZeneca retains the right to request additional information for any patient with ongoing AE(s)/SAE(s) at the end of the study, if judged necessary.

### Variables

The following variables will be collect for each AE:

- AE (verbatim)
- The date when the AE started and stopped
- Maximum CTCAE grade
- Whether the AE is serious or not
- Investigator causality rating against the IP (yes or no)
- Action taken with regard to IP
- Outcome

In addition, the following variables will be collected for SAEs:

- Date AE met criteria for serious AE
- Date Investigator became aware of serious AE
- AE is serious due to (provide all seriousness criteria applicable)
- Date of hospitalisation (if applicable)
- Date of discharge (if applicable)
- Probable cause of death (if applicable)
- Date of death (if applicable)
- Autopsy performed (yes or no; if yes provide report) (if applicable)
- Causality assessment in relation to study procedure(s)
- Causality assessment in relation to Other medication
- Description of AE

Severity of AE

For each episode of an adverse event, all changes to the CTCAE grade attained as well as the highest attained CTC grade should be reported.

It is important to distinguish between serious and severe AEs. Severity is a measure of intensity whereas seriousness is defined by the criteria in Section 6.2. An AE of severe intensity need not necessarily be considered serious. For example, nausea that persists for several hours may be considered severe nausea, but not a SAE unless it meets the criteria shown in Section 6.2. On the other hand, a stroke that results in only a limited degree of disability may be considered a mild stroke but would be a SAE when it satisfies the criteria shown in Section 6.2.

The grading scales found in the National Cancer Institute (NCI) CTCAE version 4.0 will be utilised for all events with an assigned CTCAE grading. For those events without assigned CTCAE grades the recommendation is that the CTCAE criteria that convert mild, moderate and severe events into CTCAE grades should be used.

A copy of the CTCAE version can be downloaded from the Cancer Therapy Evaluation program website (http://ctep.cancer.gov).

### Causality collection

The investigator will assess causal relationship between Investigational Product and each Adverse Event, and answer ‘yes’ or ‘no’ to the question ‘Do you consider that there is a reasonable possibility that the event may have been caused by the investigational product?’

For SAEs causal relationship will also be assessed for other medication and study procedures. Note that for SAEs that could be associated with any study procedure the causal relationship is implied as ‘yes’.

A guide to the interpretation of the causality question is found in Appendix A ‘Additional Safety Information’ to the Clinical Study Protocol.

### Adverse events based on signs and symptoms

All AEs spontaneously reported by the patient or care provider or reported in response to the open question from the study personnel: *‘Have you had any health problems since the previous visit/you were last asked?’*, or revealed by observation will be collected and recorded in the CRF. When collecting AEs, the recording of diagnoses is preferred (when possible) to recording a list of signs and symptoms. However, if a diagnosis is known and there are other signs or symptoms that are not generally part of the diagnosis, the diagnosis and each sign or symptom will be recorded separately.

### Adverse events based on examinations and tests

The results from protocol mandated laboratory tests and vital signs will be summarised in the clinical study report. Deterioration as compared to baseline in protocol-mandated laboratory values, vital signs and ECG abnormalities should therefore only be reported as AEs if one of the following is met:

- Any criterion for an SAE is fulfilled
- Causes study treatment discontinuation
- Causes study treatment interruption
- Causes study treatment dose reduction
- The investigator believes that the abnormality should be reported as an AE

If deterioration in a laboratory value/vital sign is associated with clinical signs and symptoms, the sign or symptom will be reported as an AE and the associated laboratory result/vital sign will be considered as additional information. Wherever possible the reporting Investigator uses the clinical, rather than the laboratory term (e.g., anaemia versus low haemoglobin value). In the absence of clinical signs or symptoms, clinically relevant deteriorations in non-mandated parameters should be reported as AE(s).

Deterioration of a laboratory value, which is unequivocally due to disease progression, should not be reported as an AE/SAE.

Any new or aggravated clinically relevant abnormal medical finding at a physical examination as compared with the baseline assessment will be reported as an AE.

### Hy’s Law

Cases where a patient shows elevations in liver biochemistry may require further evaluation and occurrences of AST or ALT ≥ 3xULN together with total bilirubin ≥ 2xULN may need to be reported as SAEs. Please refer to Appendix B for further instruction on cases of increases in liver biochemistry and evaluation of Hy’s Law.

### Disease progression

Disease progression can be considered as a worsening of a patient’s condition attributable to the disease for which the investigational product is being studied. It may be an increase in the severity of the disease under study (DUS) and/or increases in the signs and symptoms of the cancer. The development of new, or progression of existing metastasis to the primary cancer under study should be considered as disease progression and not an AE (if the progression was ‘as expected’). Events, which are unequivocally due to disease progression, should not be reported as an AE during the study.

### New cancers

The development of a new primary cancer (including skin cancer) should be regarded as an AE and will generally meet at least one of the serious criteria (see Section 6.2). New primary cancers are those that are not the primary reason for the administration of the study treatment and have developed after the inclusion of the patient into the study. They do not include metastases of the original cancer. Symptoms of metastasis or the metastasis itself (if ‘as expected’) should not be reported as an AE/SAE, as they are considered to be disease progression.

### Lack of efficacy

When there is deterioration in the ovarian cancer, for which the study treatment(s) is being used, there may be uncertainty as to whether this is lack of efficacy or an AE. In such cases, unless the Sponsor or the reporting physician considers that the study treatment contributed to the deterioration of the condition, or local regulations state to the contrary, the deterioration should be considered to be a lack of efficacy and not an AE.

### Deaths

All deaths that occur during the study, or within the protocol defined 30 day post study follow up period after the administration of the last dose of study treatment, must be reported as follows:

- Death clearly the result of disease progression should be reported to the study monitor at the next monitoring visit and should be documented in the eCRF but should not be reported as an SAE.
- Where death is not due (or not clearly due) to progression of the disease under study, the AE causing the death must be reported to the study monitor as a SAE within 24 hours (see Section 6.2 for further details). The report should contain a comment regarding the co-involvement of progression of disease, if appropriate, and should assign main and contributory causes of death. This information can be captured in the ‘Death eCRF’.
- Deaths with an unknown cause should always be reported as a SAE. A post mortem maybe helpful in the assessment of the cause of death, and if performed a copy of the post-mortem results should be forwarded to AstraZeneca within the usual timeframes.

## Reporting of serious adverse events

All SAEs have to be reported, whether or not considered causally related to the investigational product, or to the study procedure(s). All SAEs will be recorded in the CRF.

If any SAE occurs in the course of the study, then investigators or other site personnel inform the appropriate AstraZeneca representatives immediately, but **no later than 24 hours** of when he or she becomes aware of it.

The designated AstraZeneca representative works with the investigator to ensure that all the necessary information is provided to the AstraZeneca Patient Safety data entry site **within 1 calendar day** of initial receipt for fatal and life-threatening events and within 5 calendar days of initial receipt of all other SAEs.

For fatal or life-threatening adverse events where important or relevant information is missing, active follow-up is undertaken immediately. Investigators or other site personnel inform designated AstraZeneca representatives of any follow-up information on a previously reported SAE within one calendar day i.e., immediately but **no later than 24 hours** of when he or she becomes aware of it.

Once the investigators or other site personnel indicate an AE is serious in the EDC system, an email alert is sent to the designated AstraZeneca representative.

If the EDC system is not available, then the investigator or other study site personnel reports a SAE to the appropriate designated AstraZeneca representative by telephone.

The designated AstraZeneca representative will advise the investigator/study site personnel how to proceed.

The reference document for definition of expectedness/listedness is the IB.

## Overdose

There is currently no specific treatment in the event of overdose with olaparib and possible symptoms of overdose are not established.

Olaparib must only be used in accordance with the dosing recommendations in this protocol. Any dose or frequency of dosing that exceeds the dosing regimen specified in this protocol should be reported as an overdose.

Adverse reactions associated with overdose should be treated symptomatically and should be managed appropriately.

- An overdose with associated AEs is recorded as the AE diagnosis/symptoms on the relevant AE modules in the CRF and on the Overdose CRF module.
- An overdose without associated symptoms is only reported on the Overdose CRF module.

If an overdose on an AstraZeneca study drug occurs in the course of the study, then the investigator or other site personnel inform appropriate AstraZeneca representatives immediately, or **no later than 24 hours** of when he or she becomes aware of it.

The designated AstraZeneca representative works with the investigator to ensure that all relevant information is provided to the AstraZeneca Patient Safety data entry site.

For overdoses associated with a SAE, the standard reporting timelines apply, see Section 6.4. For other overdoses, reporting must occur within 30 days.

## Pregnancy

All pregnancies and outcomes of pregnancy should be reported to AstraZeneca.

### Maternal exposure

If a patient becomes pregnant during the course of the study olaparib should be discontinued immediately.

The outcomes of any conception occurring from the date of the first dose of study medication until 1 month after the last dose of study medication must be followed up and documented.

Pregnancy itself is not regarded as an adverse event unless there is a suspicion that the investigational product under study may have interfered with the efficacy of a contraceptive medication. Congenital abnormalities/birth defects and spontaneous miscarriages should be reported and handled as SAEs. Elective abortions without complications should not be handled as AEs. The outcome of all pregnancies (spontaneous miscarriage, elective termination, ectopic pregnancy, normal birth or congenital abnormality) should be followed up and documented even if the patient was discontinued from the study.

If any pregnancy occurs during the course of the study, then the Investigator or other site personnel informs the appropriate AstraZeneca representatives within 1day i.e., immediately but **no later than 24 hours** of when he or she becomes aware of it.

The designated AstraZeneca representative works with the Investigator to ensure that all relevant information is provided to the AstraZeneca Patient Safety data entry site within 1 calendar days for SAEs (see Section 6.4) and within 30 days for all other pregnancies.

The same timelines apply when outcome information is available.

The Pregnancy Report module in the CRF is used to report the pregnancy and the paper report form is used to report the outcome of the pregnancy.

### Paternal exposure

Not applicable.

## Medication Error

For the purposes of this clinical study a medication error is an unintended failure or mistake in the treatment process for an AstraZeneca study drug that either causes harm to the patient or has the potential to cause harm to the patient. A medication error is not lack of efficacy of the drug, but rather a human or process related failure while the drug is in control of the study site staff or patient.

Medication error includes situations where an error:

- occurred
- was identified and intercepted before the patient received the drug
- did not occur, but circumstances were recognized that could have led to an error

Examples of events to be reported in clinical studies as medication errors:

- Drug name confusion
- Dispensing error, e.g., medication prepared incorrectly, even if it was not actually given to the patient
- Drug not administered as indicated, for example, wrong route or wrong site of administration
- Drug not taken as indicated, e.g., tablet dissolved in water when it should be taken as a solid tablet
- Drug not stored as instructed, e.g., kept in the fridge when it should be at room temperature
- Wrong patient received the medication (excluding IVRS/IWRS errors)
- Wrong drug administered to patient (excluding IVRS/IWRS errors)

Examples of events that **do not** require reporting as medication errors in clinical studies:

- Errors related to or resulting from IVRS/IWRS, including those which lead to one of the above listed events that would otherwise have been a medication error
- Patient accidentally missed drug dose(s), e.g., forgot to take medication
- Accidental overdose (will be captured as an overdose)
- Patient failed to return unused medication or empty packaging
- Errors related to background and rescue medication, or standard of care medication in open label studies, even if an AZ product

Medication errors are not regarded as AEs but AEs may occur as a consequence of the medication error.

If a medication error occurs in the course of the study, then the Investigator or other site personnel informs the appropriate AstraZeneca representatives within 1 day i.e., immediately but **no later than 24** hours of when he or she becomes aware of it.

The designated AstraZeneca representative works with the Investigator to ensure that all relevant information is completed within 1 calendar days if there is an SAE associated with the medication error (see Section 6.4) and within 30 days for all other medication errors.

## Management of IP related toxicities, dose interruptions and dose reductions

Any toxicity observed during the course of the study could be managed by interruption of the dose of study treatment or dose reductions. Repeat dose interruptions are allowed as required, for a maximum of 4 weeks on each occasion. If the interruption is any longer, the study team must be informed. Study treatment can be dose reduced to 250 mg twice daily as a first step and to 200 mg twice daily as a second step. If the reduced dose of 200 mg twice daily is not tolerable, no further dose reduction is allowed and study treatment should be discontinued.

Once dose is reduced, escalation is not permitted.

### Management of haematological toxicity

#### Management of anaemia

Table 5 Management of anaemia

| Haemoglobin | Action to be taken |
| --- | --- |
| **Hb < 10** *but* **≥ 8 g/dl (CTCAE Grade 2)** | Give appropriate supportive treatment and investigate causality.  Investigator judgement to continue olaparib with supportive treatment (eg transfusion) *or* interrupt dose for a maximum of 4 weeks.  If repeat Hb**< 10** *but* **≥ 8 g/dl**, dose interrupt (for max of 4 weeks) until Hb ≥ 10 g/dl and upon recovery dose reduction to **250 mg twice daily** as a first step and to **200 mg twice daily** as a second step may be considered. |
| **Hb < 8 g/dl**  **(CTCAE Grade 3)** | Give appropriate supportive treatment (e.g. transfusion) and investigate causality.  Interrupt olaparib for a maximum of 4 weeks. until improved to Hb ≥ 10 g/dl.  Upon recovery dose reduce to **250 mg twice daily** as a first step and to **200 mg twice daily** as a second step in the case of repeat Hb decrease. |

Common treatable causes of anaemia (e.g., iron, vitamin B12 or folate deficiencies and hypothyroidism) should be investigated and appropriately managed. In some cases management of anaemia may require blood transfusions. For cases where patients develop prolonged haematological toxicity (≥2 week interruption/delay in study treatment due to CTC grade 3 or worse anaemia and/or development of blood transfusion dependence), refer to Section 6.8.1.3 for the management of this.

#### Management of neutropenia, leukopenia and thrombocytopenia

Table 6 Management of neutropenia, leukopenia and thrombocytopenia

| Toxicity | Study treatment dose adjustment |
| --- | --- |
| CTCAE Grade 1-2 | Investigator judgement to continue treatment or if dose interruption, this should be for a maximum of 4 weeks; appropriate supportive treatment and causality investigation |
| CTCAE Grade 3-4 | Dose interruption until recovered to CTCAE gr 1 or better for a maximum of 4 weeks. If repeat CTCAE grade 3-4 occurrence, dose reduce to **250 mg twice daily** as a first step and **200 mg** **twice daily** as a second step |

Adverse event of neutropenia and leukopenia should be managed as deemed appropriate by the investigator with close follow up and interruption of study drug if CTC grade 3 or worse neutropenia occurs.

Primary prophylaxis with Granulocyte colony-stimulating factor (G-CSF) is not recommended, however, if a patient develops febrile neutropenia, study treatment should be stopped and appropriate management including G-CSF should be given according to local hospital guidelines. Please note that G-CSF should not be used within at least 24 h (7 days for pegylated G-CSF) of the last dose of study treatment unless absolutely necessary.

Platelet transfusions, if indicated, should be done according to local hospital guidelines.

For cases where patients develop prolonged haematological toxicity (≥2 weeks interruption/delay in study treatment due to CTC grade 3 or worse), refer to Section 6.8.1.3.

#### Management of of prolonged haematological toxicities while on study treatment

If a patient develops prolonged haematological toxicity such as:

≥2 weeks interruption/delay in study treatment due to CTC grade 3 or worse anaemia and/or development of blood transfusion dependence

≥2 weeks interruption/delay in study treatment due to CTC grade 3 or worse neutropenia (ANC < 1 x 10^9^/L)

≥2 weeks interruption/delay in study treatment due to CTC grade 3 or worse thrombocytopenia and/or development of platelet transfusion dependence (Platelets < 50 x 10^9^/L)

Check weekly differential blood counts including reticulocytes and peripheral blood smear. If any blood parameters remain clinically abnormal after 4 weeks of dose interruption, the patient should be referred to haematologist for further investigations. Bone marrow analysis and/or blood cytogenetic analysis should be considered at this stage according to standard haematological practice. Study treatment should be discontinued if blood counts do not recover to CTC gr 1 or better within 4 weeks of dose interruption.

Development of a confirmed myelodysplastic syndrome or other clonal blood disorder should be reported as an SAE and full reports must be provided by the investigator to AstraZeneca Patient Safety. Olaparib treatment should be discontinued if patient’s diagnosis of MDS and/or AML is confirmed.

### Management of non-haematological toxicity

Repeat dose interruptions are allowed as required, for a maximum of 4 weeks on each occasion. If the interruption is any longer than this the study monitor must be informed. Where toxicity reoccurs following re-challenge with study treatment, and where further dose interruptions are considered inadequate for management of toxicity, then the patient should be considered for dose reduction or must permanently discontinue study treatment.

Study treatment can be dose reduced to 250 mg bd as a first step and to 200 mg bd as a second step. Treatment must be interrupted if any NCI-CTCAE grade 3 or 4 adverse event occurs which the investigator considers to be related to administration of study treatment.

#### Management of new or worsening pulmonary symptoms

If new or worsening pulmonary symptoms (e.g., dyspnoea) or radiological abnormalities occur in the absence of a clear diagnosis, an interruption in study treatment dosing is recommended and further diagnostic workup (including a high resolution CT scan) should be performed to exclude pneumonitis.

Following investigation, if no evidence of abnormality is observed on CT imaging and symptoms resolve, then study treatment can be restarted, if deemed appropriate by the investigator. If significant pulmonary abnormalities are identified, these need to be discussed with the Study Physician.

#### Management of nausea and vomiting

Events of nausea and vomiting are known to be associated with olaparib treatment. In study D0810C00019 nausea was reported in 71% of the olaparib treated patients and 36% in the placebo treated patients and vomiting was reported in 34% of the olaparib treated patients and 14% in the placebo treated patients. These events are generally mild to moderate (CTCAE grade 1 or 2) severity, intermittent and manageable on continued treatment. The first onset generally occurs in the first month of treatment for nausea and within the first 6 months of treatment for vomiting. For nausea, the incidence generally plateaus at around 9 months, and for vomiting at around 6 to 7 months.

No routine prophylactic anti-emetic treatment is required at the start of study treatment, however, patients should receive appropriate anti-emetic treatment at the first onset of nausea or vomiting and as required thereafter, in accordance with local treatment practice guidelines. Alternatively, olaparib tablets can be taken with a light meal/snack (ie 2 pieces of toast or a couple of biscuits).

As per international guidance on anti-emetic use in cancer patients (ESMO, NCCN), generally a single agent antiemetic should be considered eg dopamine receptor antagonist, antihistamines or dexamethasone.

#### Interruptions for intercurrent non-toxicity related events

Study treatment dose interruption for conditions other than toxicity resolution should be kept as short as possible. If a patient cannot restart study treatment within 4 weeks for resolution of intercurrent conditions not related to disease progression or toxicity, the case should be discussed with AZ study physician.

All dose reductions and interruptions (including any missed doses), and the reasons for the reductions/interruptions are to be recorded in the eCRF.

Study treatment should be stopped at least 3 days prior to planned surgery. After surgery study treatment can be restarted when the wound has healed. No stoppage of study treatment is required for any needle biopsy procedure.

Study treatment should be discontinued for a minimum of 3 days before a patient undergoes radiation treatment. Study treatment should be restarted within 4 weeks as long as any bone marrow toxicity has recovered.

Because the AEs related to olaparib may include asthenia, fatigue and dizziness, patients should be advised to use caution while driving or using machinery if these symptoms occur.

| Table 7 Dose reductions for study treatment | | |
| --- | --- | --- |
| Initial Dose | Following re-challenge post interruption: Dose reduction 1 | Dose reduction 2 |
| 300mg twice daily | 250mg twice daily | 200mg twice daily |

## Study governance and oversight

Not applicable.

### Steering Committee

Not applicable.

### Data Monitoring Committee

Not applicable.

### Scientific Advisory Committee

A scientific advisory committee will be established as part of the study to provide guidance on study conduct and interpretation, chaired by the PI and including key investigators and optional external experts as needed. This committee will be facilitated by AstraZeneca.

# investigational product and other Treatments

## Identity of investigational product(s)

AstraZeneca’s Pharmaceutical Development, R&D Supply Chain will supply olaparib to the Investigator as green film-coated tablets.

| Investigational product | Dosage form and strength |  |
| --- | --- | --- |
| Olaparib | Tablet – 150 mg and 100 mg |  |

a Descriptive information for olaparib can be found in the Investigator’s Brochure

## Dose and treatment regimens

Olaparib tablets will be packed in high-density polyethylene (HDPE) bottles with child-resistant closures. Each dosing container will contain sufficient medication for at least 28 days plus overage. Olaparib will be dispensed to patients on Day 1 and every 28 days thereafter until the patient completes the study, withdraws from the study or closure of the study.

Study treatment is available as a green film-coated tablet containing 150 mg or 100 mg of olaparib.

Patients will be administered olaparib tablets orally at a dose of 300 mg twice daily. Doses of study treatment should be taken at the same times each day approximately 12 hours apart with one glass of water. The study treatment tablets should be swallowed whole and not chewed, crushed, dissolved or divided. Olaparib tablets can be taken with or without food.

If vomiting occurs shortly after the olaparib tablets are swallowed, the dose should only be replaced if all of the intact tablets can be seen and counted. Should any patient enrolled on the study miss a scheduled dose for whatever reason (e.g. as a result of forgetting to take the tablets or vomiting), the patient will be allowed to take the scheduled dose up to a maximum of 2 hours after that scheduled dose time. If greater than 2 hours after the scheduled dose time, the missed dose is not to be taken and the patient should take their allotted dose at the next scheduled time.

There is no maximum duration for taking olaparib. Patients should continue to receive olaparib until disease progression as assessed by the investigator or as long as in the investigator’s opinion they are benefiting from treatment and they do not meet any other discontinuation criteria as outlined in Section 3.9.

**Dose Reductions**

For guidance on dose reductions for management of AEs refer to section [6.8](#_Management_of_IP).

For guidance on dose reductions when concomitant strong or moderate CYP3A inhibitors cannot be avoided see section [7.7](#_Concomitant_and_other).

**Renal Impairment**

If subsequent to study entry and while still on study therapy, a patient’s estimated CrCl

falls below the threshold for study inclusion (≥51 ml/min), retesting should be performed promptly.

A dose reduction is recommended for patients who develop moderate renal impairment (calculated creatinine clearance by Cockcroft-Gault equation of between 31 and 50 ml/min) for any reason during the course of the study: the dose of olaparib should be reduced to 200mg BD.

Because the CrCl determination is only an estimate of renal function, in instances where the CrCl falls to between 31 and 50 mL/min, the investigator should use his or her discretion in determining whether a dose change or discontinuation of therapy is warranted.

Olaparib has not been studied in patients with severe renal impairment (creatinine clearance ≤ 30 ml/min) or end-stage renal disease; if patients develop severe impairment or end stage disease is it recommended that olaparib be discontinued.

## Labelling

Labels will be prepared in accordance with Good Manufacturing Practice (GMP) and local regulatory guidelines. The labels will fulfil GMP Annex 13 requirements for labelling. Label text will be translated into local language.

Specific dosing instructions will not be included on the label, the site must complete the “Patient Dispensing Card” with the details of the dosing instructions at the time of dispensing.

The patient emergency contact details will not be on the label, but can be found in the informed consent and the ‘Patient Dispensing Card’. For emergency purposes the patient must be in possession of the emergency contact details at all times.

## Storage

All study drugs should be kept in a secure place under appropriate storage conditions. The investigational product label on the bottle specifies the appropriate storage.

## Compliance

The administration of investigational product should be recorded in the appropriate sections of the Case Report Form.

Patients should be given clear instructions on how and when to take olaparib. Patients will self-administer olaparib. Study site staff will make tablet counts at regular intervals during treatment. Compliance will be assessed by the tablet count and the information will be recorded in the appropriate section of the eCRF. After the tablet count has been performed, the remaining tablets will not be returned to the patient but will be retained by the investigative site until reconciliation is completed by the study monitor. All patients must return their bottle(s) of olaparib at the appropriate scheduled visit, when a new bottle will be dispensed. Patients will be instructed to notify study site personnel of missed (forgotten) doses. Dates of missed or held doses will be recorded by the patient on their patient diary and by the site staff on the eCRF.

Patients must return all containers and any remaining IP tablets at the end of the study.

## Accountability

The IP provided for this study will be used only as directed in the study protocol.

The study personnel will account for all IP dispensed to and returned from the patient.

Study site personnel or the study monitor will account for IP received at the site, unused study drugs and for appropriate destruction. Certificates of delivery, destruction or return should be signed.

## Concomitant and other treatments

The use of any natural/herbal products or other traditional remedies should be discouraged, but use of these products, as well as use of all vitamins, nutritional supplements, and all other concomitant medications must be recorded in the case report form (CRF).

Medications that may NOT be administered

No other anti-cancer therapy (chemotherapy, immunotherapy, hormonal therapy (Hormone replacement therapy (HRT) is acceptable), radiotherapy, biological therapy or other novel agent) is to be permitted while the patient is receiving study medication.

Live virus and live bacterial vaccines should not be administered whilst the patient is receiving study medication and during the 30 day follow up period. An increased risk of infection by the administration of live virus and bacterial vaccines has been observed with conventional chemotherapy drugs and the effects with olaparib are unknown.

Restricted concomitant medications

*Strong or Moderate CYP3A inhibitors*

Known strong CYP3A inhibitors (e.g., itraconazole, telithromycin, clarithromycin, boosted protease inhibitors, indinavir, saquinavir, nelfinavir, boceprevir, telaprevir) or moderate CYP3A inhibitors (ciprofloxacin, erythromycin, diltiazem, fluconazole, verapamil) should not be taken with olaparib.

If there is no suitable alternative concomitant medication then the dose of olaparib should be reduced for the period of concomitant administration. The dose reduction of olaparib should be recorded in the CRF with the reason documented as concomitant CYP3A inhibitor use.

- Strong CYP3A inhibitors – reduce the dose of olaparib to 100mg bd for the duration of concomitant therapy with the strong inhibitor and for 5 half lives afterwards.
- Moderate CYP3A inhibitors - reduce the dose of olaparib to 150mg bd for the duration of concomitant therapy with the moderate inhibitor and for 3 half lives afterwards.
- After the washout of the inhibitor is complete, the olaparib dose can be re-escalated.

*Strong or Moderate CYP3A inducers*

Strong (e.g., phenobarbital, phenytoin, rifampicin, rifabutin, rifapentine, carbamazepine, nevirapine, enzalutamide and St John’s Wort) and moderate CYP3A inducers (eg. bosentan, efavirenz, modafinil) of CYP3A should not be taken with olaparib.

If the use of any strong or moderate CYP3A inducers are considered necessary for the patient’s safety and welfare this could diminish the clinical efficacy of olaparib.

If a patient requires use of a strong or moderate CYP3A inducer then they must be monitored carefully for any change in efficacy of olaparib.

*P-gp inhibitors*

It is possible that co-administration of P-gp inhibitors (eg amiodarone, azithromycin) may increase exposure to olaparib. Caution should therefore be observed.

*Effect of olaparib on other drugs*

Based on limited *in vitro* data, olaparib may increase the exposure to substrates of CYP3A4, P-gp, OATP1B1, OCT1, OCT2, OAT3, MATE1 and MATE2K.

Based on limited *in vitro* data, olaparib may reduce the exposure to substrates of CYP3A4, 2B6, 2C9, 2C19 and P-gp.

The efficacy of hormonal contraceptives may be reduced if co administered with olaparib.

Caution should therefore be observed if substrates of these isoenzymes or transporter proteins are co-administered.

Examples of substrates include:

- CYP3A4 – hormonal contraceptive, simvastatin, cisapride, cyclosporine, ergot alkaloids, fentanyl, pimozide, sirolimus, tacrolimus and quetiapine
- CYP2B6 – bupropion, efavirenz
- CYP2C9 – warfarin
- CYP2C19 - lansoprazole, omeprazole, S-mephenytoin
- P-gp - simvastatin, pravastatin, digoxin, dabigatran, colchicine
- OATP1B1 - bosentan, glibenclamide, repaglinide, statins and valsartan
- OCT1, MATE1, MATE2K – metformin
- OCT2 - serum creatinine
- OAT3 -furosemide, methotrexate

Anticoagulant Therapy

Patients who are taking warfarin may participate in this trial; however, it is recommended that international normalised ratio (INR) be monitored carefully at least once per week for the first month, then monthly if the INR is stable. Subcutaneous heparin and low molecular weight heparin are permitted.

Anti-emetics/Anti-diarrhoeals

From screening part 2 onwards, should a patient develop nausea, vomiting and / or diarrhoea, then these symptoms should be reported as AEs (see section 6.3) and appropriate treatment of the event given.

Palliative radiotherapy

Palliative radiotherapy may be used for the treatment of pain at the site of bony metastases that were present at baseline, provided the investigator does not feel that these are indicative of clinical disease progression during the study period. Study treatment should be discontinued for a minimum of 3 days before a patient undergoes therapeutic palliative radiation treatment. Study treatment should be restarted within 4 weeks as long as any bone marrow toxicity has recovered.

Administration of other anti-cancer agents

Patients must not receive any other concurrent anti-cancer therapy, including investigational agents, while on study treatment. Patients may continue the use of bisphosphonates or denosumab for bone disease and corticosteroids for the symptomatic control of brain metastases provided the dose is stable before and during the study and they were started at least 4 weeks prior to beginning study treatment.

Subsequent therapies for cancer

Details of first and subsequent therapies for cancer and/or details of surgery for the treatment of the cancer, after discontinuation of treatment, will be collected. Reasons for starting subsequent anti-cancer therapies including access to other PARP inhibitors or investigational drugs will be collected and included in the exploratory assessments of OS.

### Other concomitant treatment

Other medication other than that described above, which is considered necessary for the patient’s safety and well being, may be given at the discretion of the Investigator and recorded in the appropriate sections of the Case Report Form.

In addition, any unplanned diagnostic, therapeutic or surgical procedure performed during the study period must be recorded in the eCRF.

## Post Study Access to Olaparib

After the end of the study, patients still on study therapy at that time will continue to receive olaparib outside the study until they continue to derive clinical benefit from treatment in the view of the investigator and no other criteria for treatment discontinuation are met.

# Statistical ANALYSES BY ASTRAZENECA

## Statistical considerations

Analyses will be performed by AstraZeneca or its representatives.

A comprehensive statistical analysis plan (SAP) will be prepared and any subsequent amendments will be documented, with final amendments completed prior to the data cut for the primary analysis.

## Sample size estimate

The sample size of approximately 220 patients is driven by the need to enrol an adequate number of patients to help understand the efficacy and safety of olaparib with the tablet formulation. Around 46 patients with BRCAm ovarian cancer will be enrolled assuming that BRCA mutation rate is 21%.

The primary analysis will be performed after the accumulation of 132 progression or death events which corresponds to 60% maturity. Assuming a median PFS value of 8.4 months, 132 PFS events will provide a 95% confidence interval of (7.1 m, 10.0 m) for the median PFS. Assuming a median PFS of 19.1 months in the BRCA mutated patients, 27 PFS events (60% of 46 BRCA mutated patients) will provide a 95% confidence interval of (13.1 m, 27.9 m) for the median PFS.

## Definitions of analysis sets

Full analysis set (FAS)

The full analysis set will include all subjects who received at least one dose of study treatment.

All analyses will be performed based on the FAS.

## Outcome measures for analyses

### Calculation or derivation of efficacy variable(s)

At each visit patients will be assigned a visit response of CR, PR, SD, PD, NE based on RECIST criteria or other methods depending on the status of their disease compared to baseline and previous assessments, based on the investigator assessment.

### Primary endpoint

PFS is defined as the time from first dose of olaparib until the date of investigator assessed disease progression according to RECIST 1.1 criteria or death (by any cause in the absence of disease progression) regardless of whether the patient withdraws from therapy or receives another anticancer therapy prior to disease progression. Patients who have not progressed or died at the time of analysis will be censored at the time of the latest date of assessment from their last evaluable efficacy assessment. However, if the patient progresses or dies after two or more missed visits, the patient will be censored at the time of the latest evaluable assessment. Given the scheduled visit assessment scheme, two missing visits will equate to more than 26 weeks since the previous RECIST assessment, allowing for early and late visits. If the patient has no evaluable visits or does not have a baseline assessment they will be censored at day 1 unless they die within two visits of baseline (25 weeks allowing for visit window).

The PFS time will always be derived based on scan/assessment dates not visit dates. Assessments/scans contributing towards a particular visit may be performed on different dates. The following rules will be applied:

Date of progression will be determined based on the earliest of the assessment/scan dates of the component that triggered the progression.

When censoring a patient for PFS the patient will be censored at the latest of the assessment/scan dates contributing to a particular overall visit assessment.

Overall visit assessments will be determined for each assessment (scheduled or unscheduled) and will contribute to the derivation of PFS.

Disease progression is defined as at least a 20% increase in the sum of the diameters of the target lesions (compared to previous minimum sum) and an absolute increase of > 5 mm, or an overall non-target lesion assessment of progression or a new lesion.

### Secondary endpoints

**Overall Survival**

Overall survival is defined as the time from the date of first dose of olaparib until the date of death due to any cause. Any patient not known to have died at the time of analysis will be censored based on the last recorded date on which the patient was known to be alive.

**Time from first dose to second progression (PFS2)**

Time to second progression is defined as the time from the date of first dose of olaparib to the date of the earliest progression event subsequent to that used for the primary variable PFS or death from any cause. The date of second progression will be recorded by the investigator and defined according to local standard clinical practice and may involve any of objective radiological, symptomatic, CA-125 progression or death. Second progression status will be reviewed every 12 weeks following the progression event used for the primary variable PFS (the first progression) and status recorded. Patients alive and for whom a second disease progression has not been observed should be censored at the last time known to be alive and without a second disease progression, i.e. censored at the last assessment for progression date if the patient has not had a second progression or death.

**Time to first subsequent treatment or death (TFST)**

As a supportive summary to PFS, time to start of first subsequent treatment or death will be assessed. Time to first subsequent treatment or death is defined as the time from the date of first dose of olaparib to the first subsequent treatment start date, or death date. Any patient not known to have had a further subsequent therapy or death will be censored at the last known time to have not received subsequent treatment.

**Time to study treatment discontinuation or death (TDT)**

Time to study treatment discontinuation or death (TDT) will be assessed. TDT is defined as the time from the date of first dose of olaparib to the date of study treatment discontinuation or death from any cause. Any patient not known to have died at the time of analysis and not known to have discontinued study treatment will be censored based on the last recorded date on which the patient was known to be alive.

**Time to second subsequent treatment or death (TSST)**

As a supportive summary to PFS2, time to start of second subsequent treatment or death will be assessed. Time to second subsequent treatment or death is defined as the time from the date of first dose of olaparib to the earlier of the date of second subsequent treatment start date, or death date. Any patient not known to have had a further second subsequent therapy or death will be censored at the last known time to have not received second subsequent treatment.

### Calculation or derivation of safety variable(s)

Safety and tolerability will be assessed in terms of AEs, deaths, laboratory data, and vital signs. These will be collected for all patients. The number of patients experiencing each AE (based on MedDRA preferred term) will be summarized by CTCAE grade.

### Other significant adverse events (OAE)

During the evaluation of the AE data, an AstraZeneca medically qualified expert will review the list of AEs that were not reported as SAEs and discontinuation of investigational product due to adverse events (DAEs). Based on the expert’s judgement, significant adverse events of particular clinical importance may, after consultation with the Global Patient Safety Physician, be considered OAEs and reported as such in the Clinical Study Report. A similar review of laboratory/vital signs will be performed for identification of OAEs. Examples of these are marked haematological and other laboratory abnormalities, and certain events that lead to intervention (other than those already classified as serious), dose reduction or significant additional treatment.

## Methods for statistical analyses

Data will be analyzed on the FAS population. The primary analysis will be performed at the time of accrual of 132 progression or death events in the 220 enrolled patients. The final analysis will be performed when approximately 60% OS maturity is achieved.

Data will be summarized using descriptive statistics as appropriate. Continuous variables will be summarised by the number of observations (n), mean, standard deviation (SD), median, quartiles (Q1 and Q3), minimum, and maximum. Categorical variables will be summarised by frequency counts and percentages for each category.

### Analysis of the primary variable (s)

PFS will be analysed when approximately 132 progression events have occurred in the study (60% maturity). This analysis will address the primary objective of the trial. The updated results will also be provided at the final analysis.

A Kaplan-Meier (KM) plot of PFS will be presented. Summaries of the number and percentage of patients experiencing a PFS event, and the type of event (progression or death) will be provided along with median PFS and 95% confidence interval.

The analysis on PFS will be based on RECIST 1.1 criteria assessed by investigators, and using all scans regardless of whether they were scheduled or not. The estimated PFS rates at 6 months, 12 months and 24 months will be summarised (using the KM curve) for all patients.

The number of patients prematurely censored will be summarised together with baseline prognostic factors of the prematurely censored patients. A patient is defined as prematurely censored if they had not progressed and the latest scan prior to the date of cut off (DCO) was more than one scheduled tumour assessment interval (+ 2 weeks) prior to the DCO date.

### Analysis of the secondary variable(s)

All time to event endpoints will be described as for PFS.

Safety data will be summarised descriptively.

### Subgroup analysis (if applicable)

Subgroup analyses will be performed, as described for the primary analysis of PFS and secondary analysis of OS, to assess the consistency of olaparib effect across potential or expected prognostic factors.

The following subgroups of the PFS and OS may be summarised.

- Response to previous platinum chemotherapy (CR vs PR)
- Time to disease progression on last platinum based chemotherapy received prior to first dose of olaparib (6 – 12 months / > 12 months)
- Measurable versus non-measurable disease at baseline
- *BRCA* mutation type, e.g. germline, somatic *BRCA*; *BRCA1, BRCA2* or *BRCA1/2* (both)
- *HRR* mutation type
- Age at first dose (<65 vs. ≥65)
- Country
- Prior bevacizumab use

Number of prior chemotherapy lines Other baseline variables may also be assessed if there is clinical justification.

### Interim analysis

Interim analysis for OS is planned approximately 3years after last patients enrolled if 60% OS maturity is not achieved by then.

### Exploratory analysis

Biomarker data will be summarized with descriptive statistics. The relationship between biomarkers and treatment effect of olaparib will be explored. The details will be described in the study SAP or a separate exploratory analysis plan.

Due to the retrospective nature of the HRD testing, and that tissue testing for BRCA status will be carried out first, HRD testing will only be possible in those patients with remaining tumour sample/FFPE sample of sufficient quality and quantity. To ascertain whether systematic differences exist between patients with and without necessary tumour sample (or in effect a HRD test), the relation between having a HRD test and patient demographic and clinical characteristics will be investigated using t-tests or chi-squared tests as appropriate for the variable. Participant characteristics will be summarized separately by HRD test using means (SD) and percentages (number).

# STUDY and data MANAGEMENT by astrazeneca

## Training of study site personnel

Before the first patient is entered into the study, a designated AstraZeneca representative will review and discuss the requirements of the Clinical Study Protocol and related documents with the investigational staff and also train them in any study specific procedures and the EDC and/or any other system(s) utilised.

The PI will ensure that appropriate training relevant to the study is given to all of these staff, and that any new information relevant to the performance of this study is forwarded to the staff involved.

The PI will maintain a record of all individuals involved in the study (medical, nursing and other staff).

## Monitoring of the study

During the study, an AstraZeneca representative will have regular contacts with the study site, including visits to:

Provide information and support to the Investigator(s)

Confirm that facilities remain acceptable

Confirm that the investigational team is adhering to the protocol, that data are being accurately and timely recorded in the CRFs, that biological samples are handled in accordance with the Laboratory Manual and that study drug accountability checks are being performed

Perform source data verification (a comparison of the data in the CRFs with the patient’s medical records at the hospital or practice, and other records relevant to the study) including verification of informed consent of participating patients. This will require direct access to all original records for each patient (e.g., clinic charts)

Ensure withdrawal of informed consent to the use of the patient’s biological samples is reported and biological samples are identified and disposed of/destroyed accordingly, and the action is documented, and reported to the patient.

The AstraZeneca representative will be available between visits if the Investigator(s) or other staff at the centre needs information and advice about the study conduct.

### Source data

Refer to the Clinical Study Agreement for location of source data.

### Study agreements

The PI at each/the centre should comply with all the terms, conditions, and obligations of the Clinical Study Agreement, or equivalent, for this study. In the event of any inconsistency between this Clinical Study Protocol and the Clinical Study Agreement, the terms of Clinical Study Protocol shall prevail with respect to the conduct of the study and the treatment of patients and in all other respects, not relating to study conduct or treatment of patients, the terms of the Clinical Study Agreement shall prevail.

Agreements between AstraZeneca and the PI should be in place before any study-related procedures can take place, or patients are enrolled.

### Archiving of study documents

The Investigator follows the principles outlined in the Clinical Study Agreement (CSA).

### Deviation from the clinical study protocol

The Investigator(s) must not deviate from or make any changes to the protocol without documented agreement between the Principal Investigator and AstraZeneca or the IRB approval based on its deliberations.  However, this shall not apply to cases where the deviation or change is necessary to avoid an immediate hazard to the patients or for other compelling medical reasons, or where the changes involve only logistical or administrative aspects of the clinical study (e.g., changes to the organisation/structure of the AstraZeneca, the name/department name of the study site, the address or phone number of the study site or AstraZeneca, the job title of the Investigator, and monitors).

The Investigator(s) should document any deviation from the protocol regardless of their reasons.  Only when the protocol was not followed in order to avoid an immediate hazard to the patients or for other medically compelling reason, the Investigator should prepare and submit the records explaining the reasons thereof to AstraZeneca and the head of study site, and retain a copy of the records.

The Investigator(s) may deviate from or make a change to the protocol without documented agreement between the Principal Investigator and AstraZeneca or the IRB approval, only in the event of a medical emergency, e.g., it is only way to avoid an immediate hazard to the patients.  In such case, the Principal Investigator must notify details of the deviation or change, the reason, and a proposed revision in the protocol if required, to AstraZeneca and the head of the study site and IRB via the head of the study site as soon as possible, in order to obtain their approval.  A certificate of approval by the head of the study site as well as AstraZeneca should be obtained via the head of the study site.

## Study timetable and end of study

The end of the study is defined as ‘the last visit of the last patient undergoing the study’.

There will be a final data cut-off defined as the time when 60% patients achieved overall survival. At this time point, the clinical study database will close to new data. Patients are, however, permitted to continue to receive study treatment beyond the closure of the database if, in the opinion of the Investigator, they are continuing to receive benefit from treatment with olaparib. For patients who do continue to receive treatment beyond the time of this data cut-off, Investigators will continue to report all SAEs to AstraZeneca Patient Safety until 30 days after study treatment is discontinued, in accordance with Section [6.4](#_Reporting_of_serious) (Reporting of Serious Adverse Events). If an Investigator learns of any SAEs, including death, at any time after a patient has completed the study, and he/she considers there is a reasonable possibility that the event is causally related to the investigational product, the Investigator should notify AstraZeneca, Patient Safety. Additionally as stated in Section 6.3 (Recording of adverse events), any SAE or non-serious adverse event that is ongoing at the time of this data cut-off, must be followed up to resolution unless the event is considered by the investigator to be unlikely to resolve, or the patient is lost to follow-up.

The study is expected to start in Q2 2018 and to end by Q2 2024.

The study may be terminated at individual centres if the study procedures are not being performed according to GCP, or if recruitment is slow. AstraZeneca may also terminate the entire study prematurely if concerns for safety arise within this study or in any other study with olaparib.

## Data management by AstraZeneca

Data management will be performed by an AstraZeneca representative, according to the Data Management Plan (DMP)*.*

The data collected through third party sources will be obtained and reconciled against study data.

Adverse events and medical/surgical history will be classified according to the terminology of the latest version the Medical Dictionary for Regulatory Activities (MedDRA). Medications will be classified according to the AstraZeneca Drug Dictionary. All entries to the study database will be available in an audit trail.

The data will be validated as defined in the DMP. Quality control procedures will be applied to each stage of data handling to ensure that all data are reliable and have been processed correctly. The Plans will also clarify the roles and responsibilities of the various functions and personnel involved in the data management process.

When all data have been coded, validated, and locked, clean file will be declared. Any treatment revealing data may thereafter be added and the final database will be locked.

**Data management of genotype data**

Exploratory genotype data generated in this study will be stored in the AstraZeneca genotyping LIMS database, or other appropriate secure system within AstraZeneca and/or third party contracted to work with AstraZeneca to analyse samples.

Some or all of the clinical datasets from the main study may be merged with the genetic data in a suitable secure environment separate from the clinical database.

**Data associated with human biological samples**

Data associated with biological samples will be transferred from laboratory (ies) internal or external to AstraZeneca.

Management of external data

Data from external providers (e.g. central laboratories) will be validated as appropriate to ensure it is consistent with the clinical data and included in the final database.Tumour tissue or blood for exploratory analyses data, the results of any analyses will not be recorded in the database, but information relating to the processing of the sample, including the original date of biopsy (historical tumour tissue sample and the actual date the sample(s) were collected) will be recorded in the eCRF and database.

# Ethical and regulatory requirements

## Ethical conduct of the study

The study will be performed in accordance with ethical principles that have their origin in the Declaration of Helsinki and are consistent with International Conference on Harmonisation (ICH)/Good Clinical Practice (GCP), applicable regulatory requirements and the AstraZeneca policy on Bioethics and Human Biological Samples.

## Patient data protection

The Informed Consent Form will incorporate (or, in some cases, be accompanied by a separate document incorporating) wording that complies with relevant data protection and privacy legislation.

## Ethics and regulatory review

An Ethics Committee (EC) should approve the final study protocol, including the final version of the Informed Consent Form and any other written information and/or materials to be provided to the patients. The Investigator will ensure the distribution of these documents to the applicable Ethics Committee, and to the study site staff.

The opinion of the Ethics Committee should be given in writing. The Investigator should submit the written approval to AstraZeneca before enrolment of any patient into the study.

The Ethics Committee should approve all advertising used to recruit patients for the study.

AstraZeneca should approve any modifications to the Informed Consent Form that are needed to meet local requirements.

If required by local regulations, the protocol should be re-approved by the Ethics Committee annually.

Before enrolment of any patient into the study, the final study protocol, including the final version of the Informed Consent Form, is approved by the national regulatory authority or a notification to the national regulatory authority is done, according to local regulations.

AstraZeneca will handle the distribution of any of these documents to the national regulatory authorities.

AstraZeneca will provide Regulatory Authorities, Ethics Committees and Principal Investigators with safety updates/reports according to local requirements.

Each PI is responsible for providing the Ethics Committees/IRB with reports of any serious and unexpected adverse drug reactions from any other study conducted with the investigational product. AstraZeneca will provide this information to the PI so that he/she can meet these reporting requirements.

## Informed consent

The Principal Investigator(s) at each centre will:

Ensure each patient is given full and adequate oral and written information about the nature, purpose, possible risk and benefit of the study

Ensure each patient is notified that they are free to discontinue from the study at any time

Ensure that each patient is given the opportunity to ask questions and allowed time to consider the information provided

Ensure each patient provides signed and dated informed consent before conducting any procedure specifically for the study

Ensure the original, signed Informed Consent Form(s) is/are stored in the Investigator’s Study File

Ensure a copy of the signed Informed Consent Form is given to the patient

Ensure that any incentives for patients who participate in the study as well as any provisions for patients harmed as a consequence of study participation are described in the informed consent form that is approved by an Ethics Committee.

## Changes to the protocol and informed consent form

Study procedures will not be changed without the mutual agreement of the International co-ordinating Investigator, the Principal Investigator and AstraZeneca or designated CRO.

If there are any substantial changes to the study protocol, then these changes will be documented in a study protocol amendment and where required in a new version of the study protocol (Revised Clinical Study Protocol).

The amendment is to be approved by the relevant Ethics Committee and if applicable, also the national regulatory authority approval, before implementation. Local requirements are to be followed for revised protocols.

AstraZeneca or designated CRO will distribute any subsequent amendments and new versions of the protocol to each Principal Investigator(s). For distribution to Ethics Committee see Section 10.3.

If a protocol amendment requires a change to a centre’s Informed Consent Form, AstraZeneca and the centre’s Ethics Committee are to approve the revised Informed Consent Form before the revised form is used.

If local regulations require, any administrative change will be communicated to or approved by each Ethics Committee.

## Audits and inspections

Authorised representatives of AstraZeneca, a regulatory authority, or an Ethics Committee may perform audits or inspections at the centre, including source data verification. The purpose of an audit or inspection is to systematically and independently examine all study-related activities and documents, to determine whether these activities were conducted, and data were recorded, analysed, and accurately reported according to the protocol, Good Clinical Practice (GCP), guidelines of the International Conference on Harmonisation (ICH), and any applicable regulatory requirements. The Investigator will contact AstraZeneca immediately if contacted by a regulatory agency about an inspection at the centre.

# LIST OF References

Blackledge et al 1989

Blackledge G, Lawton F, Redman C, Kelly K.  Response of patients in Phase II studies of chemotherapy in ovarian cancer: implications for patient treatment and the design of Phase II trials.  Br J Cancer 1989;59(4):650-3.

Cella et al 2002

Cella DF, Eton DT, Lai JS, Peterman AH, Merkel DE. Combining anchor and distribution-based methods to derive minimal clinically important differences on the functional assessment of cancer therapy (FACT) anaemia and fatigue scales. J Pain Symptom Manage. 2002;24:547-61.

Collett 1994

Collett D. Modelling survival data in medical research Chapman & Hall/CRC 1994.

Colombo et al 2010

Colombo N**,** Peiretti M, Parma G, Lapresa M, Mancari R, Carinelli S, et al. Newly diagnosed and relapsed epithelial ovarian carcinoma: ESMO Clinical Practice Guidelines for diagnosis, treatment and follow up. Ann Oncol 2010; 21 (Suppl 5) v23-v30.

Eisenhauer et al 1997

Eisenhauer EA, Vermorken JB, van Glabbeke M.  Predictors of response to subsequent chemotherapy in platinum pre-treated ovarian cancer: a multivariate analysis of 704 patients.  Ann Oncol 1997;8(10):963-968.

Eisenhauer et al 2009

Eisenhauer EA, Therasse P, Bogaerts J, et al.  New response evaluation criteria in solid tumours: Revised RECIST guideline (version 1.1).  Eur J Cancer 2009;45(2):228-247.

Fong et al 2009

Fong PC, Boss DS, Yap TA, Tutt A, Wu P, Mergui-Roelvink M, et al. Inhibition of poly(ADP-ribose) polymerase in tumors from *BRCA* mutation carriers. N Engl J Med. 2009;361(2):123-34.

Gore et al 1990

Gore ME, Fryatt I, Wiltshaw E, Dawson T.  Treatment of relapsed carcinoma of the ovary with cisplatin or carboplatin following initial treatment with these compounds.  Gynecol Oncol 1990;36(2):207-11.

Hay et al 2009

Hay T, Matthews JR, Pietzka L, Lau A, Cranston A, Nygren AO, et al. Poly(ADP-ribose) polymerase-1 inhibitor treatment regresses autochthonous *BRCA*2/p53-mutant mammary tumors in vivo and delays tumor relapse in combination with carboplatin. Cancer Res.2009;69(9):3850-5.

Helleday 2011

Helleday T. The underlying mechanism for the PARP and *BRCA* synthetic lethality: Clearing up the misunderstandings. Molecular Oncology 2011; 5: 387-393.

Ledermann et al 2012

Ledermann J, Harter P, Gourley C. Olaparib Maintenance Therapy in Platinum-Sensitive Relapsed Ovarian Cancer. N Engl J Med 2012;366:1382-92.

Ledermann et al 2016

Ledermann J, Harter P, Gourley C. Overall survival in patients with platinum-sensitive recurrent serous ovarian cancer receiving olaparib maintenance monotherapy: an updated analysis from a randomized, placebo-controlled, double-blind, phase 2 trial. Lancet Oncol. 2016;17(11):1579-89.

Markman et al 1991

Markman M, Reichman B, Hakes T, Jones W, Lewis JL Jr, Rubin S, et al.  Responses to second-line cisplatin-based intraperitoneal therapy in ovarian cancer: influence of a prior response to intravenous cisplatin.  J Clin Oncol 1991;9(10):1801-5.

McGuire et al 1996

McGuire WP, Hoskins WJ, Brady MF, Kucera PR, Partridge EE, Look KY, et al.  Cyclophosphamide and cisplatin compared with paclitaxel and cisplatin in patients with stage III and stage IV ovariancancer.  N Engl J Med 1996;334:1-6.

Murai et al

Murai J, Huang SN, Das BB, Renaud A, et al. Trapping of PARP1 and PARP2 by Clinical PARP Inhibitors. AACR; 72(21); 5588–99.

NCCN Clinical Practice Guidelines in Oncology; Ovarian cancer version I.2013

www.nccn.com

NCI; *BRCA*1 and *BRCA*2: Cancer Risk and Genetic Testing

[http://www.cancer.gov/cancertopics/factsheet/Risk/*BRCA*](http://www.cancer.gov/cancertopics/factsheet/Risk/BRCA)

Pennington et al 2014

Pennington KP, Walsh T, Harrell MI, et al. Germline and somatic mutations in homologous recombination genes predict platinum response and survival in ovarian, fallopian tube, and peritoneal carcinomas. Clin Cancer Res 2014; 20: 764-775.

Richards et al 2008

Richards SC, Bale S, Bellissimo DB. ACMG recommendations for standards for interpretation and reporting of sequence variations. *Genet Med* 2008; 10(4):294–300.

Konstantinopoulos et al 2015

Konstantinopoulos P A, Ceccaldi R, Shapiro G I, et al. Homologous recombination deficiency: exploiting the fundamental vulnerability of ovarian cancer. Cancer discovery, 2015, 5(11): 1137-1154

Rottenberg et al 2008

Rottenberg S, Jaspers JE, Kersbergen A, van der Burg E, Nygren AO, Zander SA, et al. High sensitivity of *BRCA*1-deficient mammary tumors to the PARP inhibitor AZD2281 alone and in combination with platinum drugs. Proc Natl Acad Sci. 2008;105(44):17079-84.

Siegel et al 2014

Siegel R, Ma J, Zou Z, Jemal A. Cancer statistics, 2014. CA Cancer J Clin. 2014;64(1):9-29.

Yellen et al 1997

Yellen SB, Cella DF, Webster K, Blendowski C, Kaplan E. Measuring fatigue and other anaemia-related symptoms with the Functional Assessment of Cancer Therapy (FACT) measurement system. J Pain Symptom Manage. 1997;13:63-74.

Zeng et al 2015

Zeng HM, Zheng RS, Guo TM, et al. Cancer survival in China, 2003-2005: A population-based study. Int J Cancer. 2015;136:1921-30.

Appendix A Additional Safety Information

Further Guidance on the Definition of a Serious Adverse Event (SAE)

Life threatening

‘Life-threatening’ means that the patient was at immediate risk of death from the AE as it occurred or it is suspected that use or continued use of the product would result in the patient’s death. ‘Life-threatening’ does not mean that had an AE occurred in a more severe form it might have caused death (eg, hepatitis that resolved without hepatic failure).

Hospitalisation

Outpatient treatment in an emergency room is not in itself a serious AE, although the reasons for it may be (eg, bronchospasm, laryngeal oedema). Hospital admissions and/or surgical operations planned before or during a study are not considered AEs if the illness or disease existed before the patient was enrolled in the study, provided that it did not deteriorate in an unexpected way during the study.

Important medical event or medical intervention

Medical and scientific judgement should be exercised in deciding whether a case is serious in situations where important medical events may not be immediately life threatening or result in death, hospitalisation, disability or incapacity but may jeopardize the patient or may require medical intervention to prevent one or more outcomes listed in the definition of serious. These should usually be considered as serious.

Simply stopping the suspect drug does not mean that it is an important medical event; medical judgement must be used.

- Angioedema not severe enough to require intubation but requiring iv hydrocortisone treatment
- Hepatotoxicity caused by paracetamol (acetaminophen) overdose requiring treatment with N-acetylcysteine
- Intensive treatment in an emergency room or at home for allergic bronchospasm
- Blood dyscrasias (eg, neutropenia or anaemia requiring blood transfusion, etc) or convulsions that do not result in hospitalisation
- Development of drug dependency or drug abuse

A Guide to Interpreting the Causality Question

When making an assessment of causality consider the following factors when deciding if there is a ‘reasonable possibility’ that an AE may have been caused by the drug.

- Time Course. Exposure to suspect drug. Has the patient actually received the suspect drug? Did the AE occur in a reasonable temporal relationship to the administration of the suspect drug?
- Consistency with known drug profile. Was the AE consistent with the previous knowledge of the suspect drug (pharmacology and toxicology) or drugs of the same pharmacological class? Or could the AE be anticipated from its pharmacological properties?
- De-challenge experience. Did the AE resolve or improve on stopping or reducing the dose of the suspect drug?
- No alternative cause. The AE cannot be reasonably explained by another aetiology such as the underlying disease, other drugs, other host or environmental factors.
- Re-challenge experience. Did the AE reoccur if the suspected drug was reintroduced after having been stopped? AstraZeneca would not normally recommend or support a re-challenge.
- Laboratory tests. A specific laboratory investigation (if performed) has confirmed the relationship.

In difficult cases, other factors could be considered such as:

- Is this a recognized feature of overdose of the drug?
- Is there a known mechanism?

Causality of ‘related’ is made if following a review of the relevant data, there is evidence for a ‘reasonable possibility’ of a causal relationship for the individual case. The expression ‘reasonable possibility’ of a causal relationship is meant to convey, in general, that there are facts (evidence) or arguments to suggest a causal relationship.

The causality assessment is performed based on the available data including enough information to make an informed judgment. With limited or insufficient information in the case, it is likely that the event(s) will be assessed as ‘not related’.

Causal relationship in cases where the disease under study has deteriorated due to lack of effect should be classified as no reasonable possibility.

Appendix B Actions Required in Cases of Increases in Liver

Biochemistry and Evaluation of Hy’s Law

Introduction

This Appendix describes the process to be followed in order to identify and appropriately report cases of Hy’s Law. It is not intended to be a comprehensive guide to the management of elevated liver biochemistries.

During the course of the study the Investigator will remain vigilant for increases in liver biochemistry. The investigator is responsible for determining whether a patient meets potential Hy’s Law (PHL) criteria at any point during the study.

The Investigator participates, together with AstraZeneca clinical project representatives, in review and assessment of cases meeting PHL criteria to agree whether Hy’s Law (HL) criteria are met. HL criteria are met if there is no alternative explanation for the elevations in liver biochemistry other than Drug Induced Liver Injury (DILI) caused by the Investigational Medicinal Product (IMP).

The Investigator is responsible for recording data pertaining to PHL/HL cases and for reporting Adverse Events (AE) and Serious Adverse Events (SAE) according to the outcome of the review and assessment in line with standard safety reporting processes.

Definitions

Potential Hy’s Law (PHL)

Aspartate Aminotransferase (AST) or Alanine Aminotransferase (ALT) ≥ 3x Upper Limit of Normal (ULN) **together with** Total Bilirubin (TBL) ≥ 2xULN at any point during the study following the start of study medication irrespective of an increase in Alkaline Phosphatase (ALP).

Hy’s Law (HL)

AST or ALT ≥ 3x ULN **together with** TBL ≥ 2xULN, where no other reason, other than the IMP, can be found to explain the combination of increases, eg, elevated ALP indicating cholestasis, viral hepatitis, another drug.

For PHL and HL the elevation in transaminases must precede or be coincident with (i.e. on the same day) the elevation in TBL, but there is no specified timeframe within which the elevations in transaminases and TBL must occur.

Identification of Potential Hy’s Law Cases

In order to identify cases of PHL it is important to perform a comprehensive review of laboratory data for any patient who meets any of the following identification criteria in isolation or in combination:

- ALT ≥ 3xULN
- AST ≥ 3xULN
- TBL ≥ 2xULN

When the identification criteria are met from central or local laboratory results the Investigator will without delay:

- Determine whether the patient meets PHL criteria (see Definitions within this Appendix for definition) by reviewing laboratory reports from all previous visits (including both central and local laboratory results)

The Investigator will without delay review each new laboratory report and if the identification criteria are met will:

- Notify the AstraZeneca representative
- Determine whether the patient meets PHL criteria (see Definitions within this Appendix for definition) by reviewing laboratory reports from all previous visits
- Promptly enter the laboratory data into the laboratory CRF

Follow-up

Potential Hy’s Law Criteria not met

If the patient does not meet PHL criteria the Investigator will:

- Inform the AstraZeneca representative that the patient has not met PHL criteria.
- Perform follow-up on subsequent laboratory results according to the guidance provided in the Clinical Study Protocol.

Potential Hy’s Law Criteria met

If the patient does meet PHL criteria the Investigator will:

- Determine whether PHL criteria were met at any study visit prior to starting study treatment (See Actions Required When Potential Hy’s Law Criteria are Met Before and After Starting Study Treatment)
- Notify the AstraZeneca representative who will then inform the central Study Team

The Study Physician contacts the Investigator, to provide guidance, discuss and agree an approach for the study patients’ follow-up and the continuous review of data. Subsequent to this contact the Investigator will:

- Monitor the patient until liver biochemistry parameters and appropriate clinical symptoms and signs return to normal or baseline levels, or as long as medically indicated
- Investigate the etiology of the event and perform diagnostic investigations as discussed with the Study Physician.
- Complete the three Liver CRF Modules as information becomes available
- If at any time (in consultation with the Study Physician) the PHL case meets serious criteria, report it as an SAE using standard reporting procedures

Review and Assessment of Potential Hy’s Law Cases

The instructions in this Section should be followed for all cases where PHL criteria are met.

No later than 3 weeks after the biochemistry abnormality was initially detected, the Study Physician contacts the Investigator in order to review available data and agree on whether there is an alternative explanation for meeting PHL criteria other than DILI caused by the IMP.

According to the outcome of the review and assessment, the Investigator will follow the instructions below.

If there is an agreed alternative explanation for the ALT or AST and TBL elevations, a determination of whether the alternative explanation is an AE will be made and subsequently whether the AE meets the criteria for a SAE:

- If the alternative explanation is **not** an AE, record the alternative explanation on the appropriate CRF
- If the alternative explanation is an AE/SAE, record the AE /SAE in the CRF accordingly and follow the AZ standard processes

If it is agreed that there is **no** explanation that would explain the ALT or AST and TBL elevations other than the IMP:

- Report an SAE (report term ‘Hy’s Law’) according to AstraZeneca standard processes.
- The ‘Medically Important’ serious criterion should be used if no other serious criteria apply
- As there is no alternative explanation for the HL case, a causality assessment of ‘related’ should be assigned.

If, there is an unavoidable delay, of over 3 weeks, in obtaining the information necessary to assess whether or not the case meets the criteria for HL, then it is assumed that there is no alternative explanation until such time as an informed decision can be made:

- Report an SAE (report term ‘Potential Hy’s Law’) applying serious criteria and causality assessment as per above
- Continue follow-up and review according to agreed plan. Once the necessary supplementary information is obtained, repeat the review and assessment to determine whether HL criteria are met. Update the SAE report according to the outcome of the review

Actions Required When Potential Hy’s Law Criteria are Met Before and After Starting Study Treatment

This section is applicable to patients with liver metastases who meet PHL criteria on study treatment having previously met PHL criteria at a study visit prior to starting study treatment.

At the first on study treatment occurrence of PHL criteria being met the Investigator will:

- Determine if there has been a significant change in the patients’ condition^#^ compared with the last visit where PHL criteria were met^#^
- If there is no significant change no action is required
- If there is a significant change notify the AstraZeneca representative, who will inform the central Study Team, then follow the subsequent process described in Potential Hy’s Law Criteria met of this Appendix

^#^ A ‘significant’ change in the patient’s condition refers to a clinically relevant change in any of the individual liver biochemistry parameters (ALT, AST or total bilirubin) in isolation or in combination, or a clinically relevant change in associated symptoms. The determination of whether there has been a significant change will be at the discretion of the Investigator, this may be in consultation with the Study Physician if there is any uncertainty.

Actions Required for Repeat Episodes of Potential Hy’s Law

This section is applicable when a patient meets PHL criteria on study treatment and has already met PHL criteria at a previous on study treatment visit.

The requirement to conduct follow-up, review and assessment of a repeat occurrence(s) of PHL is based on the nature of the alternative cause identified for the previous occurrence.

The investigator should determine the cause for the previous occurrence of PHL criteria being met and answer the following question:

- Was the alternative cause for the previous occurrence of PHL criteria being met found to be the disease under study e.g. chronic or progressing malignant disease, severe infection or liver disease, << or did the patient meet PHL criteria prior to starting study treatment and at their first on study treatment visit as described in Actions Required When Potential Hy’s Law Criteria are Met Before and After Starting Study Treatment >>?

If No: follow the process described in Potential Hy’s Law Criteria met of this Appendix

If Yes:

Determine if there has been a significant change in the patient’s condition^#^ compared with when PHL criteria were previously met

- If there is no significant change no action is required
- If there is a significant change follow the process described in this Appendix

^#^ A ‘significant’ change in the patient’s condition refers to a clinically relevant change in any of the individual liver biochemistry parameters (ALT, AST or total bilirubin) in isolation or in combination, or a clinically relevant change in associated symptoms. The determination of whether there has been a significant change will be at the discretion of the Investigator; this may be in consultation with the Study Physician if there is any uncertainty.

References

FDA Guidance for Industry (issued July 2009) ‘Drug-induced liver injury: Premarketing clinical evaluation’: <http://www.fda.gov/downloads/Drugs/GuidanceComplianceRegulatoryInformation/Guidances/UCM174090.pdf>

Appendix C Acceptable Birth Control Methods

Olaparib is regarded as a compound with medium/high foetal risk.

Women of childbearing potential and their partners, who are sexually active, must agree to the use one of highly effective forms of contraception and their partners must use a male condom (as listed below). This should be started from the signing of the informed consent and continue throughout the period of taking study treatment and for at least 6month after last dose of study drug(s), or they must totally/truly abstain from any form of sexual intercourse (see below).

**Acceptable Non-hormonal birth control methods include:**

- Total/True abstinence: When the subject refrains from any form of sexual intercourse and this is in line with their usual and/or preferred lifestyle; this must continue for the total duration of the trial and for at least 1 month after the last dose of study drug. [Periodic abstinence (e.g., calendar, ovulation, symptothermal, post-ovulation methods, or declaration of abstinence solely for the duration of a trial) and withdrawal are not acceptable methods of contraception]
- Vasectomised sexual partner PLUS male condom. With participant assurance that partner received post-vasectomy confirmation of azoospermia.
- Tubal occlusion PLUS male condom
- IUD PLUS male condom. Provided coils are copper-banded

Acceptable hormonal methods:

- Normal and low dose combined oral pills PLUS male condom
- Cerazette (desogestrel) PLUS male condom. Cerazette is currently the only highly efficacious progesterone based pill.
- Hormonal shot or injection (eg., Depo-Provera) PLUS male condom
- Etonogestrel implants (e.g., Implanon, Norplant) PLUS male condom
- Norelgestromin / EE transdermal system PLUS male condom
- Intrauterine system [IUS] device (eg., levonorgestrel releasing IUS -Mirena®) PLUS male condom
- Intravaginal device (e.g., EE and etonogestrel) PLUS male condom
