# Supplementary material for: Exploratory biomarker analysis in the phase III L-MOCA study of olaparib maintenance therapy in patients with platinum-sensitive relapsed ovarian cancer
Source: BMC Med. 2024 May 16;22:199. doi: 10.1186/s12916-024-03409-9 (PMC11100112; doi:10.1186/s12916-024-03409-9)
Supplement: Supplementary file 2 — Additional file 2: Figure S1. PD-L1 was mainly expressed on immune cells. Figure S2. The association between PD-L1 expression and olaparib efficacy in HRR mutation subgroups. Figure S3. The hypothesis of DNA damage accumulation in T cells. Table S1. ACTHRD assay experimental quality control parameters. Table S2. Summary of HRD testing results. Table S3. Summary of subject demographic and baseline factors by whether HRD was tested. Table S4. Summary of subjects with PD-L1 testing results. Table S5. Summary of demographics and baseline characteristics for different germline BRCA and PD-L1 subgroups. Table S6. Summary of demographics and baseline characteristics for different HRD and PD-L1 subgroups. Table S7. Summary of baseline pathology and extent of disease for different germline BRCA and PD-L1 subgroups. Table S8. Summary of baseline pathology and extent of disease for different HRD and PD-L1 subgroups.Table S9. Summary of previous anti-cancer therapy for ovarian cancer for different germline BRCA and PD-L1 subgroups. Table S10. Summary of previous anti-cancer therapy for ovarian cancer for different HRD and PD-L1 subgroups. Table S11. Summary of germline and somatic BRCA mutations in HRD-positive patients. Table S12. Concordance for detecting BRCA/HRR mutations in ctDNA and tumour samples. Table S13. PFS analysis in ctDNA-detected BRCA and HRR subgroups. Table S14. Summary of results of test of interaction. [file 12916_2024_3409_MOESM2_ESM.docx]

**Supplementary Materials**

**Exploratory Biomarker Analysis in the Phase III L-MOCA Study of Olaparib Maintenance Therapy in Patients with Platinum-Sensitive Relapsed Ovarian Cancer**

Huayi Li, Zikun Peng, Jianqing Zhu, Weidong Zhao, Yi Huang, Ruifang An, Hong Zheng, Pengpeng Qu, Li Wang, Qi Zhou, Danbo Wang, Ge Lou, Jing Wang, Ke Wang, Beihua Kong, Xing Xie, Rutie Yin, John Low, Abdul Malik Rozita, Lim Chun Sen, Yong Chee Meng, Kho Swee Kiong, Jihong Liu, Zhiqing Liang, Weiguo Lv, Yaping Zhu, Weiguo Hu, Wei Sun, Jingya Su, Qiqi Wang, Rongyu Zang, Ding Ma, Qinglei Gao

**Supplementary figures**


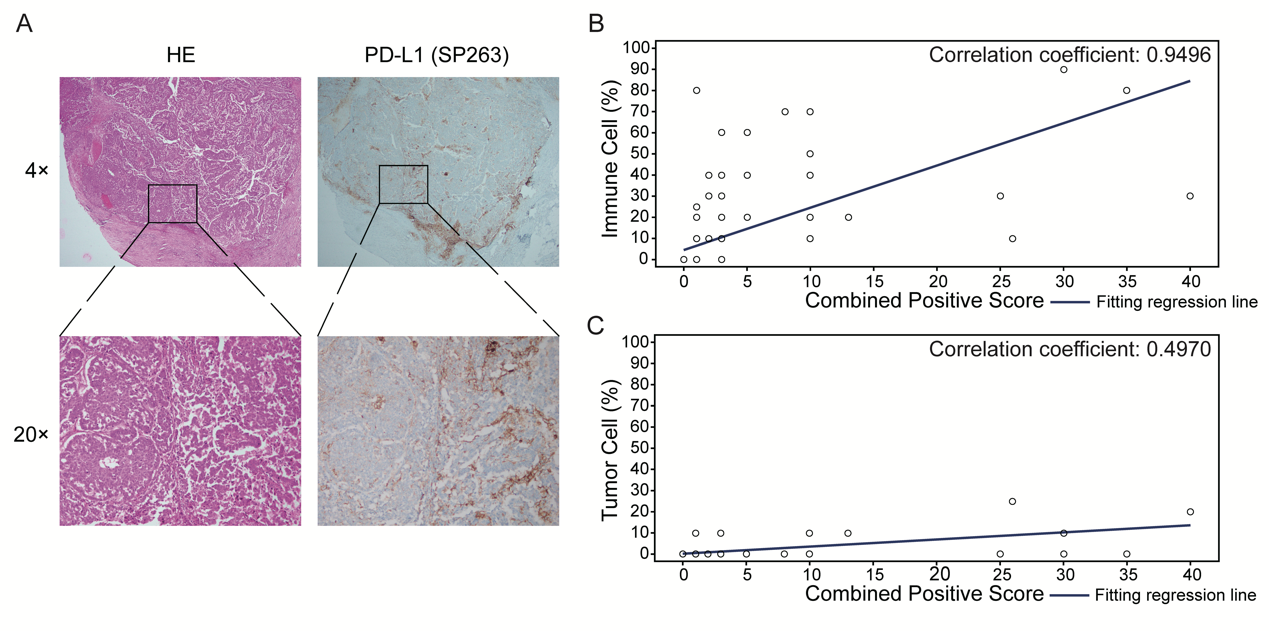


**Figure S1.** PD-L1 was mainly expressed on immune cells. **A** Representative fields of immunohistochemistry staining showing that PD-L1 was dominantly expressed on immune cells; **B** Correlation between PD-L1 expression on immune cells and combined positive score; **C** Correlation between PD-L1 expression on tumour cells and combined positive score; PD-L1 expression on immune cells contributed the most to combined positive score.


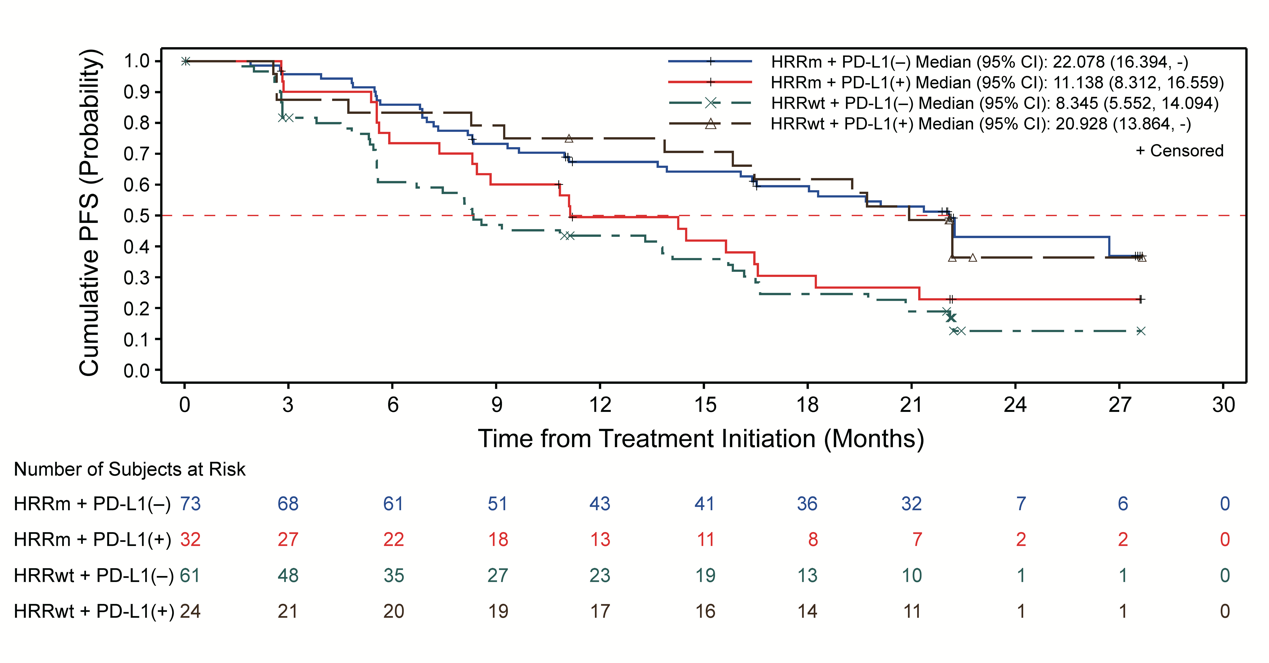


**Figure S2.** The association between PD-L1 expression and olaparib efficacy in HRR mutation subgroups. Kaplan-Meier plots of PFS analysis in HRRm PD-L1-negative, HRRm PD-L1-positive, HRRwt PD-L1-negative, and HRRwt PD-L1-positive patients. Abbreviations: PFS, progression-free survival; PD-L1, programmed cell death ligand 1; 95% CI, 95% confidence interval; HRRm, mutated homologous recombination repair genes; HRRwt, wild-type homologous recombination repair genes.


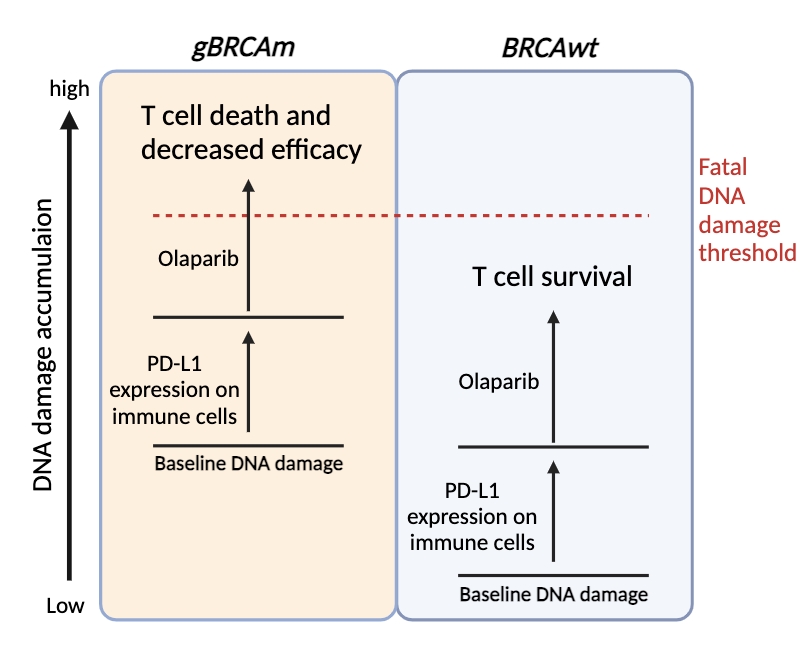


**Figure S3.** The hypothesis of DNA damage accumulation in T cells. T cells within patients with germline *BRCA1/2* mutations (*gBRCAm*) exhibit higher baseline DNA damage levels compared with T cells with wild-type *BRCA1/2*. PD-L1 expression on immune cells reflected IFNγ-induced adaptive regulation of PD-L1 expression and the presence of pre-existing immune responses including T cell activation. T cell activation could accumulate DNA damage within T cells. PARP inhibition leads to irreversible T cell death for *BRCA1/2*-deficient T cells, which could impair PARP inhibitors efficacy since PARP inhibitors partially depend on T cells to reject tumours. *BRCA1/2*-proficient T cells will survive PARP inhibitors treatment even when they are previously activated (PD-L1 expression on immune cells). Pre-existing T cell activation might indicate an immunoreactive niche that favours olaparib activities.**Supplementary Tables**

**Table S1.** ACTHRD assay experimental quality control parameters

| Parameter | Description |
| --- | --- |
| Tumour cell | Tumour cell ≥40% (30% ≤ Tumour cell <40% risk detection) |
| DNA | DNA concentration ≥3.4 ng/µL DNA total amount ≥60 ng DNA integrity (500 bp to 5 kbp) ≥15% |
| Library | Library total amount ≥2 nM × 10 μL  Library concentration ≥2 nM |
| Bioinformatics data | Q30 rate ≥80% Total base ≥0.8 G Mean depth for exonic region ≥1000x  Target base coverage at 200x for exonic region ≥90% Mean depth for SNP region ≥300x Target base coverage at 100x for SNP region ≥90% |

**Table S2.** Summary of HRD testing results

|  | Total (N=219) |
| --- | --- |
| HRD Tested |  |
| Yes, Testing Results: | 190 (86.8%) |
| Positive, specify: | 125 (65.8%) |
| *BRCAm* LOH high | 41 (32.8%) |
| *BRCAm* LOH low | 29 (23.2%) |
| *BRCAm* LOH Inconclusive^a^ | 1 (0.8%) |
| *BRCAwt* LOH high | 54 (43.2%) |
| Negative (*BRCAwt* LOH low) | 26 (13.7%) |
| Unknown^b^ | 39 (20.5%) |
| No | 29 (13.2%) |

Percentages of HRD was based on the number of subjects with sample submitted for inspection, regardless of whether there are effective test results. Other percentages are based on the number of subjects in analysis population. Subjects without biomarker informed consent with signed agreement are excluded from the analysis. Abbreviations: HRD, homologous recombination deficiency; *BRCAm*, mutated *BRCA1/2*; *BRCAwt*, wild-type *BRCA*; LOH, loss of heterozygosity.

^a^Tumour purity < 30% where only *BRCA* mutation could be tested.

^b^Unknown including unqualified samples that could not be tested and two samples where tumour purity was less than 30% with no *BRCA* mutation tested.

**Table S3.** Summary of subject demographic and baseline factors by whether HRD was tested

|  | Whether HRD Tested or Not | |
| --- | --- | --- |
|  | Yes (*N*=190) | No (*N*=29) |
| Age (years) |  |  |
| *n* (missing) | 190 (0) | 29 (0) |
| Median (Q1, Q3) | 54.0 (50.0, 62.0) | 55.0 (48.0, 59.0) |
| Age groups, *n* (%) |  |  |
| < 65 | 157 (82.6) | 27 (93.1) |
| >= 65 | 33 (17.4) | 2 (6.9) |
| Country, *n* (%) |  |  |
| China | 180 (94.7) | 24 (82.8) |
| Malaysia | 10 (5.3) | 5 (17.2) |
| FIGO Stage at Initial Diagnosis, *n* (%) |  |  |
| Stage I | 9 (4.7) | 0 |
| Stage II | 21 (11.1) | 5 (17.2) |
| Stage III | 132 (69.5) | 17 (58.6) |
| Stage IV | 23 (12.1) | 7 (24.1) |
| Unknown | 5 (2.6) | 0 |
| Response to Last Previous Platinum Based Chemotherapy |  |  |
| Complete Response | 72 (37.9) | 6 (20.7) |
| Partial Response | 118 (62.1) | 23 (79.3) |
| Time to Disease Progression on Second to Last Prior Platinum Based Chemotherapy^a^ |  |  |
| 6–12 months | 75 (39.5) | 13 (44.8) |
| >12 months | 114 (60.0) | 16 (55.2) |
| Disease at Baseline |  |  |
| Measurable | 46 (24.2) | 8 (27.6) |
| Non-Measurable | 144 (75.8) | 21 (72.4) |
| Prior Bevacizumab |  |  |
| With | 12 (6.3) | 1 (3.4) |
| Without | 178 (93.7) | 28 (96.6) |

Percentages are based on the number of subjects in analysis population. Subjects without biomarker informed consent with signed agreement are excluded from the analysis. Abbreviations: Q1, 25th percentile; Q3, 75th percentile; HRD, homologous recombination deficiency.

^a^One patient was found with Time to Disease Progression on Second to Last Prior Platinum Based Chemotherapy <6 months, who can not fall into either category.

**Table S4**. Summary of subjects with PD-L1 testing results

|  | Total (*N*=219) |
| --- | --- |
| PD-L1 Tested: |  |
| Yes, Testing Results: | 196 (89.5) |
| PD-L1 – Tumour Cell (%Positive) |  |
| <1% | 180 (91.8) |
| 1%-25% | 12 (6.1) |
| >25% | 0 |
| Unknown ^a^ | 4 (2.1) |
| PD-L1 – Immune Cell (%Positive) |  |
| <1% | 135 (68.9) |
| 1%-25% | 37 (18.9) |
| >25% | 19 (9.7) |
| Unknown ^a^ | 5 (2.5) |
| PD-L1 – Combined Positive Score |  |
| <1 | 142 (72.4) |
| ≥1 | 50 (25.5) |
| Unknown ^a^ | 4 (2.1) |
| No | 23 (10.5) |

Percentage of PD-L1 was based on the number of subjects with sample submitted for inspection, regardless of whether there are effective test results, other percentages are based on the number of subjects in analysis population. Abbreviations: PD-L1, programmed cell death ligand 1.

^a^Samples of four patients concluded unknown PD-L1 expression due to ineligible Formalin-Fixed Paraffin-Embedded (FFPE) tissue samples. An additional sample with the percentage of tumour-associated immune cells as 1% and the percentage of immune cells with PD-L1 positivity as <100% also concluded unknown results for PD-L1 expression in immune cells. Subjects without biomarker informed consent with signed agreement are excluded from the analysis.

**Table S5.** Summary of demographics and baseline characteristics for different germline *BRCA* and PD-L1 subgroups

|  | *gBRCAm* patients | | *BRCAwt* patients | |
| --- | --- | --- | --- | --- |
|  | IC<1% (*N*=52) | IC≥1% (*N*=24) | IC<1% (*N*=73) | IC≥1% (*N*=29) |
| Age (years) |  |  |  |  |
| n (missing) | 52 (0) | 24 (0) | 73 (0) | 29 (0) |
| Median (Q1, Q3) | 52.0 (47.5, 58.5) | 55.5 (50.5, 58.5) | 56.0 (50.0, 63.0) | 55.0 (51.0, 61.0) |
| Age groups, n (%) |  |  |  |  |
| <65 | 45 (86.5) | 19 (79.2) | 59 (80.8) | 25 (86.2) |
| ≥65 | 7 (13.5) | 5 (20.8) | 14 (19.2) | 4 (13.8) |
| Country, n (%) |  |  |  |  |
| China | 49 (94.2) | 23 (95.8) | 69 (94.5) | 26 (89.7) |
| Malaysia | 3 (5.8) | 1 (4.2) | 4 (5.5) | 3 (10.3) |
| BMI (kg/m^2^) |  |  |  |  |
| n (missing) | 52 (0) | 24 (0) | 73 (0) | 29 (0) |
| Median (Q1, Q3) | 24.1 (21.5, 26.7) | 24.7 (21.0, 27.4) | 23.2 (20.3, 25.4) | 25.1 (22.6, 26.6) |
| BMI groups, n (%) |  |  |  |  |
| ≤24 (kg/m^2^) | 26 (50.0) | 11 (45.8) | 46 (63.0) | 10 (34.5) |
| >24 (kg/m^2^) | 26 (50.0) | 13 (54.2) | 27 (37.0) | 19 (65.5) |

Percentages are based on the number of subjects in analysis population. Subjects without exploratory informed consent with signed agreement are excluded from the analysis. Abbreviations: *gBRCAm*, germline *BRCA1/2* mutations; *BRCAwt*, wild-type *BRCA1/2*. IC, immune cell; Q1, 25th percentile; Q3, 75th percentile; BMI, body mass index.

**Table S6.** Summary of demographics and baseline characteristics for different HRD and PD-L1 subgroups

|  | HRD-positive patients | | HRD-negative patients | |
| --- | --- | --- | --- | --- |
|  | IC<1% (*N*=81) | IC≥1% (*N*=41) | IC<1% (*N*=21) | IC≥1% (*N*=5) |
| Age (years) |  |  |  |  |
| n (missing) | 81 (0) | 41 (0) | 21 (0) | 5 (0) |
| Median (Q1, Q3) | 53.0 (50.0, 62.0) | 55.0 (50.0, 60.0) | 54.0 (47.0, 63.0) | 61.0 (61.0, 63.0) |
| Age groups, n (%) |  |  |  |  |
| <65 | 70 (86.4) | 34 (82.9) | 16 (76.2) | 4 (80.0) |
| ≥65 | 11 (13.6) | 7 (17.1) | 5 (23.8) | 1 (20.0) |
| Country, n (%) |  |  |  |  |
| China | 77 (95.1) | 38 (92.7) | 21 (100) | 5 (100) |
| Malaysia | 4 (4.9) | 3 (7.3) | 0 | 0 |
| BMI (kg/m^2^) |  |  |  |  |
| n (missing) | 81 (0) | 41 (0) | 21 (0) | 5 (0) |
| Median (Q1, Q3) | 23.2 (20.9, 25.4) | 25.6 (22.9, 27.1) | 22.9 (20.3, 24.3) | 22.6 (20.8, 25.1) |
| BMI groups, n (%) |  |  |  |  |
| ≤24 (kg/m^2^) | 49 (60.5) | 16 (39.0) | 14 (66.7) | 3 (60.0) |
| >24 (kg/m^2^) | 32 (39.5) | 25 (61.0) | 7 (33.3) | 2 (40.0) |

Percentages are based on the number of subjects in analysis population. Subjects without exploratory informed consent with signed agreement are excluded from the analysis. Abbreviations: HRD, homologous recombination deficiency; IC, immune cell; Q1, 25th percentile; Q3, 75th percentile; BMI, body mass index.

**Table S7.** Summary of baseline pathology and extent of disease for different germline *BRCA* and PD-L1 subgroups

|  | *gBRCAm* patients | | *BRCAwt* patients | |
| --- | --- | --- | --- | --- |
|  | IC<1% (*N*=52) | IC≥1% (*N*=24) | IC<1% (*N*=73) | IC≥1% (*N*=29) |
| FIGO Stage at Initial Diagnosis, n (%) |  |  |  |  |
| Stage I | 2 (3.8) | 2 (8.3) | 3 (4.1) | 1 (3.4) |
| Stage II | 5 (9.6) | 2 (8.3) | 6 (8.2) | 8 (27.6) |
| Stage III | 35 (67.3) | 18 (75.0) | 54 (74.0) | 19 (65.5) |
| Stage IV | 9 (17.3) | 2 (8.3) | 9 (12.3) | 0 |
| Unknown | 1 (1.9) | 0 | 1 (1.4) | 1 (3.4) |
| Primary Tumour Location, n (%) |  |  |  |  |
| Ovary | 47 (90.4) | 22 (91.7) | 68 (93.2) | 25 (86.2) |
| Fallopian Tube | 1 (1.9) | 1 (4.2) | 2 (2.7) | 4 (13.8) |
| Peritoneum | 1 (1.9) | 0 | 2 (2.7) | 0 |
| Other^a^ | 3 (5.8) | 1 (4.2) | 1 (1.4) | 0 |
| Histology Type, n (%) |  |  |  |  |
| Serous | 50 (96.2) | 24 (100) | 68 (93.2) | 28 (96.6) |
| Endometrioid | 2 (3.8) | 0 | 4 (5.5) | 1 (3.4) |
| Other | 0 | 0 | 1 (1.4) | 0 |
| Site of Metastatic Disease, n (%) |  |  |  |  |
| Brain/CNS | 2 (3.8) | 0 | 0 | 0 |
| Ascites | 0 | 0 | 1 (1.4) | 0 |
| Breast | 1 (1.9) | 0 | 0 | 0 |
| Gastrointestinal | 1 (1.9) | 1 (4.2) | 1 (1.4) | 1 (3.4) |
| Hepatic (including Gall Bladder) | 5 (9.6) | 1 (4.2) | 7 (9.6) | 1 (3.4) |
| Genitourinary | 0 | 0 | 2 (2.7) | 0 |
| Skin/Soft Tissue | 0 | 0 | 1 (1.4) | 0 |
| Bone and Locomotor | 1 (1.9) | 0 | 0 | 0 |
| Adrenal | 0 | 0 | 2 (2.7) | 0 |
| Lymph Nodes | 6 (11.5) | 1 (4.2) | 8 (11.0) | 3 (10.3) |
| Pericardial Effusion | 1 (1.9) | 0 | 0 | 0 |
| Peritoneum | 5 (9.6) | 3 (12.5) | 9 (12.3) | 2 (6.9) |
| Neck | 0 | 0 | 1 (1.4) | 0 |
| Other | 10 (19.2) | 6 (25.0) | 19 (26.0) | 5 (17.2) |
| Site of Locally advanced Disease, n (%) |  |  |  |  |
| Genitourinary | 1 (1.9) | 0 | 8 (11.0) | 0 |
| Skin/Soft Tissue | 0 | 0 | 1 (1.4) | 0 |
| Bone and Locomotor | 0 | 0 | 1 (1.4) | 0 |
| Lymph Nodes | 1 (1.9) | 1 (4.2) | 3 (4.1) | 2 (6.9) |
| Peritoneum | 1 (1.9) | 0 | 3 (4.1) | 1 (3.4) |
| Other | 12 (23.1) | 0 | 10 (13.7) | 6 (20.7) |
| Site of both Metastatic and Locally Advanced Disease, n (%) |  |  |  |  |
| Pleural Effusion | 1 (1.9) | 0 | 0 | 0 |
| Ascites | 1 (1.9) | 0 | 0 | 0 |
| Lymph Nodes | 0 | 0 | 1 (1.4) | 0 |
| Peritoneum | 1 (1.9) | 0 | 0 | 0 |
| Other | 1 (1.9) | 0 | 0 | 0 |
| Recurrence of earlier cancer, n (%) |  |  |  |  |
| Yes | 42 (80.8) | 24 (100) | 68 (93.2) | 28 (96.6) |
| No | 10 (19.2) | 0 | 4 (5.5) | 1 (3.4) |
| Missing | 0 | 0 | 1 (1.4) | 0 |

Percentages are based on the number of subjects in analysis population. Subjects without exploratory informed consent with signed agreement are excluded from the analysis. Abbreviations: *gBRCAm*, germline *BRCA1/2* mutations; *BRCAwt*, wild-type *BRCA1/2*; IC, immune cell; CNS, central nerve system.

^a^Other primary tumour locations include bilateral ovaries, double fallopian tubes, both ovary and fallopian tube, pelvic cavity, and bilateral adnexa.

**Table S8.** Summary of baseline pathology and extent of disease for different HRD and PD-L1 subgroups

|  | HRD-positive patients | | HRD-negative patients | |
| --- | --- | --- | --- | --- |
|  | IC<1% (*N*=81) | IC≥1% (*N*=41) | IC<1% (*N*=21) | IC≥1% (*N*=5) |
| FIGO Stage at Initial Diagnosis, n (%) |  |  |  |  |
| Stage I | 2 (2.5) | 3 (7.3) | 1 (4.8) | 0 |
| Stage II | 8 (9.9) | 7 (17.1) | 1 (4.8) | 2 (40.0) |
| Stage III | 60 (74.1) | 28 (68.3) | 17 (81.0) | 3 (60.0) |
| Stage IV | 10 (12.3) | 2 (4.9) | 2 (9.5) | 0 |
| Unknown | 1 (1.2) | 1 (2.4) | 0 | 0 |
| Primary Tumour Location, n (%) |  |  |  |  |
| Ovary | 79 (97.5) | 37 (90.2) | 19 (90.5) | 4 (80.0) |
| Fallopian Tube | 0 | 3 (7.3) | 1 (4.8) | 1 (20.0) |
| Peritoneum | 1 (1.2) | 0 | 0 | 0 |
| Other^a^ | 1 (1.2) | 1 (2.4) | 1 (4.8) | 0 |
| Histology Type, n (%) |  |  |  |  |
| Serous | 78 (96.3) | 41 (100) | 21 (100) | 5 (100) |
| Endometrioid | 3 (3.7) | 0 | 0 | 0 |
| Other | 0 | 0 | 0 | 0 |
| Site of Metastatic Disease, n (%) |  |  |  |  |
| Brain/CNS | 2 (2.5) | 0 | 0 | 0 |
| Ascites | 0 | 0 | 1 (4.8) | 0 |
| Hepatic (including Gall Bladder) | 6 (7.4) | 2 (4.9) | 4 (19.0) | 0 |
| Genitourinary | 1 (1.2) | 0 | 1 (4.8) | 0 |
| Skin/Soft Tissue | 1 (1.2) | 0 | 0 | 0 |
| Bone and Locomotor | 1 (1.2) | 0 | 0 | 0 |
| Adrenal | 2 (2.5) | 0 | 0 | 0 |
| Lymph Nodes | 6 (7.4) | 0 | 4 (19.0) | 2 (40.0) |
| Pericardial Effusion | 1 (1.2) | 0 | 0 | 0 |
| Peritoneum | 10 (12.3) | 3 (7.3) | 3 (14.3) | 0 |
| Neck | 1 (1.2) | 0 | 0 | 0 |
| Other | 17 (21.0) | 10 (24.4) | 4 (19.0) | 0 |
| Site of Locally Advanced Disease, n (%) |  |  |  |  |
| Genitourinary | 7 (8.6) | 0 | 2 (9.5) | 0 |
| Lymph Nodes | 3 (3.7) | 2 (4.9) | 0 | 0 |
| Peritoneum | 4 (4.9) | 1 (2.4) | 0 | 0 |
| Other | 15 (18.5) | 5 (12.2) | 3 (14.3) | 0 |
| Site of Both Metastatic and Locally Advanced Disease, n (%) |  |  |  |  |
| Ascites | 1 (1.2) | 0 | 0 | 0 |
| Peritoneum | 1 (1.2) | 0 | 0 | 0 |
| Other | 1 (1.2) | 0 | 0 | 0 |
| Recurrence of earlier cancer, n (%) |  |  |  |  |
| Yes | 68 (84.0) | 41 (100) | 20 (95.2) | 5 (100) |
| No | 13 (16.0) | 0 | 1 (4.8) | 0 |
| Missing |  |  |  |  |

Percentages are based on the number of subjects in analysis population. Subjects without exploratory informed consent with signed agreement are excluded from the analysis. Abbreviations: HRD, homologous recombination deficiency; IC, immune cell; CNS, central nerve system.

^a^Other primary tumour locations include bilateral ovaries, double fallopian tubes, both ovary and fallopian tube, pelvic cavity, and bilateral adnexa.

**Table S9.** Summary of previous anti-cancer therapy for ovarian cancer for different germline *BRCA* and PD-L1 subgroups

|  | *gBRCAm* patients | | *BRCAwt* patients | |
| --- | --- | --- | --- | --- |
|  | IC<1% (*N*=52) | IC≥1% (*N*=24) | IC<1% (*N*=73) | IC≥1% (*N*=29) |
| Subjects with Any Previous Anti-Cancer Therapy for Ovarian Cancer, n (%) | 52 (100) | 24 (100) | 73 (100) | 29 (100) |
| Therapy Class, n (%) |  |  |  |  |
| Immunotherapy | 0 | 0 | 1 (1.4) | 1 (3.4) |
| Hormonal Therapy | 0 | 0 | 0 | 1 (3.4) |
| Cytotoxic Chemotherapy | 11 (21.2) | 4 (16.7) | 11 (15.1) | 9 (31.0) |
| Systemic Therapy | 41 (78.8) | 20 (83.3) | 63 (86.3) | 22 (75.9) |
| Other | 14 (26.9) | 3 (12.5) | 22 (30.1) | 1 (3.4) |
| Prior lines of chemotherapy |  |  |  |  |
| 2 | 37 (71.2) | 14 (58.3) | 48 (65.8) | 22 (75.9) |
| 3 | 10 (19.2) | 9 (37.5) | 17 (23.3) | 4 (13.8) |
| 4 or greater | 5 (9.6) | 1 (4.2) | 8 (11.0) | 3 (10.3) |
| Duration of First Line Treatment (Days) |  |  |  |  |
| n (missing) | 51 (1) | 23 (1) | 72 (1) | 29 (0) |
| Median (Q1, Q3) | 149.0 (107.0, 184.0) | 134.0 (115.0, 193.0) | 138.5 (110.5, 173.0) | 159.0 (137.0, 187.0) |
| Duration of Second Line Treatment (Days) |  |  |  |  |
| n (missing) | 52 (0) | 24 (0) | 73 (0) | 29 (0) |
| Median (Q1, Q3) | 110.5 (80.5, 146.5) | 119.0 (88.0, 167.5) | 113.0 (79.0, 143.0) | 119.0 (85.0, 141.0) |
| Duration of Last Line Treatment (Days) |  |  |  |  |
| n (missing) | 52 (0) | 24 (0) | 73 (0) | 29 (0) |
| Median (Q1, Q3) | 116.5 (94.5, 143.0) | 120.5 (92.0, 165.0) | 99.0 (78.0, 134.0) | 121.0 (85.0, 146.0) |
| Response of last Platinum based Anti-Cancer Therapy for Ovarian Cancer prior to enrolment, n (%) |  |  |  |  |
| Complete Response | 24 (46.2) | 8 (33.3) | 22 (30.1) | 12 (41.4) |
| Partial Response | 28 (53.8) | 16 (66.7) | 51 (69.9) | 17 (58.6) |
| Classification of Platinum Sensitivity of Second to Last Previous Platinum Based Anti-Cancer Therapy for Ovarian Cancer, n (%) |  |  |  |  |
| PD<6 Months After  Completion Platinum Treatment | 0 | 0 | 1 (1.4) | 0 |
| PD≥6 months but ≤12  Months After Completion  Platinum Treatment | 23 (44.2) | 11 (45.8) | 24 (32.9) | 11 (37.9) |
| PD>12 Months After  Completion Platinum Treatment | 29 (55.8) | 13 (54.2) | 48 (65.8) | 18 (62.1) |

Percentages are based on the number of subjects in analysis population. Subjects without exploratory informed consent with signed agreement are excluded from the analysis. Abbreviations: g*BRCAm*, germline *BRCA1/2* mutations; *BRCAwt*, wild-type *BRCA1/2*; IC, immune cell; Q1, 25th percentile; Q3, 75th percentile; PD, progressive disease.

**Table S10.** Summary of previous anti-cancer therapy for ovarian cancer for different HRD and PD-L1 subgroups

|  | HRD-positive patients | | HRD-negative patients | |
| --- | --- | --- | --- | --- |
|  | IC<1% (*N*=81) | IC≥1% (*N*=41) | IC<1% (*N*=21) | IC≥1% (*N*=5) |
| Subjects with Any Previous Anti-Cancer Therapy for Ovarian Cancer, n (%) | 81 (100) | 41 (100) | 21 (100) | 5 (100) |
| Therapy Class, n (%) |  |  |  |  |
| Immunotherapy | 1 (1.2) | 0 | 0 | 1 (20.0) |
| Hormonal Therapy | 0 | 1 (2.4) | 0 | 0 |
| Cytotoxic Chemotherapy | 15 (18.5) | 13 (31.7) | 3 (14.3) | 0 |
| Systemic Therapy | 69 (85.2) | 31 (75.6) | 18 (85.7) | 5 (100) |
| Other | 24 (29.6) | 4 (9.8) | 6 (28.6) | 0 |
| Prior lines of chemotherapy |  |  |  |  |
| 2 | 57 (70.4) | 30 (73.2) | 14 (66.7) | 3 (60.0) |
| 3 | 13 (16.0) | 9 (22.0) | 6 (28.6) | 1 (20.0) |
| 4 or greater | 11 (13.6) | 2 (4.9) | 1 (4.8) | 1 (20.0) |
| Duration of First Line Treatment (Days) |  |  |  |  |
| n (missing) | 79 (2) | 40 (1) | 21 (0) | 5 (0) |
| Median (Q1, Q3) | 142.0 (109.0, 178.0) | 151.5 (118.0, 185.5) | 122.0 (111.0, 168.0) | 137.0 (110.0, 146.0) |
| Duration of Second Line Treatment (Days) |  |  |  |  |
| n (missing) | 81(0) | 41(0) | 21(0) | 5(0) |
| Median (Q1, Q3) | 119.0 (83.0, 149.0) | 111.0 (78.0, 158.0) | 96.0 (79.0, 129.0) | 139.0 (121.0, 140.0) |
| Duration of Last Line Treatment (Days) |  |  |  |  |
| n (missing) | 81(0) | 41(0) | 21(0) | 5(0) |
| Median (Q1, Q3) | 110.0 (79.0, 142.0) | 120.0 (86.0, 152.0) | 99.0 (84.0, 126.0) | 121.0 (105.0, 140.0) |
| Response of last Platinum based Anti-Cancer Therapy for Ovarian Cancer prior to enrolment, n (%) |  |  |  |  |
| Complete Response | 30 (37.0) | 16 (39.0) | 6 (28.6) | 2 (40.0) |
| Partial Response | 51 (63.0) | 25 (61.0) | 15 (71.4) | 3 (60.0) |
| Classification of Platinum Sensitivity of Second to Last Previous Platinum Based Anti-Cancer Therapy for Ovarian Cancer, n (%) |  |  |  |  |
| PD<6 Months After Completion Platinum Treatment | 1 (1.2) | 0 | 0 | 0 |
| PD≥6 months but ≤12 Months After Completion Platinum Treatment | 27 (33.3) | 15 (36.6) | 10 (47.6) | 3 (60.0) |
| PD>12 Months After Completion Platinum Treatment | 53 (65.4) | 26 (63.4) | 11 (52.4) | 2 (40.0) |

Percentages are based on the number of subjects in analysis population. Subjects without exploratory informed consent with signed agreement are excluded from the analysis. Abbreviations: HRD, homologous recombination deficiency; IC, immune cell; Q1, 25th percentile; Q3, 75th percentile; PD, progressive disease.

**Table S11.** Summary of germline and somatic *BRCA* mutation status in HRD-positive patients

|  | HRD-positive patients (*N*=125) |
| --- | --- |
| *gBRCAm* | 56 (44.8%) |
| *sBRCAm* | 8 (6.4%) |
| *BRCAwt* | 60 (48.0%) |
| Unknown | 2 (1.6%) |

Percentages are based on the number of HRD-positive subjects. One patient had both germline and somatic *BRCA1/2* mutations. Abbreviations: HRD, homologous recombination deficiency; *gBRCAm*, germline *BRCA1/2* mutations; *sBRCAm*, somatic *BRCA1/2* mutations; *BRCAwt*, wild-type *BRCA1/2*.

**Table S12.** Concordance for detecting *BRCA*/HRR mutations in ctDNA and tumour samples

|  | | Tumour samples results | | |
| --- | --- | --- | --- | --- |
|  | | *BRCAm* | *BRCAwt* | Total |
| ctDNA results | *BRCAm* | 91 | 1 | 92 |
|  | *BRCAwt* | 10 | 111 | 121 |
|  | Total | 101 | 112 | 213 |
|  | Percentage agreement (95% CI) | 90.1% PPA  (82.1–94.9%) | 99.1% NPA  (94.4–100.0%) | 94.8% OPA  (91.9–97.8%) |
|  | | Tumour samples results | | |
|  | | HRRm | HRRwt | Total |
| ctDNA results | HRRm | 103 | 2 | 105 |
|  | HRRwt | 16 | 92 | 108 |
|  | Total | 119 | 94 | 213 |
|  | Percentage agreement (95% CI) | 86.6% PPA  (78.8–91.9%) | 97.9% NPA  (91.8–99.6%) | 91.5% OPA  (87.8–95.3%) |

Abbreviations: *BRCAm*, *BRCA1/2* mutated; *BRCAwt*, *BRCA1/2* wild type; 95% CI, 95% confidence interval; ctDNA, circulating tumour DNA; HRRm, homologous recombination repair genes mutated; HRRwt, homologous recombination repair genes wild type; PPA, positive percentage agreement; NPA, negative percentage agreement; OPA, overall percentage agreement.

**Table S13.** PFS analysis in ctDNA-detected *BRCA* and HRR subgroups

|  | ctDNA *BRCAm* (*N*=92) | ctDNA *BRCAwt* (*N*=122) | ctDNA HRRm (*N*=105) | ctDNA HRRwt (*N*=109) |
| --- | --- | --- | --- | --- |
| Numbers of Patients, n (%) |  |  |  |  |
| With Event | 46 (50.0) | 86 (70.5) | 51 (48.6) | 81 (74.3) |
| PD | 44 (47.8) | 85 (69.7) | 49 (46.7) | 80 (73.4) |
| Death | 2 (2.2) | 1 (0.8) | 2 (1.9) | 1 (0.9) |
| Censored | 46 (50.0) | 36 (29.5) | 54 (51.4) | 28 (25.7) |
| PFS (Months) |  |  |  |  |
| Median (95% CI) | 22.2 (17.9, 26.7) | 11.1 (8.3, 15.8) | 22.2 (17.9, 26.7) | 10.9 (7.9, 15.8) |
| Progression-free Rate (%) (95% CI) |  |  |  |  |
| 6-month | 86.7 (77.8, 92.2) | 68.3 (59.0, 75.9) | 88.3 (80.3, 93.2) | 64.7 (54.7, 73.0) |
| 12-month | 70.7 (60.0, 79.0) | 49.1 (39.7, 57.9) | 70.1 (60.0, 78.0) | 47.3 (37.4, 56.5) |
| 24-month | 45.8 (32.5, 58.2) | 19.9 (11.8, 29.5) | 46.9 (34.7, 58.3) | 16.6 (8.8, 26.4) |

Percentages are based on the number of subjects in analysis population. Abbreviations: ctDNA, circulating tumour DNA; PD, progressive disease. PFS, progression-free survival; 95% CI, 95% confidence interval; *BRCAm*, *BRCA1/2* mutations; *BRCAwt*, *BRCA1/2* wild type; HRRm, mutated homologous recombination repair genes; HRRwt, wild-type homologous recombination repair genes.

**Table S14.** Summary of results of test of interaction.

|  | *p*-value of test of interaction |
| --- | --- |
| PD-L1 expression × germline *BRCA1/2* mutations | 0.0003 |
| PD-L1 expression × HRR mutations | 0.0004 |
| PD-L1 expression × HRD status | 0.4745 |

HRR, homologous recombination repair; HRD, homologous recombination deficiency.
